# Therapeutic Potential of Kuwanon G: From Bioactivities to Network-Level Mechanisms

**DOI:** 10.3390/molecules31132292

**Published:** 2026-07-01

**Authors:** Esra Aydemir, Beyzanur Şimşek, Ayşe Acar, A. Cansu Kilit, Elif Odabaş Köse

**Affiliations:** 1 Department of Biology, Faculty of Science, Akdeniz University, TR-07058 Antalya, Turkey; esra@akdeniz.edu.tr (E.A.); beyzanur.balkis@hotmail.com (B.Ş.); ayse.acar.office@gmail.com (A.A.); 2 Biomedical Device Technology Program, Department of Electronics and Automation, Technical Sciences Vocational School, Akdeniz University, TR-07058 Antalya, Turkey; acansukilit@akdeniz.edu.tr; 3 Medical Laboratory Program, Vocational School of Health Services, Akdeniz University, TR-07058 Antalya, Turkey

**Keywords:** Kuwanon G, *Morus*, isoprenylated flavonoids, bioactivities, multi-target therapeutics

## Abstract

Natural products like the isoprenylated flavonoid Kuwanon G (KWG), isolated primarily from *Morus alba*, offer promising pleiotropic effects against multifactorial diseases, overcoming the limitations of conventional single-target synthetic drugs. This study aims to systematically review the pharmacological activities of KWG and evaluate its underlying molecular mechanisms. A comprehensive literature review was integrated with network pharmacology, protein–protein interaction (PPI) profiling, and KEGG/GO pathway enrichment analyses to identify shared targets across different pathologies. Experimental data demonstrate that KWG exhibits antimicrobial, anti-inflammatory, antidiabetic, neuroprotective, anti-obesity, and anticancer properties. Bioinformatics analyses revealed that KWG exerts these effects by modulating core targets (e.g., TNF, IL-6, SRC, RELA) and key signaling pathways, including NF-κB, PI3K/AKT/mTOR, and Toll-like receptors, which govern inflammation, oxidative stress, and metabolic regulation. In conclusion, KWG is a potent, multi-target compound with significant therapeutic potential for managing chronic and infectious diseases. However, future structure–activity relationship studies and clinical trials are required to address its pharmacokinetic limitations, such as low bioavailability, to facilitate its clinical translation.

## 1. Introduction

Secondary metabolites derived from plants that possess biological activity have formed an indispensable foundation for the treatment of diseases. In public health applications, these compounds play a critical role not only in the treatment of diseases but also in the prevention of chronic diseases (preventive medicine), nutritional strategies (nutraceuticals), agriculture and cosmetic applications [1,2,3]. In light of the significant costs and adverse systemic effects associated with synthetic drugs, global health authorities are prompted to re-evaluate the pharmacological potential of natural products [4]. The conventional “one drug–one target” approach frequently fails for the management of multifactorial diseases, including cancer, diabetes, and neurodegenerative diseases. In this respect, natural products possess a complex structure that is “pre-validated” for interacting with biological systems in the evolutionary process and exhibit the ability to modulate multiple signaling pathways simultaneously (pleiotropic effect) [2,5]. Moreover, the inadequacy of currently available treatments has rendered natural products as alternative target molecules [6].

Plant bioactive compounds are primarily classified according to their chemical structure and biosynthetic origin into categories such as glycosides, alkaloids, terpenoids, anthraquinones, flavonoids, saponins, and tannins. Among these, flavonoids represent the most prevalent class of phenolic organic compounds found in plants [7,8]. Flavonoids exhibit a variety of functions, including the regulation of cell growth, attraction of pollinating insects, and protection against biotic and abiotic stresses such as pathogen infection and cold stress [8,9]. All flavonoids share a common structural element, characterized by the presence of two phenyl rings (A and B) linked to a heterocyclic ring (C, which contains an embedded oxygen atom). This carbon structure can be abbreviated as C6-C3-C6. The classification of flavonoids is determined by the substitution arrangement of heterocyclic rings, and flavonoids can be classified into seven distinct categories: flavonols, flavones, isoflavones, anthocyanidins, flavanones, flavanols, and chalcones [10,11]. The structural variation in most flavonoids results from the replacement of hydrogen ions at different positions with other groups, such as hydroxyl, methoxyl, and glycosyl. These changes in groups significantly affect the physicochemical properties and biological activities of flavonoids [9,11,12]. For instance, flavonoids are present in plants either in free form (aglycones) or bound to sugars, and the glycosylation of flavonoids significantly increases their water solubility and stability, thereby conferring specific biological activities [9].

Prenylated flavonoids, a subclass of flavonoids, are formed when a flavonoid structure is combined with a lipophilic prenyl side chain [13]. Prenylation potentiates the lipophilicity of flavonoids, thereby enhancing their affinity for biological membranes and their capacity to interact with target proteins [13,14,15]. Prenylation has been detected in a wide range of flavonoids, including chalcones, flavanones, flavones, flavonols, and isoflavones [14]. Prenylated flavonoids have been reported to possess various pharmacological properties, including estrogenic [16], cytotoxic [17], antimicrobial [18], anti-diabetic [19,20], anti-Alzheimer [19,21], antioxidant [22,23], anti-inflammatory [19,23], and anticancer activities [24]. These compounds have been documented to confer health benefits to humans by interacting with various cellular targets involved in significant cellular signaling pathways within the body. In recent decades, there has been significant interest in prenylated flavonoids from researchers and pharmaceutical companies for their potential application in drug discovery [13,15,25].

**Figure 1:**
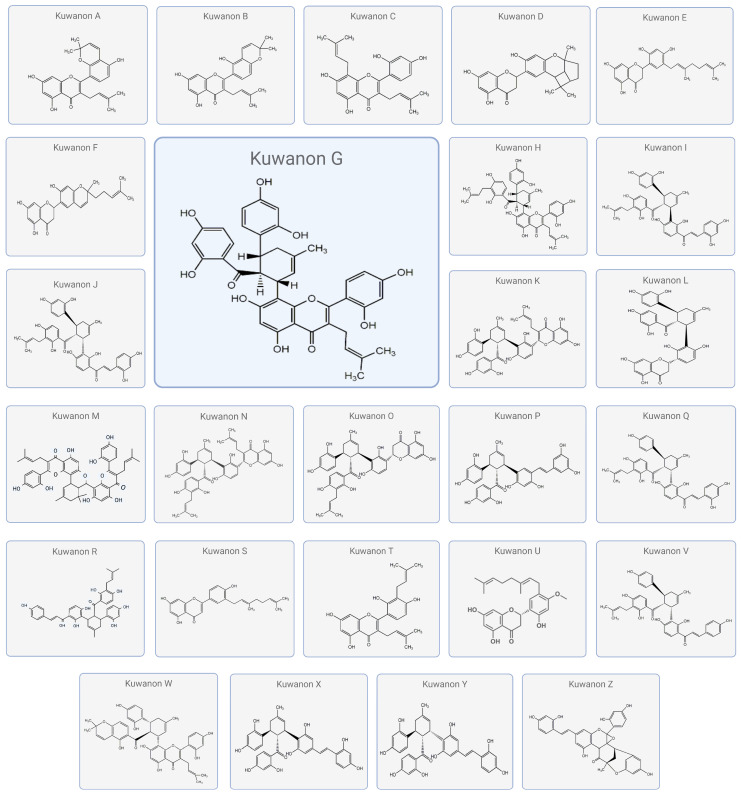
Chemical Structures of Kuwanon Types (Created in BioRender. https://BioRender.com/7nxf6m5, accessed on 7 May 2026).

Kuwanons are a class of naturally occurring isoprenylated flavonoids found in various Morus (mulberry) species of the Moraceae family, generally in roots, root barks, stem barks, and leaves [26,27]. The mulberry tree, which has been widely cultivated in Asia (China and Japan) and Europe for a long time, is generally used as a food source for silkworms. On the other hand, mulberry root bark is a crude drug called “Sang-Bai-Pi” used in traditional Chinese medicine as a diuretic, expectorant, laxative, anti-inflammatory, and antipyretic [28,29,30]. Since the 1970s, this plant has been extensively studied by researchers to identify and isolate its biologically active compounds. In conclusion, many compounds with unique structures, such as Diels–Alder type adducts (DAAs), benzofurans, stilbenes, flavonoids, and triterpenoids, have been isolated from Morus root bark (Mori Cortex Radicis) extract [31]. Mulberry Diels–Alder type adducts (MDAAs), characteristic components of mulberry trees, are a structurally unique group of naturally occurring phenolic compounds. MDDAs are mainly found in the root bark of the mulberry tree but have also been isolated from the stem bark, roots, branches, leaves and callus cultures of other plants in the Moraceae family. Their formation occurs via an enzymatic Diels–Alder reaction known as the [4 + 2] cycloaddition of a dehydroprenyl diene and a chalcone dienophile, forming a six-membered ring [31,32,33]. Structurally, kuwanons are members of a broader class of MDAAs. While the dienes of their structures are usually derived from monoterpenes, such as myrcene and β-trans-ocimene, or flavonoids, such as flavones, flavonols and flavanones, the dienophile in this class of Diels–Alder compounds consists only of a derivative of a chalcone. A multitude of kuwanon compounds have been reported from *Morus* spp., including kuwanon S, kuwanon G, kuwanon T, kuwanon H, kuwanon L, kuwanon X, and kuwanon C (Figure 1) [26].

**Figure 2:**
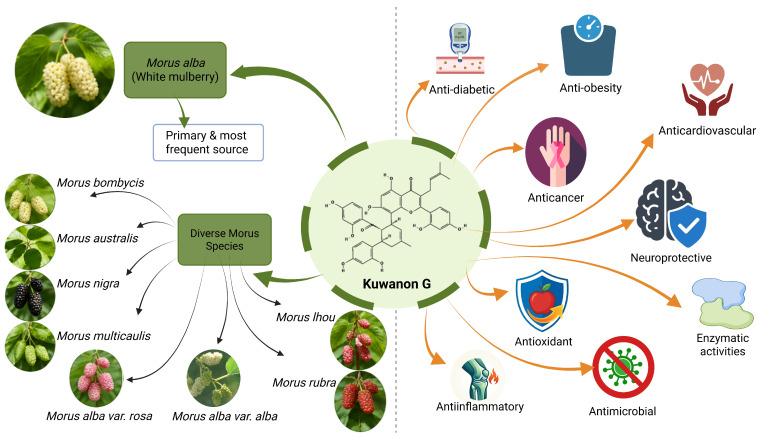
Origin and biological activities of Kuwanon G (Created in BioRender. https://BioRender.com/zh6a0u2, accessed on 5 June 2026).

Kuwanon G (KWG) is a naturally occurring MDAA first isolated from the root bark of *Morus alba* L., a species that is widely cultivated, particularly in Asia [34]. The chemical structure of KWG is responsible for its numerous biological activities, including its antimicrobial [35], antiasthmatic [36], anti-inflammatory [37], anti-diabetic [38] and neuroprotective activities (Figure 2) [39]. Although various biological activities of KWG have been identified, a comprehensive review article that systematically synthesizes these activities is not currently available. Therefore, this article plans to focus on the therapeutic activities of this compound, taking into account all studies conducted so far that have revealed the biological activities of KWG. Moreover, herein *in silico* approaches were employed to evaluate the potential molecular effects of KWG. The overlap between genes associated with KWG and those related to selected diseases was comparatively analyzed.

## 2. The Origin and Structure of KWG

KWG was first identified by Nomura and Fukai (1980) [34] as the primary active molecule behind the known hypotensive effect of root bark extracts of *M. alba* L. Analyses have demonstrated that albanin F [40], isolated from the shoot epidermis of the mulberry tree and exhibiting antifungal properties, and moracenin B [41], isolated from Mori Cortex Radicis and exhibiting hypotensive effects, are identical in structure to KWG [42]. To date, KWG has been isolated by many researchers from other *Morus* species (such as *Morus bombycis, Morus australis, and Morus nigra),* primarily *M. alba* (Table 1).

KWG is in the form of pale yellowish amorphous powder and has the molecular formula C_40_H_36_0_11_ [34,44]. It is a highly optically active molecule and characteristically exhibits a high negative specific rotation value ([α]D = −534°). Consistent with its flavonoid nature, it produced a positive reaction in the MgCl_2_ and ZnCl_2_ color tests. The presence of a shoulder at 280 nm in UV spectral analysis indicates that the molecule contains a “kuwanon C” skeleton/partial structure [28,34,40,89,90,91].

**Figure 3:**
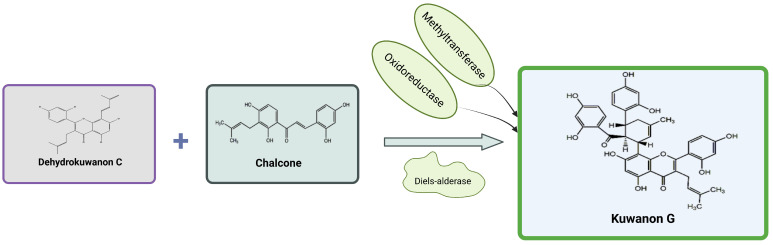
Synthesis of Kuwanon G (Created in BioRender. https://BioRender.com/u9fjvsx, accessed on 7 May 2026).

KWG is not a simple secondary metabolite but rather the product of a natural intermolecular [4 + 2] cycloaddition (Diels–Alder) reaction that occurs under enzymatic control within the plant system [28,90,91]. Classic MDAAs derived from the mulberry tree are classified into 8 different categories based on their structural characteristics. According to this classification system, KWG is the primary representative of the Type D (dehydroprenylflavone type MDDAs) class, which is formed by the combination of a calcon and a dehydroprenylflavone. The diene moiety in the structure of KWG is located at the C-8 position of ring A. Consequently, KWG is classified as Type DI, a subgroup of the Type D group, based on this characteristic [32]. During the biosynthetic process, the chalcone molecule, which contains a single unsaturated double bond, acts as a “dienophile”, while dehydrokuwanon C or an isoprenylated equivalent acts as a “diene”. These two molecules then combine to form a six-membered methylcyclohexene ring. The spatial arrangement of the three substituents on the methylcyclohexene ring (the chalcone and dehydrokuwanon C residues) is of great stereochemical importance (Figure 3). Based on its optical rotation direction and behavior in model syntheses, KWG has been identified as an “all-trans” addition product in terms of its relative configuration. This stereochemical configuration provides definitive scientific evidence that the enzyme-catalyzed Diels–Alder reaction proceeds via an “exo-addition” reaction mechanism within the plant [28,89,90,91,92]. According to the rules derived from the combination of X-ray analysis and circular dichroism (CD) spectroscopy findings, the absolute stereochemistry of the three chiral centers on the methylcyclohexene ring of KWG has been established in the scientific literature as 3″ R, 4″ R, 5″ S [90]. Partial synthesis studies conducted to prove the structure of the compound have confirmed the structural formula of KWG and its formation via the Diels–Alder mechanism [42]. Luo and colleagues completely synthesized KWG for the first time in a biomimetic manner in 2021 [93].

## 3. Biological Activities of KWG

### 3.1. Anti-Cardiovascular Effect

According to the WHO’s 2020 data, cardiovascular diseases are the leading cause of death worldwide. Arterial hypertension, one of the leading causes of cardiovascular disease, is directly or indirectly responsible for 47% of myocardial ischemia cases and 54% of stroke cases [94,95]. Nomura and Fukai (1980) [34], who first identified the compound KWG from the root bark of *M. alba*, also suggested that it possesses blood pressure-modulating properties. In intravenous tests conducted on rabbits, KWG induced a hypotensive response at a dose of 1 mg/kg. Oshima et al. (1980) [41] also found that moracenin B (KWG), isolated from mulberry root bark during the same period, exhibited similar effects. When administered intravenously in an *in vivo* mice model, moracenin B produced a blood pressure-lowering effect. These investigations indicate that KWG represents one of the active components contributing to the cardiovascular properties traditionally associated with the mulberry plant.

Atherosclerosis is a chronic inflammatory disease that affects the arteries, and its complications, particularly myocardial infarction and stroke, are the leading cause of death worldwide. The accumulation of lipids and inflammatory cells in the lesion area leads to the formation of atherosclerotic plaque, which grows inside the arteries and impairs their function. Macrophages, which transform into foam cells by engulfing oxidized low-density lipoproteins (ox-LDLs), play a crucial role in all stages of the disease, from lesion initiation to plaque rupture [96,97]. In a study investigating whether KWG affects macrophage foam cell formation *in vitro* and atherogenesis *in vivo*, the mRNA and protein levels of the ABCA1 and ABCG1 transporter proteins, which facilitate the efflux of excess cholesterol from macrophages, were significantly increased by KWG treatment in these *in vitro* models. KWG promoted cholesterol efflux by modulating the LXRα-ABCA1/ABCG1 signaling pathway. At the same time, KWG inhibited the transcription of proinflammatory cytokines such as TNF-α, IL-1β, IL-6, and IFN-γ at the cellular level by suppressing the activation of the NF-κB signaling pathway. Furthermore, in an *in vivo* model using ApoE−/− mice fed a high-fat diet, KWG administration reduced serum total cholesterol, low-density lipoprotein cholesterol, and circulating inflammatory cytokine levels in the blood. These preclinical findings suggest that KWG may serve as a pharmacological scaffold with the potential to modulate pathways associated with atherosclerosis [98].

The cytotoxic effects of a methanol extract obtained from the branch of *M. multicaulis* Perr. were evaluated on human umbilical vein endothelial cells (HUVECs) in an *in vitro* cell-based model at concentrations ranging from 10 to 100 µg/mL. The extract did not exhibit cytotoxic effects on HUVECs at concentrations of 10, 20, and 40 µg/mL but was cytotoxic at concentrations of 60, 80, and 100 µg/mL. To investigate whether the extract altered cell viability by preventing ROS-induced cellular damage caused by 80 µg/mL ox-LDL, the cells were incubated with the extract at concentrations of 10 µg/mL and 40 µg/mL. At both doses, the extract reduced the levels of ROS accumulated in the cells as a result of ox-LDL stimulation in these cellular assays. The authors suggested that the application of the extract may mitigate atherosclerotic progression at the cellular level by neutralizing oxidative damage caused by free radicals in endothelial cells. Furthermore, it is hypothesized that KWG, identified in the extract via UPLC-ESI-MS/MS analysis, may be the primary active component responsible for the extract’s anti-atherosclerotic effect [86].

The renin-angiotensin system (RAS) is one of the most important mechanisms in the body for regulating blood pressure and water and electrolyte balance [94,95]. The modulatory effects of the kuwanon-rich fraction obtained from mulberry root bark on RAS were investigated by Lee et al. (2024) [57] using enzymatic assays and *in vivo* animal models. According to the results, KWG and kuwanon H dose-dependently inhibited ACE (angiotensin-converting enzyme) in biochemical assays. At a concentration of 100 μg/mL, kuwanon H exhibited 2.2 times higher enzyme inhibition compared to KWG. It is suggested that this difference stems from the fact that, unlike KWG, kuwanon H contains two prenyl groups, and the position and number of these prenyl groups influence ACE inhibition. Subsequent *in vivo* animal model experiments demonstrated that the ethyl acetate fraction, which contains a high concentration of kuwanon, significantly reduced the increase in heart weight by 8% in mice fed a high-salt diet compared to the control group. Additionally, administration of this fraction modulated the RAS by reducing serum renin and angiotensinogen levels by 34% and 25%, respectively. These *in vitro* and *in vivo* data indicate that the kuwanon-rich fraction exhibits the potential to modulate pathways associated with hypertension by both directly inhibiting ACE and suppressing systemic renin/angiotensinogen release.

**Figure 4:**
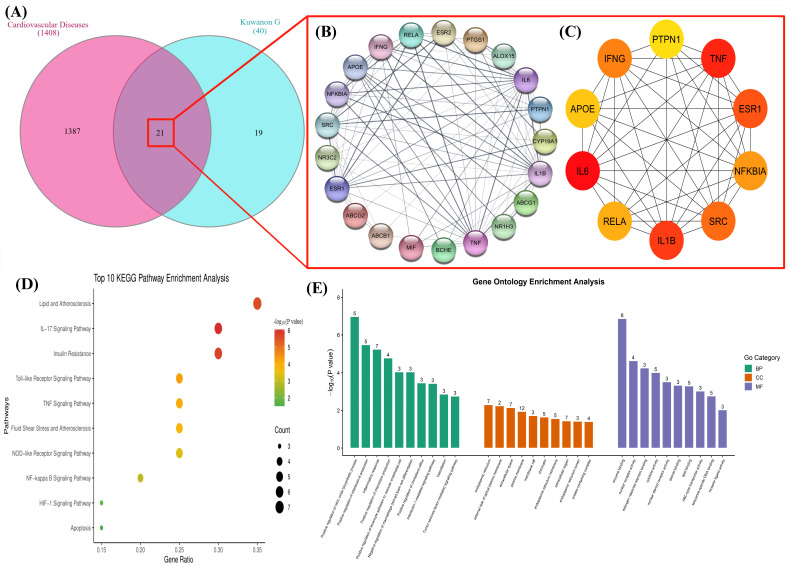
Network pharmacology analysis of Kuwanon G in cardiovascular diseases. (**A**) Venn diagram showing the overlap between cardiovascular disease targets (pink, *n* = 1408) and KWG targets (blue, *n* = 40); 21 common genes were identified. (**B**) The protein–protein interaction (PPI) network was constructed using the STRING database and visualized with Cytoscape. (**C**) Hub genes in the network are colored according to their degree values, ranging from red to yellow. (**D**) KEGG pathway enrichment analysis is shown as a dot plot, where dot size represents the number of genes and the color indicates significance (*p*-value). (**E**) GO enrichment analysis is shown as bar graphs for the categories of biological process (BP), cellular component (CC) and molecular function (MF).

The *in silico* network pharmacology results obtained indicate that KWG’s modulatory effects on cardiovascular diseases probably arise through multiple targets and multiple signaling pathways. In particular, pro-inflammatory cytokines such as IL-6, TNF, IL-1β, and IFN-γ, which stood out as core targets in the PPI network analysis, along with RELA, were modeled to be associated with KWG’s potential inhibitory effect on inflammatory processes To evaluate these *in silico* projections, existing literature offers valuable checkpoints; indeed, prior *in vitro* and *in vivo* studies have shown that KWG reduces the expression of cytokines such as TNF-α, IL-1β, IL-6, and IFN-γ by inhibiting the NF-κB signaling pathway. This parallels the functional importance of the hub genes identified computationally in our analysis. The TNF signaling, Toll-like receptor, and IL-17 signaling pathways, which were significantly enriched in the *in silico* KEGG pathway analysis, represent potential modulatory pathways related to the inflammatory mechanisms underlying atherosclerosis. Furthermore, the cytokine-mediated signaling, inflammatory response, and receptor-binding functions highlighted in the results of the computational GO analysis suggest KWG’s regulatory role in both inflammation and oxidative stress. Consistently, experimental cell-based assays indicate that KWG attenuates endothelial damage by reducing ROS levels and enhancing antioxidant defense. This finding is consistent with the computationally predicted biological processes in our analysis. However, genes such as *ESR1*, *SRC*, and *PTPN1* present in the *in silico* network suggest that KWG’s effects may involve not only inflammation but also cellular signaling and metabolic regulation processes. This multifaceted predictive effect highlights KWG’s preclinical potential in modulating pathways associated with cardiovascular diseases such as atherosclerosis and hypertension. The literature also reports that KWG exhibits antihypertensive effects through biochemically validated ACE inhibition and modulation of the renin-angiotensin system. In conclusion, the obtained computational network pharmacology data suggest that KWG has the capacity to modulate pathways associated with cardiovascular diseases through multiple targets, particularly inflammation, oxidative stress, and lipid metabolism, and this *in silico* evidence is closely aligns consistent with existing studies in the literature (Figure 4).

When the key genes identified in bioinformatics analyses as playing a role in the PPI network are evaluated in conjunction with findings from *in vitro* cell-based and *in vivo* animal experiments, it is suggested that the cardiovascular modulatory profile of KWG may be related to the regulation of inflammation, lipid metabolism, and vascular function. The identification of *TNF-α*, *IL-1β*, *IL-6*, and *IFN-γ* as core genes, the primary regulators of the inflammatory response associated with atherosclerosis, in an *in silico* network analysis, along with the observation that KWG reduces TNF-α, IL-1β, IL-6, and IFN -γ levels by suppressing the NF-κB signaling pathway in cellular models, highlights a notable alignment between bioinformatics findings and experimental results [98]. Similarly, the computational identification of RELA and NFKBIA, which are among the important regulators of NF-κB signaling, as core targets parallels the possible molecular mechanisms underlying the anti-inflammatory effect modulatory profile observed in cell and animal-based experimental studies. In terms of lipid metabolism, the *APOE*, *NR1H3*, and *ABCG1* genes in the network are particularly noteworthy. The lipid and atherosclerosis pathway, which was the most significantly enriched pathway in the KEGG analysis, mechanistically aligns with the potential anti-atherosclerotic properties of KWG observed in the studies. These experimental observations include the activation of the LXRα–ABCA1/ABCG1 signaling pathway in macrophages, increased cholesterol efflux, reduced intracellular lipid accumulation and attenuated foam cell formation in *in vitro*, alongside decreased serum cholesterol and inflammatory cytokine levels in *in vivo ApoE−/−* mouse models [98]. Additionally, the *in silico* enrichment of the shear stress and atherosclerosis pathway is consistent with the findings that extracts containing KWG reduce ROS formation induced by ox-LDL in cell-based models, thereby mitigating oxidative stress in HUVECs [86]. Furthermore, biological processes related to nitric oxide biosynthesis, vasodilation, and enzyme binding identified in the Gene Ontology analysis point to possible molecular mechanisms that may contribute to the observed cardiovascular-modulating and blood pressure-regulating potential of KWG. This is consistent with the fact that fractions rich in KWG and kuwanons exhibit inhibitory effects on ACE, modulate the RAS by reducing renin and angiotensinogen levels, and attenuate heart weight gain in mice fed a high-salt diet *in vivo* [57]. Consequently, when bioinformatic network analyses are evaluated together with *in vitro* and *in vivo* experimental data, KWG emerges as a valuable natural scaffold for investigating mechanisms relevant to the management of atherosclerosis and hypertension by potentially modulating multiple targets regulating lipid metabolism, inflammation, oxidative stress, and cardiovascular homeostasis. However, it is important to note that computational models provide valuable, hypothesis-generating insights into KWG’s multi-target profile. While key targets such as TNF-α and ACE have been empirically validated, the broader networks identified in KEGG and GO analyses represent potential modulatory pathways. These comprehensive *in silico* predictions will require more extensive *in vivo* and clinical studies to translate into validated physiological mechanisms.

To successfully advance these *in vivo* and *in silico* cardiovascular findings toward therapeutic translation, understanding KWG’s systemic behavior is essential. Intravenous tests in rabbits and mice demonstrate significant blood pressure-modulating parameters; however, oral administration introduces gastrointestinal stability barriers. Consequently, despite the clear multi-target pathways identified, the molecule’s true therapeutic window remains incomplete without systemic bioavailability and chronic toxicity data. Mapping the pharmacokinetics of specific intestinal metabolites of KWG is the critical next step to validate it as a reliable natural scaffold in cardiovascular medicine.

### 3.2. Anti-Diabetic Effect

Diabetes mellitus (DM) is a complex metabolic disorder characterized by hyperglycemia, a physiologically abnormal condition represented by persistently high blood sugar levels. Insulin is produced by pancreatic β cells and plays a vital role in regulating blood glucose levels. Insufficient insulin production or insulin resistance disrupts proper glucose homeostasis and leads to hyperglycemia. Hyperglycemia and its associated carbohydrate, fat, and protein metabolism disorders affect many organs in the body, including the eyes, kidneys, heart, and nerves, and disrupt their normal functioning [99,100]. α-glucosidase is a membrane-bound enzyme found in the epithelium of the small intestine. It catalyzes the hydrolytic breakdown of oligosaccharides into absorbable monosaccharides, thereby facilitating glucose absorption by the small intestine. One therapeutic approach used to treat diabetes is to slow down glucose absorption by inhibiting the α-glucosidase enzyme [101]. The protein tyrosine phosphatase 1B (PTP1B) enzyme is expressed primarily in tissues that regulate glucose metabolism, such as the liver, skeletal muscle, and adipose tissue, and it plays a role in the negative regulation of signaling mediated by tyrosine kinase receptors, particularly insulin and leptin receptors [102,103]. PTP1B has attracted significant attention in recent years due to its ability to attenuate insulin signaling and is currently recognized as a potential therapeutic target for metabolic syndrome, obesity, and diabetes [103]. In light of this objective, a substantial number of plant-based compounds are currently under evaluation for their α-glucosidase and PTP1B inhibitory activities.

In biochemical assays, KWG inhibited the PTP1B (Half Maximal Inhibitory Concentration (IC_50_), 2.26 ± 0.03 μM) more potently than the reference drug ursolic acid (IC_50_, 3.54 ± 0.06 μM). In the same enzymatic tests, KWG also inhibited the α-glucosidase (IC_50_, 2.35 ± 0.03 μM) more markedly than the reference agent acarbose (IC_50_, 119.16 ± 3.25 μM). Enzyme kinetics experiments confirmed that it exhibited a mixed-type inhibition against both enzymes. The *K*_i_ values were calculated for PTP1B and α-glucosidase are 1.98 μM and 2.51 μM, respectively. Subsequent molecular docking simulations were utilized to evaluate these enzymatic interactions at the molecular level. The docking findings suggested that KWG possesses a strong affinity for the allosteric site of PTP1B (−7.81 kcal/mol) and the catalytic site of α-glucosidase (−11.53 kcal/mol). To evaluate these parameters at the cellular level, KWG did not produce any significant reduction in cell viability of human hepatocellular carcinoma (HepG2) cells up to a concentration of 5 μM. At a dose of 10 μM, cell viability was measured at 85%. When applied to insulin-resistant HepG2 cells, KWG significantly increased the cells’ glucose uptake capacity in a dose-dependent manner. Western blot analyses indicated that KWG downregulates cellular levels of the PTP1B enzyme, which is oversynthesized in insulin-resistant cells. Although KWG could not completely reduce the protein expression to normal levels, it provided a steady attenuation in a dose-dependent manner (1.25, 2.5, and 5 μM). The results of the study indicate that KWG may be considered a valuable natural scaffold for investigating multi-target mechanisms relevant to modulating carbohydrate digestion and attenuating hepatic insulin receptor signaling parameters via PTP1B [58].

Zhao et al. (2018) [53] also reported that moracenin B (KWG) exhibited an inhibition of α-glucosidase by over 90% and α-amylase by over 80% in a biochromatographic assay. In quantitative analyses, this compound exhibited high inhibitory activity against α-glucosidase with an IC_50_ value of 2.80 ± 0.25 μM, which is lower than the IC_50_ value of 293.50 ± 34.42 μM measured for the standard agent acarbose. In the same biochemical tests, KWG exhibited an α-amylase inhibitory effect (IC_50_ = 2.85 ± 0.45 μM), which was similar to that of acarbose (IC_50_ = 1.51 ± 0.16 μM). Although the PTP1B enzyme demonstrated a weaker signal compared to other enzymes in the biochromatogram, quantitative analysis showed that KWG inhibited this enzyme with an IC_50_ value of 13.07 ± 1.74 μM (compared to an IC_50_ = 13.27 ± 1.00 μM for the positive control RK682). Furthermore, radical scavenging biochromatogram results suggested that KWG possesses prominent free radical scavenging activity potential, which may be attributed to its free hydroxyl groups.

Chen et al. (2018) [59] aimed to compare the chemical compositions of four different parts (root bark, branch, leaf, and fruit) of the *M. alba* and the inhibitory effects of their active components on the α-glucosidase enzyme. Among the 14 compounds tested via enzymatic evaluation in the study, KWG (IC_50_ = 3.45 ± 0.21 μg/mL) exhibited a prominent inhibitory activity against the α-glucosidase enzyme, following 1-deoxynojirimycin (DNJ). This inhibitory effect was found to be higher than that of acarbose (IC_50_ = 5.24 ± 0.47 μg/mL) in these biochemical setups. Spectrum-effect relationship analysis statistically indicated that KWG is one of the key components contributing to the *in vitro* enzymatic modulatory potential of the root bark. Molecular docking analyses have also suggested that KWG forms a stable complex by establishing seven hydrogen bonds with seven different amino acid residues (His348, Asp68, Arg439, Asp214, Arg212, Arg312, and His279) in the active site of α-glucosidase.

The extract obtained from the branches of *M. multicaulis* Perr. variety has been found to exhibit a dose-dependent inhibitory effect against the α-glucosidase (IC_50_, 1.90 ± 0.05 μg/mL) in enzymatic evaluation. LC-MS/MS results indicate that DNJ and KWG in the extract may be the primary compounds responsible for the inhibitory effect on α-glucosidase activity under these experimental conditions. Although the hypoglycemic potential of mulberry extracts has traditionally been attributed to DNJ, it has been determined that the amount of DNJ in the extract is only 0.65%. The authors state that, since the DNJ content is low, the extract contains other α-glucosidase-inhibiting components with greater activity than DNJ. Based on its high mass spectrum intensity and previous molecular binding studies, it is hypothesized that KWG may be the compound responsible for α-glucosidase inhibition [86].

Kwon et al. (2022) [38] found that among the bioactive compounds isolated from the ethyl acetate fraction of *M. alba* branches, KWG exhibited a higher affinity for the α-glucosidase enzyme than the standard drug acarbose (IC_50_ = 350.9 ± 17.94 μM), with an IC_50_ value of 1.44 ± 0.11 μM in enzymatic assays. In these biochemical tests, it also exhibited inhibitory activity against the PTP1B enzyme, with an IC_50_ value of 16.17 ± 0.29. At the same time, KWG was observed to attenuate the formation of advanced glycation end products (AGEs) and ONOO^−^ radicals, which trigger diabetic pathology, (IC_50_ values were 69.07 ± 1.49 μM and 6.35 ± 0.36 μM, respectively). Enzyme kinetic analyses demonstrated that KWG act as a mixed-type inhibitor for both α-glucosidase (*K*_i_ = 2.03 μM) and PTP1B (*K*_i_ = 12.41 μM) within these testing models. Molecular docking analyses suggested that KWG exhibits a much stronger thermodynamic affinity to the allosteric site (−8.89 kcal/mol) of the α-glucosidase compared to its catalytic site (−5.99 kcal/mol). The mixed inhibition profile of KWG modeled in experimental tests aligns with computational observations binding to both the catalytic (−6.26 kcal/mol) and allosteric sites (−7.11 kcal/mol) of the PTP1B.

To isolate active agents through advanced screening, Feng et al. (2022) [84] developed a “ligand fishing” strategy by covalently immobilizing the α-glucosidase onto magnetic nanoparticles (Fe_3_O_4_@SiO_2_) combined with a UPLC-MS/MS system. KWG was identified as one of the four main inhibitors successfully captured by an enzyme immobilized on magnetic nanoparticles. The IC_50_ value of KWG against the α-glucosidase was determined to be 2.83 ± 0.31 μM in enzymatic tests. The inhibitory effect exhibited by KWG is significantly stronger than that of DNJ, the reference alkaloid considered the primary hypoglycemic agent in mulberry extracts (IC_50_, 7.04 ± 0.82 μM), within these *in vitro* assays. Subsequent molecular docking analyses also suggested that KWG binds to the target protein with strong affinity (XP Gscore = −8.685) by forming hydrogen bonds via Asp242, Ser311, and Glu411 amino acid residues. Since the predicted binding regions of KWG were distinct from those of DNJ, the authors suggested a potential for evaluating synergistic mechanisms in future combination studies.

Hsu et al. (2022) [60], who investigated the anti-α-glucosidase activity of *M. alba*’s bioactive compounds, also reported that KWG exhibited an IC_50_ value of 7.00 ± 0.31 μM against the α-glucosidase in *in vitro* enzymatic assays. This effect is statistically approximately 77 times stronger than the effect shown by the positive control acarbose (IC_50_ = 540.38 ± 47.42 μM). As a result of *in silico* molecular docking and affinity analysis, KWG was modeled to interact with the active site of the enzyme with a binding energy of −8.5 kcal/mol, compared to −5.6 kcal/mol modeled for acarbose. Consequently, these comparative data indicate that KWG possesses a prominent biochemical capacity compared to synthetic acarbose in binding to the active site of the α-glucosidase enzyme and inhibiting enzyme activity under these experimental and computational conditions.

In a study investigating the inhibitory mechanisms of KWG and sanggenone D on α-glucosidase, KWG (IC_50_ = 3.83 × 10^−5^ mol/L) had higher inhibitory activity than sanggenone D (IC_50_ = 4.51 × 10^−5^ mol/L), but lower than acarbose (IC_50_ = 3.10 × 10^−7^ mol/L) in biochemical assays. Enzyme kinetic analyses demonstrated that KWG acts as a competitive inhibitor for the enzyme, meaning it directly competes with the substrate for the enzyme’s active site. According to fluorescence spectroscopy data, KWG rapidly interacted with the target enzyme and quenched its natural fluorescence via a static quenching mechanism. The FT-IR results demonstrated that the compound modified the secondary structure of the protein around the catalytic site after binding to the enzyme. Advanced molecular docking studies suggested that KWG exhibits a strong affinity for the enzyme, with a total binding energy of −13.83 kcal/mol. To investigate these mechanisms at the cellular level, an *in vitro* cell-based model using high-glucose employed HepG2 cells. Within this model, KWG reduced intracellular glucose, cholesterol, and triglyceride accumulation in HepG2 cells with a high glucose model more successfully than synthetic acarbose. According to Western blotting analyses, KWG statistically significantly increased the phosphorylation (p-AMPK form) of the AMPK enzyme, a key sensor of cellular energy metabolism. The activated AMPK signaling pathway was observed to upregulate the synthesis of the GLUT4 protein, an insulin-dependent glucose transporter. Additionally, KWG attenuated intracellular lipid accumulation by increasing the CPT-1 protein, which promotes the oxidation of fatty acids via the AMPK pathway. These findings provide valuable basic information regarding KWG’s potential as a natural α-glucosidase inhibitor scaffold under *in vitro* conditions [104].

Current research suggests a link between diabetes and gut microbiota. Compared to healthy individuals, patients with type 2 diabetes have been found to have disrupted gut flora, with a decrease in beneficial bacteria and an increase in pathogenic bacteria. Disruption of the gut microbiota can lead to pathogenic gram-negative bacteria releasing large amounts of endotoxins (lipopolysaccharide, LPS) in the gut. LPS is then transferred to the intestinal tissue, and the intestinal mucosal epithelial barrier may be damaged [105,106,107]. Disruption of intestinal integrity and increased intestinal permeability facilitate the passage of LPS from the intestinal lumen into the bloodstream, leading to metabolic endotoxemia [105,106].

Guo et al. (2016) [108] investigated whether KWG could mitigate LPS-induced epithelial cell damage, oxidative stress, pro-inflammatory cytokine release, and disruptions in tight junction proteins using an *in vitro* cell-based Caco-2 model. In these cell culture assays, LPS-damaged Caco-2 cells were incubated with 5, 50, 70, and 500 μM KWG for 24 h. KWG increased the viability of LPS-damaged Caco-2 cells in a concentration-dependent manner, with the most marked effect observed at 70 μM. When the KWG concentration exceeded 500 μM, an inhibitory effect on cell proliferation was observed. At the same time, KWG reduced the release of the pro-inflammatory cytokines IL-1β and TNF-α, pro-inflammatory cytokines whose release is induced by LPS, under these *in vitro* conditions. KWG alone was unable to reverse the LPS-induced decrease in superoxide dismutase (SOD), a protective enzyme, in Caco-2 cells. However, it significantly reduced malondialdehyde (MDA) levels, a strong indicator of lipid peroxidation, thereby restoring the SOD/MDA ratio, reflecting the overall antioxidant capacity of the cells. It was also found that the increase in LPS-induced reactive oxygen species (ROS) was reduced as a result of KWG administration. These evaluations indicated that LPS exposure decreased the expressions of intercellular proteins by as high as 80%. However, subsequent KWG administration restricted these alterations, significantly increasing the expression levels of the tight junction protein occludin and the intercellular adhesion molecule-1 (ICAM-1) compared to the LPS-treated control group, whereas KWG treatment did not reverse the LPS-induced decrease in zonula occludens (ZO)-1 parameters. Finally, the effect of the compound on the integrity and permeability of the intestinal epithelial barrier was evaluated. While LPS reduced the trans-epithelial electrical resistance (TEER) of the epithelial tissue by 10.86%, KWG administration increased TEER values, physically strengthening the barrier model *in vitro*. In parallel, KWG application prevented the leakage of the FITC-albumin molecule, which simulates intercellular leakage, through the epithelial barrier. The findings indicate that KWG may be a potent and innovative scaffold for investigating mechanisms relevant to diabetic endotoxemia and intestinal inflammation by enhancing cell viability, suppressing oxidative/inflammatory stress, and improving physical barrier permeability in cellular models.

**Figure 5:**
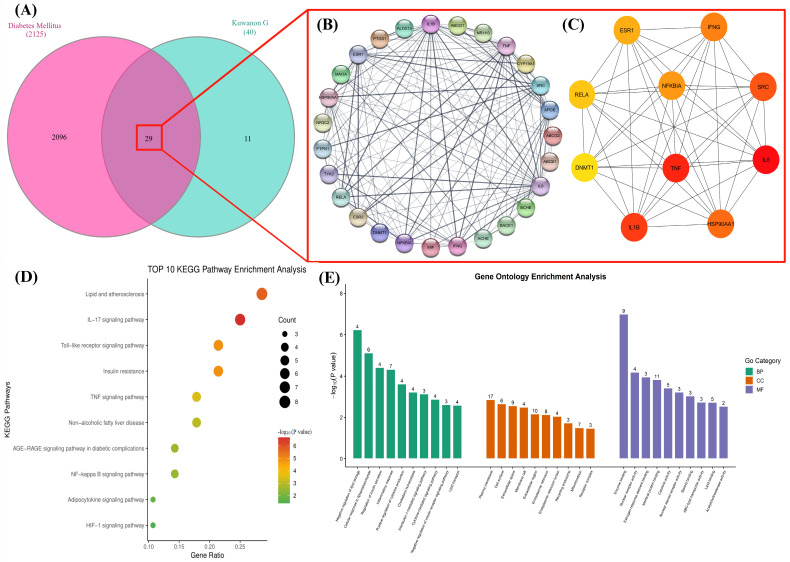
Network pharmacology analysis of Kuwanon G in diabetes mellitus. (**A**) Venn diagram showing the overlap between diabetes mellitus targets (pink, *n* = 2125) and KWG targets (blue, *n* = 40); 29 common genes were identified. (**B**) The protein–protein interaction (PPI) network was constructed using the STRING database and visualized with Cytoscape. (**C**) Hub genes in the network are colored according to their degree values, ranging from red to yellow. (**D**) KEGG pathway enrichment analysis is shown as a dot plot, where dot size represents the number of genes and the color indicates significance (*p*-value). (**E**) GO enrichment analysis is shown as bar graphs for the categories of biological process (BP), cellular component (CC) and molecular function (MF).

Bioinformatic and network analysis predicted a total of 29 common target genes at the intersection of diabetes mellitus-associated genes and KWG targets. KEGG pathway enrichment analysis suggested that these genes are concentrated in biological pathways traditionally linked to diabetes pathogenesis, primarily lipid and atherosclerosis, the TNF signaling pathway, the Toll-like receptor signaling pathway, the IL-17 signaling pathway, insulin resistance, and the AGE-RAGE signaling pathway. In the GO biological process category, the computational targets were found to be particularly concentrated in processes such as the inflammatory response, cellular response to lipopolysaccharides, positive regulation of cytokine production, and the regulation of insulin secretion. These computational predictions suggest that KWG holds a theoretical capacity to influence inflammation and metabolic processes that play a significant role in diabetes pathogenesis. These results from bioinformatic analysis are consistent with experimental findings reported in the literature. These computational predictions suggest that KWG holds a potential capacity to interact with inflammation and metabolic processes linked to diabetes pathogenesis. While these *in silico* pathway enrichments offer a useful multi-target roadmap, they serve as preliminary predictions that complement, rather than substitute, empirically validated biological mechanisms. Conceptually, these bioinformatic insights align well with experimental findings reported in the literature. Consequently, the generated networks indicate that KWG presents an encouraging multi-target profile for further exploratory studies, offering theoretical pathways to guide future research on inflammation, oxidative stress, and insulin resistance (Figure 5).

When these *in silico* network findings are evaluated together with experimental findings reported in the literature, it appears that KWG may act through multiple targets involved in diabetes-related processes. The computational identification of genes involved in diabetes pathogenesis, such as *TNF-α*, *IL-6*, *IL-1β*, *RELA*, *NFKBIA*, *IFN-γ*, *ESR1*, *SRC*, *HSP90AA1*, and *DNMT1*, among the common targets suggests that the potential modulatory properties of KWG may be related to inflammation, insulin resistance, and metabolic stress. This predicted linkage aligns conceptually with prior experimental studies. Indeed, *in vitro* cell-based models have shown that KWG increases glucose uptake capacity and significantly reduces overexpressed PTP1B levels in insulin-resistant HepG2 cells. Since PTP1B is a negative regulator of insulin receptor signaling, its suppression in cell culture may contribute to modulating insulin sensitivity parameters. The prominence of the insulin resistance pathway in our network analysis appears consistent with these cellular findings [58]. On the other hand, the *TNF-α*, *IL-6*, and *IL-1β* genes, which stood out in the network analysis, are among the molecules closely associated with inflammation and insulin resistance observed in diabetes. This *in silico* finding is also consistent with the results of the *in vitro* study conducted by Guo et al. (2016) [108]. In that cell-based study, it was reported that KWG application significantly reduced *TNF-α* and *IL-1β* levels in LPS-damaged Caco-2 cells. Furthermore, KWG was shown to improve oxidative stress markers, increase cell viability, and enhance the expression of proteins associated with intestinal barrier integrity under those *in vitro* conditions. These empirical cell-based observations conceptually match the inflammation-related targets and signaling pathways that stood out in our predictive network analysis. When these findings are considered together, it is suggested that the modulatory profile of KWG may not be limited solely to the regulation of glucose metabolism but may also be linked to mechanisms related to inflammation and intestinal barrier function within integrated *in vitro* and *in silico* frameworks.

Another notable result in the KEGG analysis is the computational enrichment of the AGE-RAGE signaling pathway. Since AGEs play a significant role in the development of diabetic complications, this predictive model parallels the experimental *in vitro* findings of Kwon et al. (2022) [38], who observed that KWG attenuates AGE formation. Taken together, these parallel insights suggest that KWG may be a candidate for further exploration regarding mechanisms linked to diabetes-related glycation processes in preclinical models. Concurrently, the prominent involvement of lipid and atherosclerosis, non-alcoholic fatty liver disease, and adipocytokine signaling pathways in the predictive models is also noteworthy. Given the close relationship between diabetes and obesity, these computational results suggest that the theoretical modulatory scope of KWG extends to influence processes related to lipid metabolism as well as carbohydrate metabolism. Indeed, empirical observations in high-glucose employed HepG2 cell models indicated that KWG reduces intracellular glucose, triglyceride, and cholesterol accumulation and increases AMPK phosphorylation and *CPT-1* expression. Therefore, an alignment can be observed between these bioinformatic projections and experimental *in vitro* findings in terms of processes related to glucose and lipid metabolism [104]. Overall, the core genes and enriched biological pathways identified in this *in silico* network pharmacology exploration exhibit a conceptual consistency with the cellular and enzymatic findings reported in the literature. These integrated preliminary insights indicate that KWG may serve as a valuable natural scaffold for investigating diverse biological mechanisms associated with type 2 diabetes across multiple molecular targets in future preclinical studies.

From a pharmacological perspective, translating these *in vitro* and *in silico* antidiabetic findings into therapy requires evaluating KWG’s metabolic profile. Although KWG strongly inhibits α-glucosidase and PTP1B in assays, its *in vivo* efficacy depends on its gastrointestinal stability. Metabolic profiling shows that KWG is extensively biotransformed by gut microbiota. Therefore, while local inhibition of intestinal α-glucosidase is highly plausible, its systemic effects—such as hepatic PTP1B downregulation and AMPK activation—are likely driven by its active metabolites rather than the parent compound. Furthermore, despite the established cytocompatibility in HepG2 cells and barrier enforcement in Caco-2 models, systemic bioavailability and toxicity parameters remain uncharacterized. Characterizing the pharmacokinetics of KWG’s specific intestinal metabolites is an essential next step to define its true multi-target potential in metabolic disorders.

### 3.3. Anti-Inflammatory/Anti-Allergic/Anti-Asthmatic Effects

Inflammatory lung diseases are generally caused by exposure to pathogens, such as bacteria and viruses, as well as toxins, pollutants, irritants, and allergens. During inflammation, numerous inflammatory cells are activated, and each secretes cytokines and mediators to alter the activities of other inflammatory cells. The regulation of these cells and molecules leads to the worsening of inflammation. Asthma and chronic obstructive pulmonary disease (COPD) are serious diseases caused by chronic inflammation [109]. Bronchodilators, corticosteroids, and antibiotics are commonly used in the treatment of acute or chronic lung diseases. However, these do not provide long-term relief in terms of either preventing or treating the disease. Research into understanding the molecular and immunological mechanisms that trigger lung damage could identify promising new therapeutic agents [110].

For this purpose, the pharmacological effects of *M. alba* root bark ethanol extract and its main components on airway inflammation were investigated. In an *in vitro* cell-based assay, KWG suppressed the production of IL-6 cytokine, an important marker of inflammation, in IL-1β-induced inflammatory lung cancer epithelial cells (A549) without causing any cytotoxic effects at the evaluated doses. Furthermore, when evaluating alveolar macrophage models, exposure to stimuli such as lipopolysaccharides (LPS) activates cellular signaling pathways, increasing inducible nitric oxide synthase (iNOS) enzyme expression. Excessive synthesis of nitric oxide (NO), a pro-inflammatory mediator, can contribute to tissue damage during chronic inflammatory processes. In these cell culture tests, KWG reduced NO production by up to 79.9% in alveolar macrophage cells (MH-S) without altering cell viability. These data suggest that KWG presents a potential bioactivity profile relevant to white mulberry root bark’s traditional use, acting as a scaffold for investigating airway inflammatory pathways [61].

In a similar study, KWG exhibited a moderate anti-NO activity on LPS-induced RAW 264.7 macrophage cells (IC_50_ = 17.80 ± 0.50 μM). Except at a high concentration of 100 μM, cell viability remained over 80% in these cell-culture assays. At a dose of 50 μM, KWG reduced iNOS expression compared to the LPS-induced control group and exhibited similar inhibitory activity to the positive control quercetin (25 μM). A molecular docking study suggested that KWG interacts with the active site of the iNOS enzyme with a predicted binding energy of −8.1 kcal/mol, compared to −7.9 kcal/mol modeled for quercetin [60].

In a related study, the anti-inflammatory and anti-allergic potential of KWG was examined in an *in vivo* animal model using ovalbumin (OVA)-induced asthmatic mice. KWG statistically significantly reduced the levels of OVA-specific IgE antibodies in serum and bronchoalveolar lavage (BAL) fluid. Especially at a high dose of 10 mg/kg, it significantly reduced the production of Th2 cytokines (IL-4, IL-5, and IL-13) that worsen the clinical symptoms of asthma. A dramatic decrease in the total number of cells, lymphocytes, neutrophils, and eosinophils (the primary inflammatory cells in asthmatic lungs) was observed in the BAL fluid of mice treated with KWG. These preclinical parameters suggested that KWG suppressed the infiltration of inflammatory cells into lung tissue, the thickening of the bronchiolar epithelium, goblet cell hyperplasia, and collagen deposition. The results of this animal test suggest that KWG modulates pathways associated with the pathological progression of allergic asthma under these experimental conditions [36].

Chang et al. (2019) [44] also investigated the anti-inflammatory properties of 11 bioactive compounds isolated from the root bark of *M. alba*. In *in vitro* cell culture assays, KWG, one of the isolated compounds, inhibited the production of IL-6 by a rate of 98.6% in cells strongly stimulated with TNF-α. Statistically, this *in vitro* effect exhibited a higher percentage of inhibition compared to the efficacy of the BAY 11-7085 molecule (87.9%), which was used as a positive control in the experiment.

In another study, Lee et al. (2014) [37] evaluated the effects of *M. alba* root bark extract and its bioactive compounds on excessive mucin production and secretion in *in vitro* cellular models relevant to serious respiratory diseases such as chronic bronchitis and cystic fibrosis. Mucin is an important defense mechanism that protects the pulmonary system. However, in inflammatory conditions, goblet cells in the surface epithelium overproduce and secrete mucins such as MUC5AC, blocking the airways and worsening these diseases [111]. In these cell culture assays, the administration of KWG to cells stimulated with phorbol 12-myristate 13-acetate (PMA) suppressed this excessive mucin production in a dose-dependent manner. When KWG was added to cells at doses of 1 μM, 10 μM, and 100 μM, mucin production levels decreased to 286%, 204%, and 23%, respectively. It showed high efficacy, markedly reducing induced mucin synthesis in cells, especially at a dose of 100 μM. These findings suggest that this molecule can modulate *de novo* mucin protein production during cellular stimulation, offering a potential bioactivity profile for investigating parameters linked to excessive mucus production [37].

Atopic dermatitis is a common, recurrent, chronic inflammatory disease characterized by skin barrier dysfunction and itching. Keratinocytes are primarily found in the epidermis and play a vital role in host defense by detecting various antigens. These cells produce inflammatory cytokines, chemokines, and adhesion molecules, which play a role in exacerbating cutaneous inflammation. It is known that histamine released by mast cells causes itching, a common symptom of atopic dermatitis [112]. Numerous studies are being conducted to discover substances that can regulate immune balance and exhibit anti-allergic effects in the treatment of atopic dermatitis [113]. Jin et al. (2019) [45] evaluated the mechanisms of KWG *in vitro* using HaCaT keratinocytes and MC/9 mast cells, which are key cells in skin inflammation, and the molecular mechanisms behind these effects. In these cell culture assays, KWG demonstrated no cytotoxicity at concentrations up to 20 μM in HaCaT cells and 10 μM in MC/9 cells. KWG administration reduced the production of pro-inflammatory chemokines, including RANTES/CCL5, TARC/CCL17, and MDC/CCL22, in cells stimulated with TNF-α and IFN-γ. The underlying cellular mechanism was associated with the suppression of STAT1 and NF-κB p65 activation. Furthermore, KWG reduced histamine cellular production, a strong cause of pruritus and allergies, in stimulated MC/9 cells. Additionally, while it suppressed 5-lipoxygenase (5-LO) protein activation, it did not produce any significant change in leukotriene C4 (LTC4) production. These data suggest that KWG exhibits a potential modulatory profile on chemokine and histamine pathways under specific *in vitro* conditions.

Cyclooxygenase (COX) is the key enzyme that acts as a catalyst in the conversion of arachidonic acid, found in cell membranes, into biologically active lipid mediators such as prostaglandins and thromboxanes [114]. This enzyme has two main isoforms, COX-1 and COX-2, which are genetically, functionally, and structurally defined. COX-1 is a housekeeping enzyme responsible for producing prostaglandins, which are essential for normal cellular homeostasis; COX-2, on the other hand, mediates signaling that regulates growth and differentiation or modulates the inflammatory response to infection or injury [115]. The anti-inflammatory properties of Non-Steroidal Anti-Inflammatory Drugs (NSAIDs) are primarily based on their ability to inhibit COX enzymes. This reduces the production of pro-inflammatory prostaglandins at the site of an injury. However, they can also cause serious side effects, such as stomach ulcers and impaired kidney function, due to a decrease in mucosal-protective prostaglandins. Therefore, COX selective inhibitors with narrow side effects are the subject of research for many scientists [114]. In a biochemical screening study designed to evaluate the selective inhibitory effects of kuwanon derivatives obtained from *M. alba* L. roots on COX enzymes, KWG did not show any inhibitory effect on either COX-1 or COX-2 enzymes, even at elevated concentrations reaching 100 µM [68].

Thromboxane A2 (TxA2) is an eicosanoid produced by the sequential action of three enzymes (phospholipase A2, COX-1/COX-2, and thromboxane A2 synthase) in arachidonic acid metabolism. TxA2, named for its role in thrombosis, has prothrombotic properties because it stimulates platelet activation and platelet aggregation. The irreversible inhibition of TxA2 provides protection against vascular thrombotic events [116]. TxA2 is easily converted into thromboxane B, a stable form. Many NSAIDs are known to inhibit the formation of COX products such as HHT, thromboxane B, and prostaglandins, but not lipoxygenase [62]. 12-hydroxy eicosatetraenoic acid (12-HETE) is another important platelet-derived eicosanoid formed by the oxidation of arachidonic acid by the platelet-type 12-lipoxygenase (12-LOX) enzyme [117]. Research has concluded that 12-HETE can exhibit both pro-aggregant and anti-aggregant effects on platelets, depending on its concentration, stereospecificity, and incubation with different agonists [118]. In enzymatic assays investigating these eicosanoid products, KWG reduced the formation of HHT and thromboxane B2 in a dose-dependent manner at concentrations ranging from 10^−5^ to 10^−3^ M. The IC_50_ value of KWG for thromboxane B2 inhibition was calculated as 13.2 × 10^−5^ M. However, KWG stimulated the formation of the lipoxygenase product 12-HETE in a dose-dependent manner. These empirical observations suggest that the 2,4-dihydroxyphenyl and 2,4-dihydroxybenzoyl sub-structures located on the cyclohexene ring of KWG may be associated with modulating thromboxane B2 production under these *in vitro* biochemical conditions [62].

Ulcerative colitis and Crohn’s disease are classified as inflammatory bowel diseases and are characterized by chronic and recurrent inflammation of the digestive system [119]. P-glycoprotein (P-gp), an ATP-binding cassette transporter, effectively regulates xenobiotic accumulation in the gastrointestinal barrier. It thus plays an important role in maintaining normal mucosal homeostasis in the intestine by suppressing excessive immune responses [120]. Jing et al. (2017) [121] investigated whether *M. alba* root bark extract alleviates inflammation and restores microbial balance in an *in vivo* model using rats with induced ulcerative colitis. They also aimed to elucidate at the cellular level how the extract regulates P-gp, an intestinal epithelial barrier protein, either directly or via intestinal bacteria. In an *in vitro* cell culture model evaluating these gastrointestinal barrier parameters, short-term exposure of Caco-2 epithelial cells to 50 μM KWG did not alter the cellular expression of P-gp. However, when cells were exposed to 50 μM KWG for a prolonged duration 7 days, an upregulation of P-gp protein expression was observed. In the pathogenesis of ulcerative colitis, suppression of the P-gp transporter pump in inflamed colon tissue is a key factor that worsens the disease. These findings suggest that long-term KWG application exhibits a potential modulatory profile on P-gp expression under *in vitro* conditions, offering an experimental baseline for investigating intestinal epithelial barrier reinforcement.

Furthermore, KWG has been evaluated for its capacity to modulate the senescence-associated secretory phenotype (SASP). Cellular senescence is a natural process in which cells permanently cease dividing, triggered by various forms of cellular stress, and it helps prevent cancer and aid in tissue repair. These senescent cells release biologically active molecules, including inflammatory cytokines, growth factors, and proteolytic enzymes, known as the SASP. While these molecules are beneficial in acute situations such as wound healing, their persistent accumulation in senescent cells can impair the immune system, trigger chronic inflammation, and contribute to age-related pathologies [122]. When KWG was applied to senescent BJ human skin fibroblasts, it reduced IL-6 production, the primary SASP marker, by more than 15%. Notably, while KWG suppressed IL-6 release, it did not alter the underlying cellular senescence process. These empirical data suggest that KWG does not reverse senescence in these cellular experiments but may limit the inflammatory secretome alterations associated with senescent cells. The results suggest that specific hydroxyl, methoxy, or prenyl substitutions on the phenyl ring may influence this *in vitro* biological activity [123].

**Figure 6:**
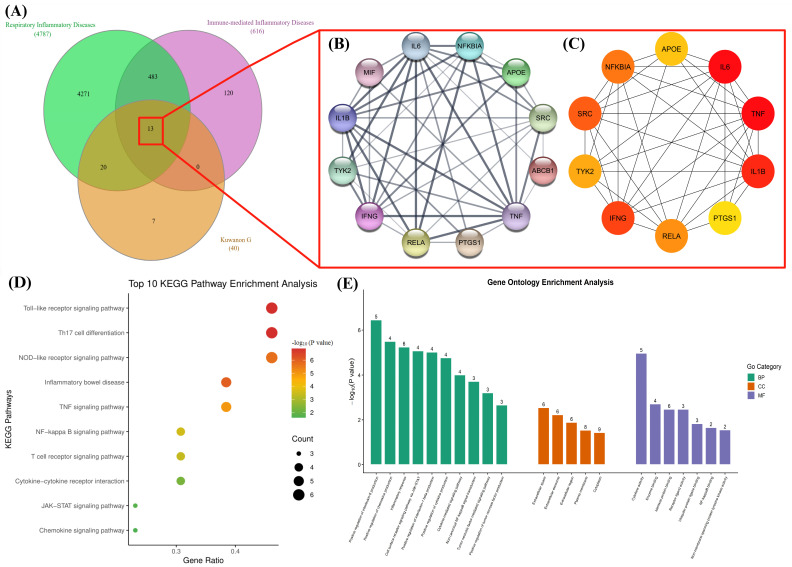
Network pharmacology analysis of Kuwanon G in respiratory inflammatory and immune-mediated inflammatory disease. (**A**) Venn diagram showing the overlap between respiratory inflammatory disease targets (green, *n* = 4787), immune-mediated inflammatory disease targets (pink, *n* = 616) and KWG targets (orange, *n* = 40); 13 common genes were identified. (**B**) The protein–protein interaction (PPI) network was constructed using the STRING database and visualized with Cytoscape. (**C**) Hub genes in the network are colored according to their degree values, ranging from red to yellow. (**D**) KEGG pathway enrichment analysis is shown as a dot plot, where dot size represents the number of genes and the color indicates significance (*p*-value). (**E**) GO enrichment analysis is shown as bar graphs for the categories of biological process (BP), cellular component (CC) and molecular function (MF).

Venn diagram analysis predicted a total of 13 common genes between target genes of respiratory inflammatory diseases (asthma, COPD, and acute/chronic bronchitis), immune-mediated inflammatory diseases (atopic dermatitis, ulcerative colitis, and Crohn’s disease) and KWG targets. The PPI network constructed using these targets shows a high level of connectivity, modeling inflammation-associated molecules such as *IL-6*, *TNF*, *IL-1β*, *NFKBIA*, and *RELA* at the center of the network. This *in silico* projection exhibits a conceptual alignment with the anti-inflammatory effects of KWG reported in the literature, such as suppressing IL-6 production, inhibiting NO synthesis, and regulating the NF-κB signaling pathway. Consistently, prior experimental studies indicate that KWG significantly reduces IL-6 levels and attenuates the inflammatory response by suppressing iNOS expression parallel to these bioinformatic patterns. KEGG pathway enrichment analysis suggests that these common target genes are involved in key biological pathways associated with inflammation and immune response, primarily the TNF, NF-κB, Toll-like receptor, and JAK-STAT signaling pathway. These pathways are characterized in the literature for their central role in chronic inflammatory pathologies. Taken together, these bioinformatic models suggest that KWG offers a potential multi-target profile to regulate pathways involved in inflammation and immune responses (Figure 6).

When experimental findings and network pharmacology analysis are considered together, important clue interpretations can be made regarding the possible molecular mechanisms underlying the anti-inflammatory effects of KWG. The suppression of *IL-6* production by KWG in *IL-1β*-induced A549 cells exhibits a conceptual alignment with the biological importance of *IL-6* and *IL-1β* genes, which are among the central targets in the network analysis. Similarly, the inhibition of NO production in LPS-stimulated MH-S alveolar macrophages and the reduction of iNOS expression in RAW 264.7 cells parallel the inflammation-related signaling pathways identified in the analysis. The significant inhibition of IL-6 production by KWG in TNF-α-induced cells, compared to the positive control, is consistent with the central role modeled for TNF-α and IL-6 in the network analysis. Concurrently, bioinformatic screening predicted genes critical to the inflammatory response, such as *IL-6*, *IL-1β*, and *TNF-α*, which parallels the *IL-6* suppression observed in experimental studies [44,60,61]. In an atopic dermatitis model, studies conducted on HaCaT keratinocytes and MC/9 mast cells showed that KWG significantly reduced the production of RANTES/CCL5, TARC/CCL17, and MDC/CCL22, and that this effect was associated with the suppression of STAT1 and NF-κB p65 (RELA) activation. These prior empirical profiles conceptually match the JAK-STAT and NF-κB signaling pathways that emerged prominently in network pharmacology analysis. Furthermore, the computational identification of *RELA*, *NFKBIA*, and *TYK2* genes as central targets corresponds with these experimentally identified signaling pathways [45].

In conclusion, although KWG exhibits remarkable anti-inflammatory properties across various models like A549, HaCaT, and RAW 264.7 cells, certain translational limitations must be noted. While systemic effectiveness is visible in asthmatic mice, translating these results into actual airway treatments requires addressing tissue-specific delivery and dose optimization. The distinct anti-mucin and anti-allergic effects observed in cell culture highlight KWG as a potential candidate for targeted local or inhalational formulative strategies rather than broad systemic use. Therefore, future research should focus on validating its precise therapeutic windows, local tissue retention, and long-term pulmonary safety profiles to firmly establish KWG as a reliable natural scaffold for chronic inflammatory diseases.

### 3.4. Neuroprotective Effect

Alzheimer’s disease (AD), the most common type of dementia, is a severe, progressive, neurodegenerative disease characterized by memory loss, cognitive decline, and behavioral disorders [124]. Abnormal accumulation of β-amyloid (Aβ) in brain tissues, tau protein aggregation, low acetylcholine levels, increased oxidative stress, and neuroinflammation in the central nervous system are considered the fundamental morphological symptoms of the disease [125]. Acetylcholine (ACh) is a key excitatory neurotransmitter that plays a role in learning, memory, and other higher-level behaviors [124]. Cholinesterase (ChE) is a hydrolase enzyme that breaks down choline esters and has two forms encoded by two different genes: acetylcholinesterase (AChE), which hydrolyzes the neurotransmitter acetylcholine, and butyrylcholinesterase (BChE). AChE terminates ACh-mediated synaptic transmission with high catalytic efficiency by catalyzing the hydrolysis of ACh into choline and acetate [126]. Therefore, cholinesterase inhibitors are one of the main targets of research aimed at investigating potential therapeutic scaffolds for the management of neurodegenerative diseases such as AD.

In a study investigating the *in vitro* AChE, BChE, and BACE1 (β-site amyloid precursor protein cleaving enzyme 1) activities of *M. alba* root bark and its isolated compounds, KWG showed moderate AChE inhibition (IC_50_ = 37.07 μM). However, in these biochemical assays, it exerted significant inhibitory activity against the BChE (IC_50_ = 15.33 µM) and the BACE1 (IC_50_ = 1.01 µM). Furthermore, enzyme kinetics analyses indicated that KWG acts via non-competitive inhibition on AChE and BChE, and mixed-type inhibition on BACE1. Molecular docking studies revealed that KWG has a high binding affinity for AChE of −9.6 kcal/mol and for BACE1 of −11.3 kcal/mol [39].

Ten MDAAs isolated from the root bark of *M. alba* were investigated for their potential regulatory effects on AD-associated targets, as well as their anti-neuroinflammatory and neuroprotective activities *in vitro*. KWG, one of the 10 isolated compounds, showed the highest activity among all tested compounds by inhibiting tau protein aggregation by 96.7% in the thioflavin S (ThS) fluorescence assay. In the antioxidant capacity test, it exhibited 1.9 trolox equivalents, demonstrating a better antioxidant activity potential than the trolox molecule, which was the positive control. However, KWG did not exert a significant preventive effect on Aβ1-42 aggregation (inhibition rate: −3.6%). It was able to inhibit AChE by 42.5% and BChE by only 13.6% (IC_50_ values > 5 μM for both enzymes). It exhibited a weak effect on ChE by inhibiting AChE by 42.5% and BChE by 13.6% (the IC_50_ values for both enzymes are > 5 μM). Furthermore, in a cell-based model using BV2 microglial cells, KWG did not demonstrate sufficient activity to suppress LPS-induced NO production (IC_50_ > 32 μM), and it did not show significant neuroprotective activity against glutamate-induced cell death. Additionally, parallel artificial membrane permeability assays (PAMPA-BBB) determined that KWG has a low ability to cross the blood–brain barrier via passive diffusion [52].

In another study conducted to explore the potential of *M. multicaulis* Perr. branch extract against neurodegenerative markers, the extract’s inhibitory effects on AChE and BChE enzymes were evaluated via the biochemical assays. Both the extract and the reference agent berberine exhibited dose-dependent inhibitory activity on both enzymes. The extract showed higher inhibitory activity against BChE (IC_50_, 101.82 ± 3.37 µg/mL) than against AChE (IC_50_, 179.47 ± 0.38 µg/mL). It has been suggested that this effect may be associated with the presence of KWG, which is found in high concentrations within the chemical profile of the extract [86].

Zhu et al. (2022) [85] used Fe_3_O_4_@BChE magnetic nanoparticles to screen and identify potential BChE inhibitors from mulberry branch extract. KWG was identified as one of the three components bound to the immobilized Fe_3_O_4_@BChE magnetic nanoparticles, indicating a direct interaction with the enzyme. In subsequent enzyme inhibition assays, KWG exhibited inhibitory activity on the BChE with an IC_50_ value of 70.96 ± 2.62 μM. However, the activity of KWG was lower than the BChE inhibition activity of the tested positive control drug galantamine (IC_50_ = 60.35 ± 6.88 μM) and kuwanon H (IC_50_ = 10.91 ± 0.39 μM), the most potent compound in the series.

G-protein-coupled receptors (GPCRs) are a large, diverse class of membrane receptors that convert extracellular signals into intracellular actions, thereby facilitating many physiological functions. Due to their roles in neurotransmission, neuroinflammation, and pathways regulating cell survival, these proteins are of great importance in the field of neurodegenerative diseases [127]. Dopamine receptors (D1R to D5R) are a subclass of GPCRs that play a vital role in regulating motor control and other dopaminergic functions. Dopaminergic neurodegeneration is not the only pathological feature of Parkinson’s disease, but the resulting dopamine deficiency is a major cause of the observed motor impairments. Therefore, GPCRs are potential targets for drug development, and recent estimates suggest that 35% of all FDA-approved drugs target this receptor family [128]. Monoamine oxidase (MAO) is an important enzyme that catalyzes the oxidative deamination of various endogenous and exogenous monoamines. They play important roles in the metabolism of released neurotransmitters and the detoxification of many amines [129]. The MAO enzyme exists in two isoforms: MAO-A and MAO-B. MAO-A primarily deaminates serotonin, norepinephrine, and tyramine, whereas MAO-B deaminates β-phenylethylamine and, to a greater extent, dopamine [130]. Currently, MAO enzymes are considered an important drug target; MAO-A selective inhibitors are seen as potential candidates for depression and anxiety disorders, while MAO-B selective inhibitors are investigated for their therapeutic potential in the context of Parkinson’s and Alzheimer’s diseases [131].

Paudel et al. (2019) [46] aimed to investigate the modulatory effects of DAA compounds (mulberrofuran G, KWG, and albanol B) isolated from the root bark of *M. alba* on human monoamine oxidase (hMAO) enzymes and dopaminergic receptors (D1R, D2LR, D3R, and D4R). When the results regarding KWG were examined, it was found that KWG mildly inhibited the activities of hMAO-A and hMAO-B isoenzymes in biochemical analyses (IC_50_ values of 70.16 ± 2.60 μM and 57.71 ± 2.12 µM, respectively). According to molecular docking studies, KWG exhibited a binding energy of −6.74 kcal/mol in the active site of hMAO-A and bound with a much stronger affinity (−12.65 kcal/mol) to the active site of hMAO-B. Furthermore, in *in vitro* cellular functional assays, KWG acted as a complete antagonist on D1R/D2LR while demonstrating full agonist activity on D3R/D4R. Specifically, KWG suppressed the dopamine-induced response on D1R and D2LR by 98.85 and 99.15%, respectively. while its independent agonist effects on D3R and D4R were 124.3% and 90.45%, respectively. Molecular docking simulations with these dopamine receptors suggested binding energies of −7.1 kcal/mol (D1R), −8.23 kcal/mol (D2LR) −7.45 kcal/mol (D3R), and −10.34 kcal/mol (D4R). In summary, this study suggested that KWG displays an *in vitro* profile consistent with a D1R/D2LR antagonist and D3R/D4R agonist, while showing mild hMAO inhibition across these screening platforms.

Diabetic encephalopathy refers to cognitive impairment and the neurophysiological and structural changes in the brain caused by diabetes [132]. Advanced glycation end products (AGEs), formed because of non-enzymatic glycation and oxidation of proteins and reducing sugars, are known to initiate and exacerbate pathological damage in diabetic encephalopathy. AGEs activate the advanced glycation end-product receptor and mediate the ROS-dependent activation of the transcription factor nuclear factor kappa B (NF-κB) and other regulatory cytokines. The accumulation of AGEs can damage brain cells by triggering oxidative stress and inflammatory responses [133]. To evaluate these mechanisms, Gan et al. (2021) [134] utilized an *in vitro* cell-based model where HT22 hippocampal neurons were exposed to AGEs. While AGE exposure led to dose-dependent cell death, KWG administration was found to increase cell viability in this model. At the molecular level, KWG downregulated apoptotic markers by reducing Bax and increasing Bcl-2 expressions. Furthermore, KWG attenuated the cellular ROS accumulation and MDA increase caused by AGEs, while also mitigating neurotoxicity by enhancing the cell’s primary antioxidant defense mechanisms (SOD and GPX). In the same cell culture model, KWG treatment increased intracellular and extracellular ACh levels, correlating with the stimulation of ChAT enzyme production and the suppression of AChE enzyme secretion. Additionally, KWG increased the phosphorylation of PI3K/Akt; conversely, it modulated the activation of the GSK3α, GSK3β, p38 MAPK, and NF-κB p65 pathways. In summary, these *in vitro* models highlight KWG as a pharmacological scaffold for investigating neuroprotective mechanisms relevant to diabetic encephalopathy due to its anti-apoptotic, antioxidant, and acetylcholine-preserving properties.

Zhang et al. (2025) [135] aimed to elucidate the multiple molecular mechanisms, potential regulatory targets, and signaling pathways underlying the potential effects of KWG associated with the management of diabetic encephalopathy using *in silico* bioinformatics and network pharmacology approaches. As a result of database searches, a total of 1.058 disease-specific target genes were identified for diabetic encephalopathy, while 101 potential target genes with which KWG could interact were identified. Cross-sectional analyses predicted that there are 34 common core target genes that may be involved in modulating pathways relevant to diabetic encephalopathy. PPI network analysis revealed that KWG targets the top 10 genes (*ALB*, *CASP3*, *PPARG*, *HSP90AA1*, *ESR1*, *GSK3B*, *ANXA5*, *PARP1*, *IGF1R*, and *MAPK14*) associated with diabetic encephalopathy. GO analyses determined that these common targets strongly regulate a total of 58 cellular biological processes, primarily including RNA polymerase II-specific DNA-binding transcription factor activity, carboxylic acid binding, endopeptidase activity, and MAP kinase activity. Through KEGG pathway analysis, key putative pathways that may mediate the modulatory effects of KWG in the context of diabetic encephalopathy were highlighted. These were determined as the lipid and atherosclerosis pathway, the IL-17 signaling pathway, the AGE-RAGE (Advanced Glycation End-product Receptor) signaling pathway, the insulin resistance pathway, the TNF signaling pathway, and the PI3K-Akt signaling pathway.

Another study by Zhang et al. (2025) [136] attempted to identify the bioactive metabolites of KWG formed by gut flora and intrabody processes and to determine the multi-target interactions of these metabolites in pathways associated with diabetic encephalopathy through network pharmacology and molecular simulations. Using *in vitro* liver microsome and gut microbiota incubation assays analyzed via UPLC-MS, a total of 56 KWG metabolites were identified. The capacity of the gut microbiota to convert KWG (37 metabolites) was found to be higher than that of liver microsomes (24 metabolites). In addition, the study indicated that KWG is not detected in its original form in the systemic circulation (blood) suggesting that it mainly enters the bloodstream as microbiota-derived metabolites. To assess central nervous system parameters, Swiss ADME screening has highlighted four metabolites with the potential to cross the blood–brain barrier. Network pharmacology identified AKT1, TNF, SRC, EGFR, and ESR1 as the top five target proteins for these metabolites in the context of diabetic encephalopathy. Furthermore, it suggested that the potential mechanism involves the attenuation of neuroinflammation and apoptosis via the PI3K-Akt signaling pathway. Molecular docking and simulation studies also showed that the original structure of KWG displays a low binding affinity to these specific receptors, whereas the active metabolites are predicted to exhibit a strong binding affinity to the same targets.

You and Kim (2019) [63] investigated the neuroprotective effects of ethanol extract obtained from the root bark of *M. alba* (MCR) in a cell-based *in vitro* model using PC12 neuronal cells against oxidative stress caused by high glucose (HG). While HG exposure increased intracellular ROS levels by 43%, MCR inhibited this increase in a dose-dependent manner. At the same time, it reduced the high MDA (lipid peroxidation) levels caused by high glucose in a dose-dependent manner, bringing them down to the levels seen in the healthy control group. Cellular SOD and GSH enzyme activities, which were suppressed by HG stress and reduced by 15% to 50%, were increased again with MCR treatment of 50 µg/mL and above. At the molecular level, MCR upregulated the cell survival protein p-Akt, balanced the Bax/Bcl-2 ratio to favor survival, and inhibited the overexpression of cytochrome c. HPLC analyses revealed that KWG and morusin were highly isolated compounds in the MCR structure. In the cell culture assay, when administered to PC12 cells at concentrations of 0.1 and 1 μM, KWG attenuated HG-induced cell death, and cell viability was determined to be 72.5% and 77.6%, respectively. Based on these results, the authors hypothesize that the antioxidant activity and neuroprotective effect of MCR in the presence of HG may be due to KWG and morusin, as well as the phenolic compounds of MCR.

Venn diagram analysis revealed 33 computationally predicted common genes between neurodegenerative and metabolic brain diseases and KWG targets. The PPI network constructed using these common genes shows a dense network of interactions between the target proteins. Network analysis predicts that genes critical to inflammation, cellular stress response, and signal transduction, such as *IL-6*, *TNF*, *IL-1β*, *RELA*, *NFKBIA*, *SRC*, and *HSP90AA1*, occupy central positions. Furthermore, the presence of enzymes associated with neurodegenerative processes, such as AChE, BChE, and BACE1, suggests that KWG may interact at the network level with elements of the cholinergic system and amyloid metabolism associated with Alzheimer’s disease.

**Figure 7:**
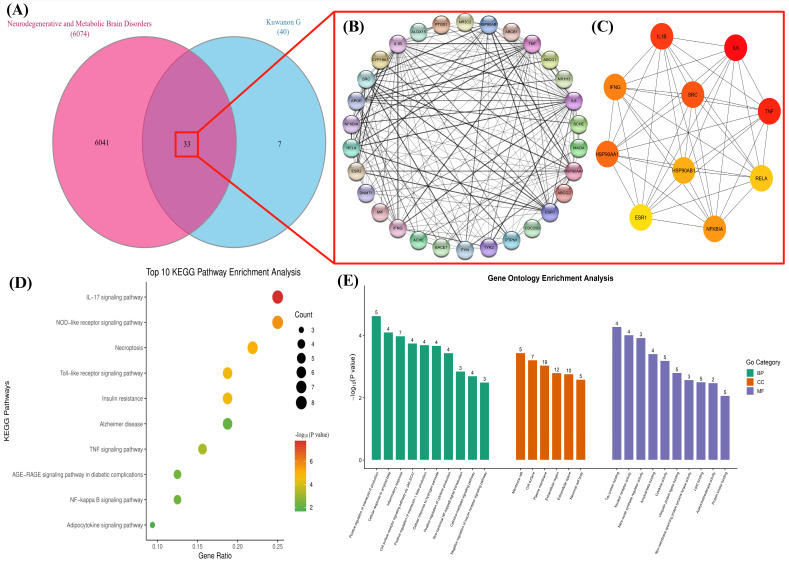
Network pharmacology analysis of Kuwanon G in neurodegenerative and metabolic brain disorder. (**A**) Venn diagram showing the overlap between neurodegenerative and metabolic brain disorder targets (pink, *n* = 6074) and KWG targets (blue, *n* = 40); 33 common genes were identified. (**B**) The protein–protein interaction (PPI) network was constructed using the STRING database and visualized with Cytoscape. (**C**) Hub genes in the network are colored according to their degree values, ranging from red to yellow. (**D**) KEGG pathway enrichment analysis is shown as a dot plot, where dot size represents the number of genes and the color indicates significance (*p*-value). (**E**) GO enrichment analysis is shown as bar graphs for the categories of biological process (BP), cellular component (CC) and molecular function (MF).

To distinguish these *in silico* predictions from experimentally validated mechanisms, these findings were evaluated in conjunction with *in vitro* literature. KWG’s notable experimentally validated *in vitro* inhibitory activity against BChE and BACE1 and its observed effects on ACh levels in cell models, are consistent with the neurodegenerative targets computationally identified in the network analysis. In the KEGG enrichment analysis, putative inflammatory pathways, as well as mechanisms associated with insulin resistance and Alzheimer’s disease, were prominently enriched. These findings computationally match KWG to numerous targets associated with neurodegenerative and metabolic brain diseases (Figure 7).

When *in vitro* experimental studies and network analysis results are critically compared, it is suggested that the modulatory profile of KWG against AD-related targets involves a multi-target mechanism. Previous *in vitro* studies have empirically validated KWG’s inhibitory activity against BACE1, successfully corroborating its identification as a computationally predicted target in the network analysis. In contrast, while experimental studies indicate that KWG does not exhibit significant *in vitro* inhibition of Aβ1-42 aggregation, targets directly related to amyloid aggregation are not among the common targets in the network analysis [39,52]. In this study, it was reported that KWG displayed mild *in vitro* inhibitory activity against both hMAO-A and hMAO-B. The MAO-A gene was independently predicted among the common targets in the network analysis [46]. Furthermore, the experimentally observed findings reported by Gan et al. (2021) [134] closely align with some targets and signaling pathways predicted in the network analysis. The NF-κB-modulating effects observed in the *in vitro* cellular model correspond to the *RELA* and *NFKBIA* genes, which are among the computationally mapped targets in the network analysis. Similarly, the pathways modulated in the study in the context of AGE-induced neurotoxicity are consistent with the AGE-RAGE signaling pathway, which is enriched in the KEGG analysis. The findings regarding acetylcholine metabolism reported in the study are associated with the ACHE gene, which is among the common targets in the network analysis, while the Alzheimer’s disease pathways identified in the KEGG analysis also suggest that these modulatory mechanisms are reflected at the network level.

Although these correlations between specific *in vitro* assays and *in silico* targets, a critical distinction must be made regarding the broader network findings. While network pharmacology provides a robust predictive framework, the comprehensive pathway enrichments (such as the AGE-RAGE or systemic inflammatory signaling pathways) remain computationally theoretical at this stage. Rigorous *in vivo* and clinical validations are strictly required to confirm whether KWG can effectively modulate these entire predicted networks in a complex physiological environment.

From a broader translational perspective, it is crucial to emphasize that despite the promising *in vitro* and *in silico* findings regarding the neuroprotective modulatory profile of KWG, its translational potential is currently hindered by significant pharmacokinetic limitations. As previously noted, the compound exhibits poor blood–brain barrier penetration via passive diffusion and is extensively metabolized by gut microbiota, meaning the parent compound rarely reaches the central nervous system in its original form. Therefore, extrapolating these target-level findings to clinical efficacy is premature. Future *in vivo* studies focusing comprehensively on its systemic bioavailability, the distinct pharmacokinetics of its active metabolites, and its long-term toxicity profile are essential to accurately define the true therapeutic boundaries of KWG.

### 3.5. Anti-Obesity Activity

Obesity is a chronic, complex, and multifactorial disease characterized by excessive fat accumulation in the body and significantly impairing health. Obesity disrupts the body’s normal metabolic processes and therefore increases the risk of developing diseases such as type 2 diabetes, hypertension, fatty liver disease, myocardial infarction, heart failure, stroke, obstructive sleep apnea, osteoarthritis, mental disorders, and certain types of cancer [137]. According to a report published by the World Obesity Federation in 2024, projections for 2035 indicate that more than 1.77 billion people will be overweight and 1.53 billion will be obese, representing 54% of all adults worldwide [138].

Inhibition of the pancreatic lipase (PL) enzyme is a proven strategy for combating obesity. PL is an important enzyme in the digestive system that plays a role in the breakdown of lipids into free fatty acids and monoglycerides. The activity of this enzyme is crucial for lipid absorption and metabolism, making it an attractive target for obesity intervention. Inhibition of PL is therefore an effective strategy for weight reduction by interfering with the body’s capacity for storing lipids [139]. In a study investigating new-generation natural PL inhibitors for the treatment of obesity, the main components of mulberry root bark were examined for this purpose. In an *in vitro* enzymatic assay, the inhibitory potentials of six main natural compounds (sanggenon C, sanggenon D, kuwanon C, KWG, morin, and morusin) were investigated using 4-methylumbelliferyl oleate (4-MU oleate) as probe substrate. Among all the components tested, sanggenone C, sanggenone D, kuwanon C, and KWG presented notable inhibitory profiles against PL. Among these four components, the highest IC_50_ value was calculated for KWG (4.85 ± 0.87 µM). However, this value is lower than the IC_50_ value of the positive control kaempferol (15.30 µM). Enzyme kinetic analyses demonstrated that KWG acts as a reversible, mixed-type inhibitor of PL-mediated 4-MU oleate hydrolysis, presenting a calculated *K*_i_ value of 3.50 μM [140].

The endocannabinoid system (ECS) is a complex system involved in various physiological and pathological processes in mammals, and it is known to regulate various metabolic processes such as food intake and energy expenditure. The receptors and endogenous ligands are the most important parts of the ECS. These receptors are called cannabinoid receptors; the most well-known are type 1 (CB1) and type 2 (CB2) [141]. CB1 is widely distributed throughout the central nervous system; its expression is primarily observed in the hippocampus, ganglia, cerebral cortex, cerebellum, limbic system, and hypothalamus. CB2 is primarily expressed in spleen, thymus, and circulating immune cells, as well as in the skeletal, cardiovascular, and renal systems. ECS receptors are also expressed in adipose tissue, where they exert a direct influence on lipid metabolism [142]. For this purpose, Yimam et al. (2019) [64] screened plant extracts for cannabinoid receptor type 1 (CB1) antagonists, identifying *M. alba* as a leading candidate. In an *in vitro* binding assay focusing on individual components isolated from the root bark, the IC_50_ values of KWG for the CB1 and CB2 receptors were determined to be 10.1 μM and 28.9 μM, respectively. In parallel enzymatic screenings, KWG did not have inhibitory activity on the α-glucosidase enzyme (−22%). These preclinical data suggest that *M. alba* root bark extract enriched with KWG presents a potential bioactivity profile that warrants further investigation regarding calorie intake regulation and metabolic syndrome management parameters.

**Figure 8:**
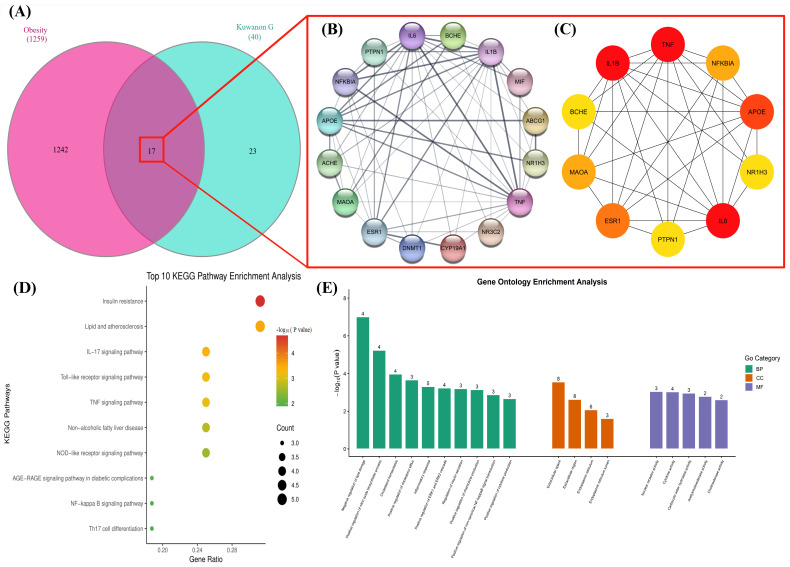
Network pharmacology analysis of Kuwanon G in obesity. (**A**) Venn diagram showing the overlap between obesity targets (pink, *n* = 1259) and KWG targets (blue, *n* = 40); 17 common genes were identified. (**B**) The protein–protein interaction (PPI) network was constructed using the STRING database and visualized with Cytoscape. (**C**) Hub genes in the network are colored according to their degree values, ranging from red to yellow. (**D**) KEGG pathway enrichment analysis is shown as a dot plot, where dot size represents the number of genes and the color indicates significance (*p*-value). (**E**) GO enrichment analysis is shown as bar graphs for the categories of biological process (BP), cellular component (CC) and molecular function (MF).

A comparison of obesity-related target genes with KWG targets predicted 17 common genes between the two groups. The PPI network constructed using these common genes shows a dense network of interactions between the target proteins. Analysis of the network topology suggests a theoretical model where genes associated with inflammation, lipid metabolism, and hormonal regulation, such as *IL-6*, *TNF*, *IL-1β*, *NFKBIA*, *APOE*, and *ESR1*, occupy central hub positions. Concurrently, KEGG pathway enrichment analysis indicated that these genes are primarily concentrated in biological pathways traditionally linked to metabolic disorders, specifically insulin resistance, lipid and atherosclerosis, IL-17, Toll-like receptor, TNF, and NF-κB signaling pathways. These pathways encompass inflammatory and metabolic processes that are modeled to contribute to the development and complications of obesity. In the GO enrichment analysis, the computational targets were found to be concentrated in processes such as negative regulation of lipid storage, cholesterol homeostasis, inflammatory response, regulation of insulin secretion, and positive regulation of chemokine production. These network pharmacology patterns suggest that KWG has theoretical capacity to influence key mechanisms involved in the pathogenesis of obesity (Figure 8).

However, the molecular target profiles and key signaling pathways identified through our network pharmacology approach represent computational models that require empirical validation. While these *in silico* tools provide a comprehensive framework for hypothesis generation, they cannot replace direct experimental evidence. The predicted targets and signaling pathways identified through network analysis have not yet been fully mapped in the literature; therefore, they must be validated through further *in vitro*, *in vivo* models, and clinical studies. These computational findings provide targeted guidance for research and are expected to steer future mechanistic studies aimed at exploring the therapeutic potential of KWG.

Despite the promising *in vitro* enzymatic profiles of KWG, its translational potential in obesity management is currently limited by crucial pharmacokinetic and safety parameters. Pancreatic lipase inhibition occurs directly within the gastrointestinal lumen, meaning KWG must maintain dynamic structural stability against digestive enzymes and local pH changes to remain active. Furthermore, evaluating KWG’s efficacy against adipocyte targets, such as the CB1 and CB2 receptors in adipose tissue, requires effective systemic absorption and tissue-specific distribution. Currently, comprehensive data on the systemic bioavailability, distribution characteristics in peripheral tissues, and chronic toxicity profiles of KWG are still lacking. Therefore, rigorous *in vivo* studies focusing on absorption kinetics and long-term toxicity are strictly required before translating these computational and enzymatic findings into validated clinical strategies for obesity intervention.

### 3.6. Antimicrobial Activity

#### 3.6.1. Antibacterial Activity

The oral cavity harbors a diverse microbiome consisting of hundreds of bacterial species that coexist in a delicate balance. However, opportunistic pathogens in the mouth can easily disrupt this balance and cause common oral diseases, such as tooth decay and periodontal disease. The most important oral pathogens include the herpes virus, *Candida albicans*, *Streptococcus mutans*, *Porphyromonas gingivalis*, *Fusobacterium nucleatum*, *Actinobacillus actinomycetemcomitans*, *Prevotella intermedia*, *Treponema denticola*, and *Tannerella forsythia*. Oral infections, beyond their local effects, can lead to very serious systemic health problems such as infective endocarditis, diabetes, Alzheimer’s disease, chronic lung disease, rheumatoid arthritis, and cardiovascular disorders [143,144]. Traditional methods used to treat oral infections, such as chlorhexidine mouthwashes and antibiotics, disrupt the balance of the oral microbiome because they affect the entire bacterial community indiscriminately. They can also cause unwanted side effects such as vomiting, diarrhea, drug resistance, and tooth discoloration. For this reason, there is a need to develop alternative antimicrobial agents with few or no side effects [145,146]. Park et al. (2003) [35] demonstrated the antibacterial effects of KWG on oral pathogens that cause tooth decay *in vitro* screening assays. KWG had a much stronger effect on *S. mutans*, with minimum inhibitory concentration (MIC) value of 8 µg/mL, compared to sanguinarine (32 µg/mL), carvacrol (125 µg/mL), green tea extract (250 µg/mL), thymol, and eucalyptol (both 500 µg/mL). In addition, the compound has demonstrated the same level of efficacy against *P. gingivalis*, *Streptococcus sobrinus* and *Streptococcus sanguis* (MIC = 8 µg/mL). In contrast, no antimicrobial activity was observed against *C. albicans*, *A. actinomycetemcomitans*, *Lactobacillus acidophilus*, and *Lactobacillus casei*. In time-kill tests, *S. mutans* cells were treated with 5, 10 and 20 µg/mL KWG for 1–10 min. 20 µg/mL KWG was found to completely inactivate and kill *S. mutans* cells within just one minute by destroying its cellular integrity. These preliminary findings suggest that the compound represents a potential candidate for topical oral care formulations. The authors suggest that the hydroxyl groups in the aromatic ring of KWG may be responsible for its antibacterial activity.

Tuberculosis is a serious disease caused by an infection with the bacterium *Mycobacterium tuberculosis* that primarily affects the respiratory tract [147]. The 2024 Global Tuberculosis Report estimates that there were 10.8 million cases of tuberculosis worldwide, 1.25 million of which resulted in death. The emergence of drug-resistant strains is making efforts to eliminate the disease even more difficult [148]. Antivirulence strategies represent a promising area of research in the development of novel, more effective tuberculosis treatments and in combating antibiotic resistance [149]. The phosphatases PtpA and PtpB, which are secreted by *M. tuberculosis*, are key virulence factors that play a crucial role in the survival of the bacteria during infection of macrophages [150]. The primary function of PtpB is to ensure the pathogen’s survival within the cell by suppressing the host’s innate immune response. The enzyme disrupts host cell signaling pathways, including JNK, p38, and NF-κB, and engages with proteins such as TRIM27 and ubiquitin to render the immune system. The rise in cases of multidrug-resistant and extensively drug-resistant tuberculosis has positioned PtpB as a strategic molecular target for the development of new drugs. The inhibitory effects of natural DAA isolated from the root bark of *M. nigra* on PtpB were investigated. In target-specific biochemical assays, KWG inhibits the PtpB enzyme with an IC_50_ value of 0.83 ± 0.35 µM, presenting a calculated *K*_i_ of 0.39 ± 0.27 µM. Compared to other phosphatases, KWG showed a 7.1-fold selectivity for PtpB over PtpA, and a 5.5-fold selectivity over the human PTP1B enzyme. Kinetic studies indicate that KWG binds directly to the active site of PtpB and competes with the substrate. Isothermal titration calorimetry (ITC) measurements reveal that the binding is exothermic and occurs primarily via hydrogen bonds. Molecular modeling studies show that KWG forms hydrogen bonds with critical amino acids in the enzyme’s active site, such as Cys160, Tyr125, Asp165, and Arg166. Additionally, interestingly, KWG inhibits the human PTP1B enzyme in a “non-competitive” manner, that is, by binding to an allosteric site outside the active site. The MIC value of KWG against *M. tuberculosis* (Mtb) H37Ra strain was determined to be 32 μg/mL using the resazurin microtiter assay. The MTT reduction assay revealed that the cytotoxic dose of KWG for macrophages (the cytotoxic concentration that kills 50% of the cells (CC)_50_ = 33.77 μg/mL) is nearly identical to this MIC parameter. Therefore, when evaluated at a non-toxic concentration of 10 μg/mL within cell culture models, KWG restricted intracellular *M. tuberculosis* growth by 61.3%, indicating a baseline antivirulence activity that warrants further investigation. Consequently, the authors state that KWG may serve as a potential candidate for future drug discovery efforts targeting tuberculosis virulence mechanisms [80].

The development of antibiotic resistance by bacteria causes hundreds of thousands of deaths every year [151]. The WHO recently identified antimicrobial resistance as one of the top 10 global public health threats. It is predicted that antibiotic-resistant infections will cause 10 million deaths annually by 2050 [152]. Methicillin-resistant *Staphylococcus aureus* (MRSA), one of the most problematic antibiotic-resistant bacteria, is a leading cause of the spread of hospital- and community-acquired infections that lead to serious and life-threatening illnesses [153]. Phytochemicals are among the alternative treatments currently being used or in the trial phase to treat infections caused by drug-resistant bacteria [154]. For this purpose, the activity of KWG against MRSA was also evaluated. Horhogea et al. (2017) [155] investigated the antibacterial activity of KWG against MRSA strains and its interactions with four antibiotics (oxacillin (OX), amoxicillin (Amx), erythromycin (Er), and gentamicin (Gn)). In these *in vitro* assays, KWG demonstrated antibacterial activity against four different clinical MRSA strains (T1–T4) selected for the study, with MIC values ranging from 6.25 to 12.5 µg/mL. In the synergy test, a synergistic effect (FICI ≤ 0.5) was observed against most of the MRSA strains tested in combination with all antibiotics. Only the combination of KWG with Amx showed an additive effect on the T4 strain. Time-kill assays confirmed the synergistic bactericidal effect of the KWG-Amx (for T1 and T2 isolates) and KWG-Gn (for all isolates) combinations. These preclinical data suggest that effective combinations of KWG and antibiotics can be developed for the treatment of gram (+) bacteria including MRSA.

In a different study by the same researchers, the synergistic interactions of morusin and KWG obtained from *Morus* spp. with antibiotics (tetracycline (TE) and ciprofloxacin (CIP)) against MRSA strains were investigated against MRSA strains. KWG alone demonstrated a good activity against all MRSA strains, with MIC values ranging from 6.25 to 12.50 µg/mL. The combination of TE and KWG showed synergistic effects against *S. aureus* ATCC 33591 and MRSA T3 clinic strains, and additive effects against other strains. Specifically, the combination reversed TE resistance in *S. aureus* ATCC 33591 and MRSA T4 clinic strains, reducing the MIC value to below the Clinical & Laboratory Standards Institute (CLSI) susceptibility limit of 4 µg/mL. When combined with CIP, a synergistic effect was noted in *S. aureus* ATCC 33591, MRSA T1, and MRSA T2 strains, whereas an additive effect was observed in the other strains. KWG also increased the susceptibility of bacteria to CIP, resulting in a 2- to 16-fold reduction in antibiotic MIC values. According to the time-dependent killing assay, the combination of KWG and CIP exhibited bactericidal synergy against *S. aureus* ATCC 33591, demonstrating a reduction in bacterial count of 2.63 ± 0.21 log_10_ CFU/mL within 24 h [156].

Another study by Aelenei et al. (2020) [157] evaluated the interactions between KWG and conventional antibiotics against *S. aureus* ATCC 43300 (MRSA) and *S. epidermidis* ATCC 12228. In these *in vitro* assays, KWG demonstrated antimicrobial activity against MRSA with an MIC value of 12.50 μg/mL. Membrane permeability evaluations showed that approximately 40% of MRSA cells exposed to KWG for 15 min, and over 90% of those exposed for 60 min, sustained cell membrane damage, leading to the leakage of cellular contents. According to the FICI analysis, KWG demonstrated synergistic activity against MRSA when combined with OX, Gn, CIP and TE, reducing the antibiotic MIC values by 64-, 8-, 32- and 4-fold, respectively. In other words, this combination altered OX resistance profile of the strain. A synergistic effect was observed in combinations of KWG with OX and CIP antibiotics against *S. aureus* ATCC 6538 (MSSA) and with Gn and CIP against *S. epidermidis* ATCC 12228. However, while these KWG combinations demonstrated synergy in checkerboard tests, they did not produce bactericidal synergy in parallel time-kill assays.

Similarly, Zhu et al. (2021) [55] investigated the activity of the most active antibacterial fraction (MA-6 fraction) obtained by ethanol extraction of *M. alba* root bark and the components isolated from it against MRSA. In standard broth microdilution tests, KWG demonstrated antimicrobial activity against clinical MRSA strains (MRSA-031, MRSA-011, MRSA-003) with an MIC value of 4 μg/mL. The MIC value against the standard *S. aureus* ATCC 43300 strain was recorded as 2 μg/mL. The minimum bactericidal concentration (MBC) values obtained against all bacteria ranged between 4 and 8 μg/mL. Compared to the control agents ampicillin and berberine, KWG exhibited higher antibacterial activity. Structural analyses determined that the prenyl moiety within KWG is associated with its anti-MRSA activity. The authors scientifically explained that prenyl units increase the lipophilicity of the compound, which may facilitate cell membrane penetration and subsequent target binding.

Another study investigated the antibacterial activities of flavonoids derived from *M. alba* and specifically examined the potential mechanisms of action of KWG against MRSA. When the study was evaluated in terms of KWG, it was determined that KWG demonstrated antibacterial activity against *S. aureus* ATCC 29213 (MSSA) and MRSA T144 strains with a MIC value of 2 µg/mL, and against *Bacillus subtilis* and *Enterococcus faecalis* strains with a MIC value of 4 µg/mL. The MIC_90_ and MIC_50_ values against 20 MRSA strains were 8 and 4 μg/mL, respectively. According to the time-kill test, a KWG concentration of 8 μg/mL reduced the viable cell counts of both MRSA T144 and MSSA ATCC 29213 within the first hour of treatment. Comparative assays indicated that while the cell-wall-targeted antibiotic vancomycin (8 μg/mL) required 12 h to eliminate the bacteria, KWG and the reference membrane-active drug daptomycin (8 μg/mL) eradicated both strains within 3 h. Because KWG exhibits bactericidal kinetics similar to daptomycin, the authors suggest that this metabolite primarily targets the bacterial cell membrane. Subsequently, the effects of KWG on the bacterial proton motive force (PMF) were evaluated. In fluorometric analyses utilizing the membrane potential-sensitive probe DiSC3(5), KWG administration induced a dissipation of both PMF components, namely the transmembrane electrical potential (Δψ) and the transmembrane proton gradient (ΔpH). Under experimental conditions where the external pH was adjusted to 9.5, a 64-fold increase in MBC value of KWG was observed, indicating that its antimicrobial action is highly dependent on ΔpH disruption. To assess barrier function alterations, *in vitro* analyses utilized the propidium iodide (PI) probe. The rapid internalization of PI following KWG application indicated macromolecular pore formation and rapid membrane integrity disruption. This structural dysfunction was morphologically confirmed via laser-scanning confocal microscopy (SYTO 9/PI double staining) and TEM imaging.

According to data from molecular docking studies, the molecular basis of this damage is that KWG destabilizes the lipid bilayer by forming high-affinity hydrogen bonds and electrostatic interactions with phosphatidylglycerol (PG) and cardiolipin (CL) molecules, which serve as building blocks in the bacterial membrane. In luminescence-based ATP quantification assays, KWG-treated cells demonstrated a depletion of intracellular ATP accompanied by a simultaneous increase in extracellular ATP concentrations. Fluorometric cell-based tests indicated intracellular ROS production does not deviate significantly from baseline levels under standard bactericidal concentrations (1–8 μg/mL). However, a higher concentration of 16 μg/mL triggered oxidative stress, which contributed to the structural dysfunction of the cytoplasmic membrane. To measure potential resistance development profiles, bacteria were exposed to sub-lethal doses (0.5 × MIC) of these agents for 30 consecutive days. By the end of this period, resistance to oxacillin increased 128-fold and to enrofloxacin 32-fold, while resistance to KWG increased only 4-fold. In safety screenings using mammalian cell lines, the IC_50_ values of KWG on HepG2 and Vero cells were found to be 114.8 and 92.2 μg/mL, respectively, while the half-hemolytic dose (H*ly*_50_) in sheep red blood cells was 19.8 μg/mL. Based on these cytotoxicity and hemolysis results, the authors suggested that KWG may not be suitable for systemic administration. Finally, to assess the effect of KWG on living organisms, an *in vivo* mouse wound healing model was established. To avoid systemic toxicity, 40 μg of KWG was applied topically to the wounds. By day 14, the bacterial load in the KWG-treated group decreased compared to the control (DMSO), promoting wound healing. However, because KWG also binds to mammalian cells, its efficacy was lower than that of the reference antibiotic vancomycin. These preclinical findings suggest that KWG represents a potential scaffold for designing topical membrane-active antimicrobials against MRSA infections [43].

The two-component systems (TCSs) are found in most bacteria and serve as the primary signal transduction pathways that bacteria use to regulate various processes, including growth, metabolism, virulence, and antibiotic resistance. TCSs typically consist of a histidine kinase (HK), a homodimeric sensor enzyme usually bound to a substrate, and a response regulator (RR) that acts as the corresponding effector [158]. HK is bifunctional, possessing both kinase activity (acting on histidine) and phosphatase activity (acting on phosphoaspartate). RRs are targets of histidine kinase phosphatases [159]. In response to environmental or metabolic signals, HKs undergo autophosphorylation at conserved histidine residues. The phosphate groups on the histidine residues of the HKs are subsequently transferred to the conserved aspartate residues in the receptor sites of the corresponding RRs. Phosphorylation of the conserved regulatory domain of RR leads to a change in its ability to bind to DNA and influence transcription [160]. Since TCSs play a key role in bacterial signal transduction, inhibitors of these proteins may have the potential to exhibit broad-spectrum antibacterial activity, making them attractive targets. TCSs can generally be used to overcome resistance to many commonly used antibiotics, such as β-lactams and vancomycin, as well as last-resort antimicrobials like colistin [158]. Based on the observation that histidine kinases also possess phosphatase activity, Barker et al. (2020) aimed to overcome colistin resistance by disrupting bacterial signaling pathways using eukaryotic phosphatase inhibitors. In this study, which screened 48 different eukaryotic phosphatase inhibitors, KWG emerged as one of the most potent compounds identified. When combined with 20 μM KWG, colistin resistance decreased in resistant strains of *Acinetobacter baumannii* and *Klebsiella pneumoniae*. Specifically, KWG reduced the colistin MIC value in the *A. baumannii* AB4106 strain from 1024 μg/mL to 0.5 μg/mL, reflecting in a 2048-fold decrease. A 512-fold decrease was also observed in *A. baumannii strains* AB3941 and AB3942, with the concentration dropping from 512 μg/mL to 1 μg/mL. In the *K. pneumoniae* KPB9 strain, it reduced the concentration from 1024 μg/mL to 1 μg/mL, resulting in a 1024-fold decrease. These combinational test parameters restored colistin susceptibility below the CLSI safety threshold (≤2 μg/mL). 20 μM of KWG reduced the colistin MIC value in the *A. baumannii* ATCC 17978*^mcr^*^−1^ strain, which carries the mcr-1 gene, from 16 μg/mL to 0.5 μg/mL (32-fold); in the *E. coli* ATCC 25922*^mcr^*^−1^ strain, it reduced the MIC value from 8 μg/mL to 1 μg/mL (8-fold). When applied independently, KWG did not exhibit direct antimicrobial effect or alter membrane permeability significantly. Based on these findings, the authors suggest that the molecule’s primary mechanism against gram (−) strains does not involve structural membrane perforation. In parallel *in vitro* assays, when administered in combination with polymyxin B nonapeptide (PMBN)—a non-bactericidal outer membrane permeabilizer—KWG did not exhibit independent antibacterial activity. This result demonstrated that KWG functions specifically as a resistance-modifying adjuvant to enable colistin activity. In cell viability screenings using an MTT assay on 4T1 mouse mammary tumor cells, the cytotoxic dose of KWG required to kill 50% of the cells (CT_50_) was found to be 114.1 μM. When colistin was added at a therapeutic dose of 1 μg/mL, no increase in the compound’s toxicity was observed (CT_50_, 101.3 μM). Interestingly, qPCR analyses revealed that KWG does not reduce *pmrCAB* expression. This evaluation suggests that KWG modulates gram (−) resistance through an unelucidated signaling pathway distinct from these known genetic cascades [161].

*K. pneumoniae* is a gram (−) opportunistic pathogen that causes various community-acquired and hospital-acquired infections, such as urinary tract infections, pneumonia, surgical site infections, catheter-associated infections, and sepsis [162,163]. Carbapenems are first-line antibiotics used to treat infections caused by multidrug-resistant gram (−) bacteria, particularly those that produce extended-spectrum β-lactamases [162]. The treatment of carbapenem-resistant *K. pneumoniae* (CRKP) infections is challenging because carbapenems are generally considered the antibiotics of last resort for severe *K. pneumoniae* infections. The most important genes responsible for conferring carbapenem resistance via carbapenemases are found in *K. pneumoniae*, rendering nearly all current treatment options ineffective [164]. Zhu et al. (2026) [65] developed a near-infrared (NIR) fluorescent probe, ACLE-S-HC, to detect carbapenemase activity and screened 288 plant extracts. *Mori Cortex* presented the highest inhibitory profile, with KWG identified as its most active component. Enzymatic assays showed that KWG inhibited the hydrolase activity of the carbapenemase enzyme with IC_50_ value of 1.322 ± 0.068 µM. Structural characterization and molecular docking simulations suggested a competitive binding model where KWG interacts at the catalytic site with a binding free energy of −25.32 ± 6.93 kcal/mol. Fluorescent imaging of live CRKP colonies treated with KWG revealed a decrease in the fluorescent signal, confirming enzyme inhibition *in vitro*. While the carbapenemase produced by CRKP rapidly degraded the antibiotic meropenem within 4 h, KWG administration extended the antibiotic’s half-life up to 12 h. When KWG (16 µg/mL) was combined with meropenem, the MIC of meropenem decreased from 16 µg/mL to 4 µg/mL, indicating a potential combination strategy to modulate carbapenem resistance.

#### 3.6.2. Antiviral Activity

Among the numerous infectious disease outbreaks humanity has to deal with, viral infections undoubtedly pose the greatest pandemic threat in the modern era [165]. Viruses possess a sophisticated molecular structure that allows them to evolve easily and thus survive in all species. Their sustained success stems from their ability to disrupt the host cell’s defense systems, which allows the virus to survive, replicate, and spread [166]. Over the past two centuries, viral pandemics have posed an increasing threat to public health worldwide. Some of the well-known public health threats are viral agents, including the highly pathogenic influenza viruses, Zika, Ebola, AIDS, dengue, and coronaviruses [167,168].

Coronaviruses include severe acute respiratory syndrome, Middle East respiratory syndrome, and especially the recent SARS-CoV variant, SARS-CoV-2. This variant caused the highly contagious, airborne, and dangerous coronavirus disease 2019 (COVID-19), which led to a pandemic affecting millions of people [167]. The majority of patients infected with the virus experience mildly moderate respiratory illness and recover without requiring special treatment. In the advanced stages of the disease, patients experience severe shortness of breath, decreased blood oxygen levels (hypoxia), lung damage, and various organ dysfunctions. Acute respiratory distress syndrome is the leading cause of death related to COVID-19 [169,170]. At the start of the pandemic, there was an urgent need to develop effective candidate drugs or therapeutic molecules against the novel virus, since there were no specific drugs or vaccines for its treatment. Therefore, various naturally occurring metabolites, such as flavonoids, have been identified and investigated as potential drug candidates for the treatment of COVID-19 [171]. Thabti et al. (2020) [77] aimed to elucidate the antiviral activity of KWG against human coronavirus (HCoV 229E) using cellular and pharmacological parameters. MTT analyses performed on L-132 lung epithelial cells showed that KWG at a dose of 5 µg/mL presented a high safety profile, maintaining a viability rate of 99.19%, while CC_50_ was 9.45 ± 0.55 µg/mL. While the viral control group sustained an 80.56% cytopathic destruction rate, the non-toxic dose of 5 µg/mL KWG restricted this cytopathic effect to 48.57%. Increasing the concentration to 10 µg/mL lowered the viral cytopathic effect to 2%. The half-maximal effective concentrations (EC_50_) of KWG for different MOI (multiplicity of infection) values (0.01, 0.1, and 1) as 0.11 ± 0.13 µg/mL, 0.56 ± 0.2 µg/mL, and 5.61 ± 0.67 µg/mL, respectively. The Selectivity Index (SI), obtained by dividing these values by cytotoxicity, was determined to be 86.73, 17.04, and 1.70 for KWG, respectively.

Dengue virus (DENV) infection is a significant global public health concern, especially in tropical and subtropical regions, where the primary vectors are *Aedes* mosquitoes [172]. However, dengue fever cases have increased 30-fold in the past 50 years, and outbreaks now occur annually across many continents, including the Americas, Asia, Europe, Africa, and Australia. According to the WHO, approximately half of the world’s population is at risk for DENV, with an estimated 100 to 400 million cases occurring each year [173]. DENV causes clinical symptoms ranging from mild, self-limiting febrile illness to serious, life-threatening conditions such as dengue hemorrhagic fever and dengue shock syndrome [172]. DENV induces autophagy, an evolutionary recycling and stress response mechanism of the host cell, to evade host immunity and maximize viral replication. It increases both intracellular viral RNA expression and extracellular virion production by preventing the fusion of autophagosomes with lysosomes [174]. In the study conducted by Limthongkul et al. (2023) [175], the antiviral activity and autophagy inhibition potential of the “Ka-003” molecule, a synthetic methylcyclohexene derivative structurally similar to KWG, against the DENV were investigated. In cell-based models utilizing HeLa and THP-1 human monocytic lines integrated with GFP-LC3 via the CRISPR-Cas9 system, Ka-003 restricted starvation-induced autophagosome formation (IC_50_ = 5.05 µM) in a concentration-dependent manner. This evaluation suggested that the compound acts by limiting autophagosome formation. In Western blot analyses, while GFP-LC3-II levels decreased in Ka-003-treated cells, the autophagy substrate P62 protein did not accumulate. To determine whether this structural pathway translates into antiviral potential, CRISPR-Cas9 GFP-LC3 knocked-in THP-1 cells were infected with DENV. Ka-003 restricted the production of newly formed infectious DENV particles in a concentration-dependent manner at low micromolar doses (IC_50_ = 2.01 ± 0.49 µM). qRT-PCR results confirmed an approximately 90% decrease in the number of viral RNA copies within the cell. Furthermore, in cellular safety assessments, Ka-003 exhibited low cytotoxicity against normal human renal HK-2 cells, HeLa cells, and THP-1 cells, with CC_50_ values of 32.05 ± 1.58 μM, 98.27 ± 5.34 μM, and 42.78 ± 3.11 μM, respectively.

Human norovirus is one of the leading causes of acute gastroenteritis worldwide, with an estimated 685 million cases annually. An average of 1.5 million people die each year from all causes of acute gastroenteritis worldwide, with 136,000 to 278,000 of those deaths related to norovirus infection. Young children, older adults, and immunocompromised individuals are especially susceptible to severe norovirus illness and its complications [176]. Currently, there is no effective medication available to prevent these infections. Therefore, exploring natural compounds for their treatment and prevention remains challenging and interesting [177]. For this purpose, KWG against murine norovirus (MNV), used as a surrogate for human norovirus, and its cytotoxic effect on RAW 264.7 (mouse leukemic macrophage) cell lines were investigated *in vitro*. KWG maintained a 92% cell viability profile at a concentration of 100 μM on RAW 264.7. Treatment of the MNV suspension with KWG induced a dose-dependent reduction in viral titers. While KWG concentrations of 1 μM and 10 μM yielded reductions of 0.3 and 0.4 logs, respectively, the maximum tested concentration of 100 μM achieved a 0.6-log reduction in MNV infectivity. Based on these preliminary results, the authors suggest that KWG may act against MNV by disrupting viral particles or by blocking the virus from binding to cell surface receptors [178].

#### 3.6.3. Antiparasitic Activity

Fish is a readily available and safe source of protein for humans, and fish parasitism poses a major threat to fish productivity by reducing growth rates, decreasing fish quality and quantity, and lowering reproductive rates [179]. The most commonly used agents against fish ectoparasites in aquaculture have harmful effects on both human health and the environment. Environmentally friendly, non-toxic natural compounds are becoming increasingly important as alternative agents in the control of fish ectoparasites [180]. For this purpose, the effect of KWG against fish ectoparasites was investigated in two separate studies. Liang et al. (2015) [47] tested the efficacy of KWG against the parasite *Ichthyophthirius multifiliis*, which causes white spot disease in freshwater fish. Against the infective theront form of the parasite isolated from goldfish, KWG demonstrated an antiparasitic effect with an EC_50_ value of 0.80 mg/L. At a concentration of 2 mg/L, KWG eliminated 100% of the theronts within 30.7 min. Increasing the concentration to 4 mg/L reduced the parasite elimination time by less than 2 min. In prophylactic evaluations, pre-treatment of theronts with 1 mg/L KWG for 2 h limited the subsequent fish infection prevalence from 100% to 3.3%. The authors suggest that the eight phenolic hydroxyl groups and one isopentene (prenyl) moiety in KWG’s structure may induce cell membrane perforations in the ciliate parasite. In the acute toxicity assay, the 96 h median lethal concentration (LC_50_) value of KWG for grass carp was determined to be 38.0 mg/L. This is approximately 50 times the dose required to kill the parasite (LC_50_ = 0.8 mg/L), indicating a laboratory-determined safety margin for the fish at therapeutic concentrations.

Liu et al. (2021) [76] also investigated the antiparasitic efficacy of KWG against *Neobenedenia girellae*, a pathogenic ectoparasite in marine fish. At concentrations of 125 mg/L and above, KWG eliminated 100% of adult parasites following an 8 h exposure. The 8 h LC_50_ value was determined to be 58.8 mg/L. Under these *in vitro* conditions, this lethal threshold presented lower values than those obtained for the reference phytochemicals curcumin (LC_50_ = 843.1 mg/L) and 10-gingerol (LC_50_ = 933.0 mg/L). Because *N. girellae* is a metazoan parasite with complex cellular organization, penetration of chemical compounds may be restricted compared to simpler protozoan models. These preliminary data suggest that KWG represents a potential natural alternative agent for evaluating ectoparasite control mechanisms.

#### 3.6.4. Antifungal Activity

Fungal pathogens cause constant damage to and loss of crops worldwide, whether in the field or in storage facilities. Additionally, certain fungal species, such as *Alternaria*, *Aspergillus*, *Fusarium*, and *Penicillium*, produce mycotoxins in food consumed by humans and/or animals [181]. In the study investigating the antifungal activities of KWG against some of these phytopathogenic fungi (*Botrytis cinerea*, *Penicillium expansum*, *Aspergillus niger*, *A. carbonarius*, and *A. terreus*), KWG exhibited a fungistatic profile against the tested species, with MIC_50_ values ranging from 16 to 64 μg/mL in the microdilution tests. The lowest inhibitory parameters were measured against *P. expansum* and *A. carbonarius* strains, presenting a MIC_50_ of 16 μg/mL and a MIC_100_ of 32 μg/mL. The study scientifically highlights that the prenyl groups in the structure of KWG confer a highly lipophilic character on the molecule, thereby enabling the compound to easily pass through the lipid cell membranes of fungal pathogens and penetrate the cell [81].

**Figure 9:**
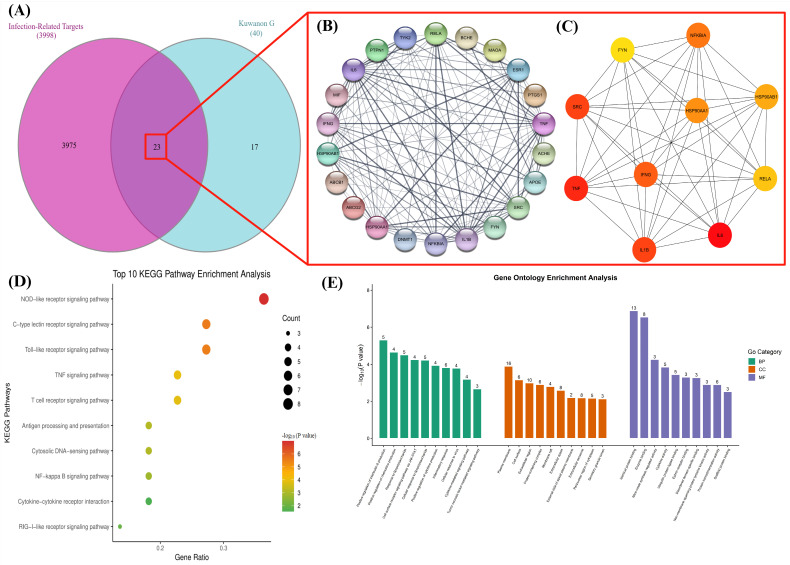
Network pharmacology analysis of Kuwanon G in infection-related targets. (**A**) Venn diagram showing the overlap between infection-related targets (pink, *n* = 3998) and KWG targets (blue, *n* = 40); 23 common genes were identified. (**B**) The protein–protein interaction (PPI) network was constructed using the STRING database and visualized with Cytoscape. (**C**) Hub genes in the network are colored according to their degree values, ranging from red to yellow. (**D**) KEGG pathway enrichment analysis is shown as a dot plot, where dot size represents the number of genes and the color indicates significance (*p*-value). (**E**) GO enrichment analysis is shown as bar graphs for the categories of biological process (BP), cellular component (CC) and molecular function (MF).

Based on computational screening, a comparison of infection-related target genes involved in antiparasitic, antifungal, antiviral, and antibacterial activities with KWG targets predicted a total of 23 common genes. This intersection indicates a theoretical overlap, suggesting that KWG could be evaluated as a computational candidate for multi-target mechanisms across different infection types. The PPI network constructed using these common genes shows a tight network of interactions between the target proteins. Analysis of the network topology suggests a theoretical model where genes critical to inflammatory and immune responses, such as *IL-6*, *TNF*, *IL-1β*, *IFN-γ*, *RELA*, and *NFKBIA*, occupy central hub positions. Concurrently, KEGG pathway enrichment analysis indicated that these common genes are primarily concentrated in biological pathways traditionally linked to host defense cascades, specifically NOD-like receptor, Toll-like receptor, C-type lectin receptor, TNF and T cell receptor signaling pathways. GO enrichment analysis reports that genes are particularly concentrated in biological processes related to inflammatory responses, cytokine-mediated signaling, immune system processes, and defense mechanisms against pathogens. These network pharmacology patterns suggest that KWG has theoretical capacity to modulate immune mechanisms involved in infection pathogenesis (Figure 9).

To evaluate these *in silico* projections, prior literature offers valuable checkpoints; indeed, our computational models conceptually parallel various empirically demonstrated antimicrobial mechanisms. Particularly in prior *in vitro* studies, KWG restricted the PtpB phosphatase of *M. tuberculosis*, which is a virulence factor that suppresses host immune cascades. This reported PtpB-NF-κB relationship, along with the prominent presence of RELA, NFKBIA, and NF-κB signaling pathways in our network analysis, exhibits a clear conceptual match across infection-related immune regulatory mechanisms [80]. Similarly, studies on MRSA have reported that KWG disrupts MRSA membrane integrity, impairs PMF, reduces intracellular ATP levels. Our network analysis suggested that inflammation-related targets, such as TNF-α, IL-6, and IL-1β, alongside the TNF signaling, Toll-like receptor, and cytokine-cytokine receptor interaction pathways, are theoretically associated with host responses modeled during infection [43,157]. The predicted targets and enriched KEGG/GO categories encompass core regulatory cascades, including NF-κB, NOD-like receptor, and C-type lectin receptor signaling, as well as antigen processing and presentation. When evaluated alongside the antibacterial, antituberculosis, and resistance-modifying effects reported in the literature, these *in silico* patterns conceptually parallel the known empirical context of KWG. Consequently, these findings indicate that infection-related signaling networks and host immune responses represent prominent directions for future validation studies.

In conclusion, while KWG exhibits a broad-spectrum *in vitro* antimicrobial layout, its clinical translation is strictly governed by systemic toxicity profiles and pharmacokinetic limitations. Safety screenings using mammalian cell lines indicate measurable cytotoxicity against HepG2 and Vero cells, alongside a noteworthy hemolytic activity against sheep red blood cells. Based on these cellular thresholds, the systemic administration of KWG remains restricted under current preclinical designs. Furthermore, *in vivo* evaluations indicate that the compound’s independent efficacy can be limited by its baseline tendency to bind mammalian cells. Therefore, these combined findings suggest that future development strategies for KWG should focus primarily on targeted local applications or topical formulations, such as wound healing or oral care agents, where its multi-target antimicrobial potential can be leveraged without systemic exposure risks.

### 3.7. Antioxidant Activity

Oxidative stress develops when there is an imbalance between the generation and accumulation of ROS and reactive nitrogen species (RNS) in cells and tissues and the capacity of biological mechanisms to remove these by-products. It arises from disturbances in the homeostatic processes that regulate pro- and antioxidant equilibrium [182]. The elevation of free radicals generated through biological oxidation leads to the disruption of intracellular protein structure and function, membrane impairment due to polyunsaturated fatty acid peroxidation, modifications of nucleic acid bases, chromosomal alterations including single-strand and double-strand DNA breaks, as well as DNA-protein cross-links, cell death, and oxidative damage to cellular structures and components, such as polysaccharide depolymerization and carbohydrate degradation. Molecules that can inhibit or postpone the oxidation of proteins, lipids, carbohydrates, and DNA in biological systems are termed antioxidants. Antioxidants function as a protective system for biomolecules and organisms against the detrimental effects of free radicals, mitigating or repairing the damage inflicted by ROS on target molecules, a process referred to as antioxidant defense [183]. Approaches that enhance the functioning of antioxidant mechanisms are considered important therapeutic strategies for both the prevention and treatment of many diseases [184].

Hsu et al. (2022) [60] evaluated the antioxidant activities of different solvent extracts of *M. alba* root bark and their isolated compounds. In superoxide radical scavenging assays, KWG exhibited a 50% scavenging concentration (SC_50_) value of 188.24 ± 19.07 µM, presenting limited independent activity compared to reference antioxidant morin (SC_50_ = 17.49 ± 3.43 μM). KWG did not show a prominent radical scavenging effect in parallel DPPH test (SC_50_ > 200 µM). According to the ABTS assay, KWG presented a radical scavenging threshold (SC_50_ = 9.28 ± 1.02 μM) lower than the synthetic positive control butylated hydroxytoluene (BHT; SC_50_ = 115.86 ± 25.14 µM). In terms of iron reduction capacity, KWG exhibited antioxidant power with a Trolox equivalent (TE) value of 3549.22 ± 160.65 mM/g when compared to BHT (TE = 4385.56 ± 78.88 mM/g).

In another study, the antioxidant capacities of compounds and subfractions isolated from the bark of *M. nigra* were determined using the *in vitro* ABTS radical scavenging test. KWG isolated from the ethyl acetate fraction exhibited a radical inhibition value of 87.11%, which conceptually aligns with the 87.5% inhibition rate observed for the ascorbic acid reference control. Structurally, KWG is a Diels–Alder type adduct formed via the condensation of a chalcone and dehydroprenylkuwanon C. Its structural precursor, kuwanon C, presented a lower inhibition rate (80.38%), suggesting that such Diels–Alder cyclization models are associated with altered radical scavenging parameters [83].

Beyond cell-free chemical assays, *in vitro* cell culture models evaluated the protective effects of KWG against induced endothelial injury. Jin et al. (2025) [72] examined the effects of the main compounds in mulberry twig extracts on HUVECs induced by ox-LDL. In cell viability screenings, KWG did not induce cytotoxicity at concentrations of 10 and 20 µM. While exposure to 80 μg/mL of ox-LDL reduced HUVEC viability to 77.57%, co-incubation with 20 μM KWG maintained a cell viability rate of 92.05%. When lipid peroxidation and antioxidant defense parameters were examined, the application of 20 µM KWG reduced the ox-LDL-induced MDA elevation from 5.67 nmol/mg to 3.60 ± 1.23 nmol/mg. Concurrently, SOD activity, which was suppressed by ox-LDL exposure, increased from 28.60 ± 7.00 U/mL to 54.37 ± 13.47 U/mL in the presence of KWG. In fluorometric cellular analyses, it was observed that KWG treatment reduced intracellular superoxide anion levels caused by oxidized LDL by 45.95% and total ROS levels by 49.27%; this demonstrates its ability to alleviate endothelial oxidative damage.

**Figure 10:**
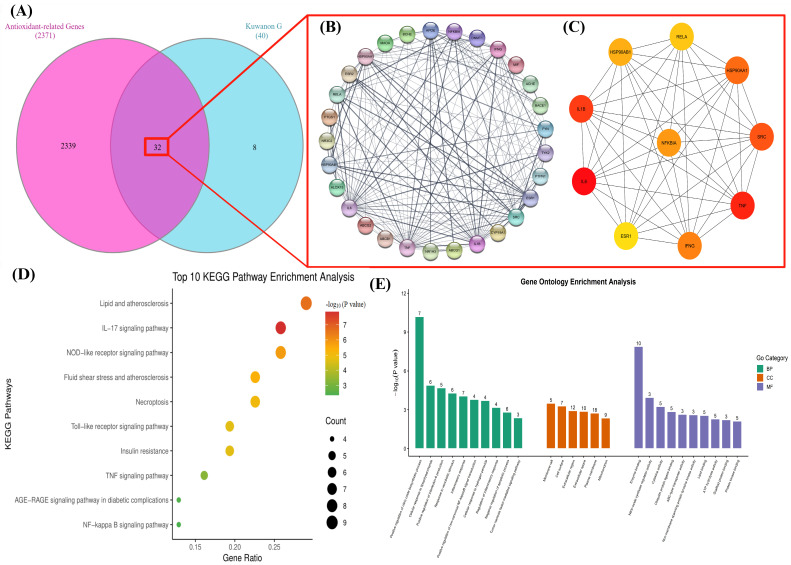
Network pharmacology analysis of Kuwanon G in antioxidant-related genes. (**A**) Venn diagram showing the overlap between antioxidant-related genes (pink, *n* = 2371) and Kuwanon G targets (blue, *n* = 40); 32 common genes were identified. (**B**) The protein–protein interaction (PPI) network was constructed using the STRING database and visualized with Cytoscape. (**C**) Hub genes in the network are colored according to their degree values, ranging from red to yellow. (**D**) KEGG pathway enrichment analysis is shown as a dot plot, where dot size represents the number of genes and the color indicates significance (*p*-value). (**E**) GO enrichment analysis is shown as bar graphs for the categories of biological process (BP), cellular component (CC) and molecular function (MF).

To contextualize these cellular mechanisms, a separate *in silico* network pharmacology screening mapped potential interaction models. A comparison between antioxidant-related genes and KWG targets predicted an overlap of 32 common genes. PPI network analysis showed that these 32 common genes exhibited a linked structure, with specific nodes playing a central role within the network. The prominence of genes such as *TNF*, *IL-6*, *IL-1β*, *RELA*, and *NFKBIA* as hub genes suggest a theoretical model. In this model, the antioxidant effect of KWG is largely shaped by the interaction between inflammation and oxidative stress. These genes are key components of the NF-κB signaling pathway, which plays a critical role in ROS production and the regulation of cellular defense mechanisms. KEGG pathway enrichment analysis results indicated that these common genes are concentrated primarily in specific defense pathways. These are closely related to inflammation and oxidative stress, such as the IL-17, TNF, Toll-like receptor, and NF-κB signaling pathways. In the GO analysis, the computational targets were grouped under biological processes linked to oxidative stress responses, inflammatory pathways, and cytokine-mediated signaling. These network configurations suggest a hypothetical profile where KWG targets the inflammation-oxidative stress axis through multi-pathway mechanisms (Figure 10). Notably, the target genes and biological pathways identified through this network analysis have not been directly linked to existing experimental data in the literature. Nevertheless, these *in silico* results represent potential areas of research that provide baseline targets for future investigators to further clarify the comprehensive mechanisms of action of KWG.

Evaluating the therapeutic potential of KWG requires contrasting its empirical antioxidant efficiency against baseline cytotoxicity thresholds. KWG exhibits limited independent radical scavenging activity compared to reference antioxidants like morin. In cell culture models, the compound mitigates ox-LDL-induced endothelial injury by increasing SOD activity, suppressing MDA accumulation, and reducing intracellular ROS levels. Despite these positive *in vitro* results, the transition of KWG into systemic antioxidant therapies is restricted by safety boundaries, including measurable cytotoxicity in cell lines. These parameters suggest that high systemic doses required to achieve effective antioxidant concentrations may induce adverse cellular interactions. Consequently, future research directions should focus on validating these antioxidant mechanisms within localized vascular injury models or targeted microenvironments. Investigating KWG within localized systems allows for the utilization of its cellular cytoprotective effects while avoiding the toxicological risks associated with high systemic exposure.

### 3.8. Anticancer Activity

Cancer is the aberrant proliferation of cells within the body and constitutes the primary cause of mortality, representing the most significant public health threat worldwide. The concerning increase in the death rate from this disease draws attention to the need for identifying effective anticancer drugs to mitigate its mortality rate. The identification of innovative and potent anticancer medicines from natural sources has been a primary focus in pharmaceutical research. Over 60% of modern anticancer drugs have been derived from natural sources. Plants and their derivatives have significantly contributed to the development of potent anticancer medicines. Numerous plant-derived bioactive chemicals are currently undergoing clinical investigation for cancer treatment, contingent upon their specific activity. Insights from pharmaceutical research investigations could produce alternative medication development processes from natural sources that are cost-effective, more dependable, and safe for consumption [185].

KWG was isolated from the ethyl acetate (EtOAc) fraction of the methanol extract of the bark of *M. nigra* L. The antiproliferative activity of KWG was evaluated against HepG2 and breast cancer (MCF-7) cells using the MTT assay. IC_50_ values of KWG were determined to be 34.35 ± 1.52 µM for the HepG2 and 35.79 ± 1.50 µM for the MCF-7 cells. The activity exhibited by KWG in the MCF-7 line (35.79 µM) was at a level quite similar to that of 5-fluorouracil (5-FU; 34.17 µM), the standard chemotherapy drug used as a control [186].

The effects of KWG on the proliferation, apoptosis, migration, and invasion of gastric cancer cells (MGC 803, HGC 27, AGS, and SGC-7901) were evaluated *in vitro* and *in vivo*. KWG restricted the proliferation of these cells in a concentration-dependent manner. The cell colony formation assay demonstrated that, compared to the control group, 20 and 40 μmol/L KWG limited the number of MGC 803 and HGC 27 cell colonies compared to 5 and 10 μmol/L KWG. At the molecular level, KWG at different concentrations increased cleaved-caspase 3 and Bax expression while decreasing Bcl2 levels in MGC 803 and HGC 27 cells, confirming apoptosis. Transwell migration and invasion assays demonstrated a decrease in the migration and invasion rates of all four gastric cancer cell lines following KWG administration at various concentrations. Immunoblot analysis indicated that the expression levels of MMP2 and MMP9 proteins, which are associated with migration and invasion, decreased in MGC 803, HGC 27, AGS, and SGC-7901 cells following KWG treatment at various concentrations. Following KWG treatment at various concentrations, the expression of p-PI3K, p-AKT, and p-mTOR was inhibited in MGC 803 and HGC 27 cells; IGF-1 enhanced KWG’s inhibitory effect on the cells. Compared to the KWG group, MMP2 and MMP9 levels in gastric cancer cells were increased in the IGF-1 group. KWG inhibited the proliferation, migration, and invasion of gastric cancer cells by inhibiting the PI3K/AKT/mTOR pathway. In *in vivo* experiments, tumors were implanted into the backs of nude mice, and the application of KWG at various concentrations led to a reduction in the weight and volume of the transplanted tumors compared to the control group. Immunohistochemical analyses showed a decrease in the number of Ki-67-positive cells following KWG treatment was achieved at different concentrations. When TUNEL staining was applied to the transplanted tumors, an increase in the number of TUNEL-positive cells was observed following KWG treatment. Consistent with the *in vitro* findings, KWG application in the xenograft model increased levels of cleaved-caspase 3 and Bax in transplanted tumors while suppressing Bcl-2 levels. Compared to the control group, KWG inhibited the expression of MMP2 and MMP9 and reduced levels of p-PI3K, p-AKT, and p-mTOR in transplanted tumors [187].

**Figure 11:**
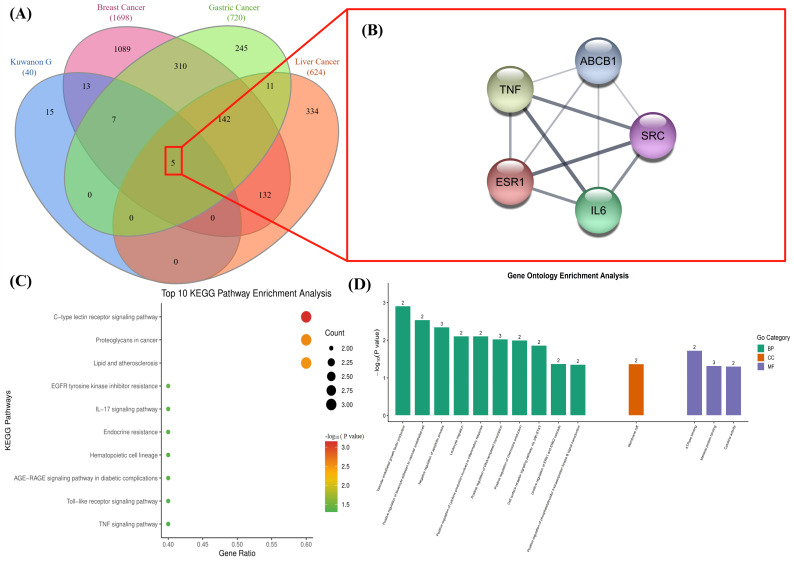
Network pharmacology analysis of Kuwanon G in breast, gastric and liver cancer. (**A**) Venn diagram showing the overlap between breast cancer targets (pink, *n* = 1698), gastric cancer (green, *n* = 720), liver cancer (orange, *n* = 624) and KWG targets (blue, *n* = 40); 5 common genes were identified. (**B**) The protein–protein interaction (PPI) network was constructed using the STRING database and visualized with Cytoscape. (**C**) KEGG pathway enrichment analysis is shown as a dot plot, where dot size represents the number of genes and the color indicates significance (*p*-value). (**D**) GO enrichment analysis is shown as bar graphs for the categories of biological process (BP), cellular component (CC) and molecular function (MF).

To contextualize these multi-target activities, a separate *in silico* network pharmacology screening mapped potential cross-cancer interaction models. A comparison of target genes associated with breast, gastric, and liver cancers with KWG targets predicted an overlap of 5 common genes This limited overlap indicates that KWG may interact through common but selective molecular targets across cancer types. Topology analysis of the constructed PPI network highlighted interactions between the *TNF*, *IL-6*, *SRC*, *ESR1*, and *ABCB1* genes. These specific genes are linked to inflammation, cellular proliferation, hormone signaling, and drug resistance mechanisms. KEGG pathway enrichment analysis indicated that the common genes are involved in the C-type lectin receptor, proteoglycans, and lipid and atherosclerosis-related pathways. The network also highlighted pathways associated with disease progression and resistance, including EGFR tyrosine kinase inhibitor resistance, IL-17 signaling and TNF signaling pathways. In GO enrichment analysis, the computational target genes were concentrated in the BP category. These processes involve the regulation of vascular endothelial growth factor production, positive regulation of leukocyte adhesion to vascular endothelial cells, negative regulation of apoptotic processes, and leukocyte migration. Taken together, these network configurations suggest a hypothetical profile where KWG interacts with pathways regulating inflammation, proliferation, angiogenesis, and signal transduction (Figure 11).

Notably, the target genes and biological pathways identified through this network analysis have not been directly linked to existing experimental data in the literature. In line with critical evaluation standards, these computational patterns cannot be interpreted as definitive biological evidence of therapeutic efficacy. Nevertheless, these *in silico* results represent potential areas of research that provide baseline targets for future investigators to further clarify the mechanisms of action of KWG.

In conclusion, the clinical evaluation of KWG requires comparing its empirical anticancer efficacy directly against its documented cytotoxicity thresholds. KWG exhibits targeted antiproliferative activities against HepG2 and MCF-7 cell lines, alongside a concentration-dependent capacity to induce apoptosis and reduce tumor weights and volumes in gastric xenograft designs. However, these experimental oncological profiles are limited by the compound’s broader cellular safety parameters. Preclinical screens demonstrate measurable cytotoxicity in standard human cell lines. Based on these paired findings, the high doses evaluated in animal cancer models must be carefully weighed against the established toxicological thresholds observed in non-cancerous cells.

### 3.9. Enzymatic Activity

Tyrosinase is an oxidoreductase that has two copper ions in its active site and is commonly found in many organisms, from bacteria to eukaryotes. It catalyzes the *o*-hydroxylation of monophenols to diphenols (monophenolase activity) and the oxidation of diphenols to quinones (diphenolase activity). Tyrosinase plays an important role in the enzymatic browning of fruits or fungi and in melanin synthesis in mammals [188]. It is involved in the first two rate-limiting steps of melanin production in the skin: the initial hydroxylation of *_L_*-tyrosine to *_L_*-3,4-dihydroxyphenylalanine (*_L_*-DOPA), followed by the oxidation of *_L_*-DOPA to *_L_*-dopaquinone [189]. Melanin is produced in melanosomes, which are lysosome-like organelles within skin melanocytes, and is the primary cause of pigmentation in the skin, eyes, and hair. Excessive production and accumulation of melanin lead to hyperpigmentation disorders, including post-inflammatory hyperpigmentation, melasma, freckles, and malignant melanoma [190]. Tyrosinase is considered an important target for pigmentation disorders because it acts as both an initiator and a rate-limiting factor in melanin biosynthesis. Therefore, anti-tyrosinase agents will have broad application potential in the agricultural, medical, and cosmetic industries [188].

In a study conducted for this purpose, the tyrosinase potentials and structure–activity relationships (SAR) of compounds isolated from the extract of *M. nigra* roots were evaluated. In tyrosinase inhibition assays, KWG exhibited very weak or undetectable inhibitory activity against mushroom tyrosinase (IC_50_ > 200 μM). SAR analyses indicated that this limited baseline activity is linked to the presence of an isoprenyl group at the 3rd position of the flavonoid skeleton. In contrast, the structural analog kuwanon H shows alternative dynamics due to an additional isoprenyl substitution at the 24th position of its E ring [82]. In a parallel investigation using components from *M. australis*, KWG similarly displayed minimal efficacy, maintaining an IC_50_ threshold greater than 200 μM. The authors suggested that the tyrosinase inhibitory activities of these compounds depend on the specific substitution profiles of the structural rings rather than their fundamental skeletons. Consequently, they concluded that the low tyrosinase activity of KWG is due to the presence of an isoprenyl group at the 3rd position of the flavonoid skeleton [79].

In a tyrosinase diphenolase assay evaluating components from Greek flora, including *M. alba*, KWG presented a measured IC_50_ value of 27.5 ± 1.2 μM, which was numerically higher than the reference control kojic acid (IC_50_ = 16.1 μM). Remarkably, *in vivo* experiments conducted on zebrafish have shown that a specific chromatographic fraction (CPC F33) containing the compounds 2,4,3-trihydrodihydrostilbene, morasin M, and KWG restricted melanogenesis, suggesting potential multi-component interactions within the natural plant matrix [75].

Koirala et al. (2018) [66] conducted a comprehensive study specifically to clarify the conflicting results regarding the effects of KWG on the tyrosinase enzyme. The activity of KWG against mushroom tyrosinase was tested using *_L_*-tyrosine and *_L_*-DOPA substrates. KWG showed inhibitory activity (IC_50_ = 67.6 µM) against *_L_*-tyrosine oxidation. Against the *_L_*-DOPA substrate, it demonstrated an IC_50_ value of 44.04 µM, compared to the kojic acid reference parameter of 79.0 µM. Enzyme kinetic analyses indicated a competitive and reversible inhibition mode, showing *K*_i_ value of 18.7 µM. Based on the molecular docking results, four hydrogen bonds between the hydroxyl groups of KWG and the Val283, His263, Gly281, and Ser282 residues within the active site, as well as van der Waals interactions with the copper ions in the core, were modeled. SAR analyses suggest that the methyl cyclohexene ring in the structure of KWG forms strong hydrophobic interactions with the catalytic residues of the enzyme, and therefore, this functional group is directly responsible for its tyrosinase inhibitory activity.

Another study investigated the anti-tyrosinase activities of bioactive components, including KWG, which was isolated from the root bark of *M. alba*. KWG demonstrated moderate activity on the tyrosinase, with an IC_50_ value of 100.40 ± 9.62 μM, while the arbutin reference control exhibited an IC_50_ of 206.08 ± 36.50 μM. According to molecular docking analysis supporting the experimental data, the binding affinities of KWG and arbutin to the active pocket of the tyrosinase were calculated as −6.4 kcal/mol and −6.1 kcal/mol, respectively [60].

To evaluate microbial enzyme interactions linked to drug metabolism, multiple *in vitro* assays analyzed the inhibitory capacity of KWG against gut bacterial enzymes. The β-glucuronidase (β-GUS) enzyme produced by gut bacteria, and particularly the *E. coli*-derived form (EcGUS), plays a critical role in human health and drug metabolism. β-GUS is responsible for hydrolyzing various compounds that have been converted into the glucuronide form (inactivated) in the body, thereby converting them back into the free aglycone (active/toxic form). This process increases the local concentration of toxic compounds in the intestinal mucosa, leading to tissue damage. It has been noted that high β-GUS activity in the intestine contributes to the development of various diseases, such as colitis, liver disorders, and colorectal cancer. This enzyme is identified as the primary cause of severe diarrhea and intestinal toxicity caused by the anticancer drug irinotecan (CPT-11). The enzyme converts SN38G, the inactive metabolite of the drug, back into the active toxic form, SN38, in the intestine. In diseases such as colon cancer, β-GUS activity in the intestinal flora has been observed to be significantly higher than in healthy individuals. EcGUS is the most abundant type among β-glucuronidases produced by intestinal bacteria. *E. coli* is predominant for the production of “loop-β-glucuronidase” (L-GUS) during intestinal stress or colonic diseases. EcGUS is recognized as a key therapeutic target for alleviating drug-induced intestinal toxicity and maintaining intestinal health. Identifying natural compounds capable of strongly inhibiting EcGUS is essential for reducing chemotherapy side effects and preventing intestinal diseases. In substrate hydrolysis screenings, KWG restricted the concentration-dependent hydrolysis of 4-nitrophenyl β-D-glucuronide (PNPG) catalyzed by EcGUS. The IC_50_ value of KWG was determined to be 2.37 ± 0.11 µM, compared to the reference control D-glucaric acid-1,4-lactone (IC_50_ = 42.81 µM). Time-dependence assays indicated that the enzyme-inhibiting power of KWG is not time-dependent, indicating that it is not an inactivator that permanently inactivates the enzyme but rather a reversible inhibitor. Lineweaver–Burk plot analyses demonstrated a non-competitive inhibition pattern, indicating a calculated *K*_i_ value of 3.74 µM. Molecular docking simulations aligned with these kinetic findings, predicting that KWG localizes to an allosteric ligand-binding site rather than binding directly to the catalytic active pocket of the EcGUS enzyme. Parallel *in vitro* screenings indicated that KWG restricted (IC_50_ approximately 1 µM) not only EcGUS but also human carboxylesterase 2 (hCE2), another key target that triggers the intestinal toxicity of anticancer drugs such as CPT-11. These combined enzymatic profiles suggest that the compound targets microenvironmental elements associated with chemotherapeutic intestinal side effects, establishing a framework for further preclinical validation [191].

In another study, Wei and colleagues investigated the inhibitory effects of KWG on the *E. coli* β-glucuronidase (EcoGUS) and *S. pasteuri* 3I10 β-glucuronidase (SpasGUS) enzymes. In this study, PNPG, a synthetic substrate, was used to measure the activity of bacterial β-glucuronidase enzymes. Under normal conditions, the β-glucuronidase enzyme hydrolyzes PNPG, releasing yellow-colored p-nitrophenol (PNP) and glucuronic acid. The amount of PNP formed serves as a measure of the enzyme’s activity. Against EcoGUS, KWG presented an IC_50_ value of 1.6 ± 0.1 μM and *K*_i_ of 0.59 ± 0.27 μM, displaying a mixed-type inhibition profile. Against SpasGUS, the compound exhibited an IC_50_ parameter of 0.98 ± 0.25 μM and a *K*_i_ of 1.75 ± 0.79 μM, also maintaining a mixed-type inhibition design. The fact that KWG exhibits mixed-type inhibition indicates that the compound can bind to both the active site and the allosteric site of the enzyme. In this study, the interactions of KWG with the EcoGUS and SpasGUS enzymes were characterized using molecular docking methods with MOE software. In the EcoGUS simulation, the benzopyranil moiety of KWG positioned within the allosteric binding pocket, modeling hydrogen bonds with the Lys157 and Gln158 residues. Molecular simulations indicated that KWG targets the allosteric pocket of SpasGUS, which exhibits greater structural proximity to its catalytic active site compared to the layout observed in EcoGUS. In the SpasGUS configuration, the phenolic hydroxyl groups of KWG modeled three distinct hydrogen bonds with the Phe162, Val369, and Glu370 residues, alongside hydrophobic interactions with Phe367 and Ile565. These structural simulations suggest that the phenolic groups and the benzopyran ring within the prenylflavanonol skeleton facilitate allosteric binding conformations across these bacterial enzymes [192].

Xanthine oxidase (XO) is an enzyme that plays a critical role in purine metabolism in the human body and facilitates the conversion of purines into uric acid. Hyperuricemia, defined as elevated levels of uric acid resulting from the activity of this enzyme, causes gout, a condition characterized by severe pain and swelling. If left untreated, this condition can lead to serious consequences such as knee damage, joint deformities, and kidney failure. Substances that inhibit the XO lower uric acid levels in the body; therefore, these inhibitors are a primary class of medications for the prevention and treatment of gout and other conditions associated with hyperuricemia. Commonly used XO inhibitors, such as allopurinol and febuxostat, may have adverse side effects including skin rash, nausea, vomiting, kidney failure, and Stevens-Johnson syndrome. In enzyme inhibition assays, KWG isolated from the ethyl acetate fraction of *M. alba* bark presented an IC_50_ value of 52.41 µg/mL against XO. Molecular docking studies mapped the binding dynamics within the catalytic zone, predicting that the hydroxyl groups of KWG form hydrogen bonds with critical active site residues, specifically Glu802, Arg880, and Thr1010. Additionally, modeling suggested a π-π stacking interaction between the phenyl ring of KWG and the Phe914 residue of the enzyme, indicating a competitive inhibition mechanism [69].

Nitroreductases (NTR) are enzymes commonly found in microorganisms within the human gastrointestinal tract, particularly in the colon. Gut bacterial nitroreductases are closely associated with the intestinal toxicity of nitroaromatic compounds found in foods, medications, or environmental pollutants in the intestine. These enzymes reduce nitro groups, converting them into mutagenic and enterotoxic N-nitroso or N-hydroxy intermediates. These intermediates can cause DNA damage and promote the progression of colon cancer. It has been reported that nitroreductase expression in the feces and intestinal contents of normal samples is significantly higher than in colon cancer patients or models, and this increases intestinal toxicity. Consequently, blocking the nitroreductases expressed by gut microbes has emerged as a promising strategy to diminish mutagenic compounds in the colon and avoid intestinal illnesses. Chen et al. (2022) [193] investigated the inhibitory effects of KWG on two kinds of gut bacterial nitroreductases, EcNfsA and EcNfsB, with nitrofurazone (NFZ) as substrate and NADPH as an electron donor. In enzymatic evaluations, The IC_50_ value of KWG against EcNfsA was determined to be 1.60 ± 0.082 μM and *K*_i_ 0.67 ± 0.098 μM, while the IC_50_ value against EcNfsB was 3.98 ± 0.64 μM and the *K*_i_ value 6.59 ± 1.59 μM. Since KWG restricted both EcNfsA and EcNfsB enzymes, it has been defined as a “dual inhibitor”. KWG exhibited a more effective inhibitory performance than positive control dicumarol against both enzymes. Kinetic analyses demonstrated that KWG acts as a competitive and reversible inhibitor for both enzymes, indicating that the compound directly competes with the NFZ substrate for binding coordinates within the catalytic active sites. Molecular docking studies were conducted to elucidate how KWG interacts with critical residues in the active sites of enzymes. Within the EcNfsA active site, the phenolic hydroxyl and carbonyl groups of KWG modeled hydrogen bonds with Asn134, His69, Gln67, and Gly65 residues, while the C6–C3 ring displayed a π-π stacking interaction with the FMN-360 cofactor. Within the EcNfsB structure, KWG modeled hydrogen bonds with Arg121, Glu102, and Lys14, alongside hydrophobic alkyl and π-alkyl interactions with Lys14, Phe70, and Ala116 residues. In summary, the low oral bioavailability of KWG suggests a localized mechanism within the gastrointestinal tract. Due to restricted systemic absorption, the compound is hypothesized to maintain localized concentrations within the intestinal lumen, potentially preventing the conversion of nitroaromatic xenobiotics into toxic intermediates. Mechanistically, structural data indicate that the ‘pentenyl’ moiety is critical for this interaction, potentially anchoring the compound to the hydrophobic regions of the enzyme and interfering with normal substrate binding.

The human carboxylesterase 2 (hCE2) enzyme is a member of the serine hydrolase superfamily found in the lumen of the endoplasmic reticulum in various mammalian cells. It is one of the major carboxylesterase isoforms in humans and belongs to the carboxylesterase (CE) gene family. This enzyme is primarily expressed in the small intestine and tumor tissues in humans. As a Phase I drug-metabolizing enzyme, it plays a critical role in the biotransformation of both endogenous and exogenous compounds, converting them into polar products that facilitate their elimination. It plays a significant role in the oral bioavailability of prodrugs. It directly affects the treatment outcomes of anticancer agents with an ester structure (e.g., capecitabine and CPT-11/irinotecan). CPT-11 is hydrolyzed by hCE2 to form its active metabolite, SN-38. The accumulation of SN-38 in the intestines is considered the primary cause of severe delayed diarrhea observed in CPT-11 treatment, which can be life-threatening. The use of potent inhibitors of hCE2, such as compounds derived from white mulberry root bark, may alleviate this serious diarrhea side effect caused by anticancer drugs and improve patients’ quality of life. While hCE2 plays a key role in the activation or elimination of drugs in the body, its activity in the intestines is also directly implicated in the side effects of certain cancer treatments. In enzyme inhibition assays, the IC_50_ value of KWG for human hCE2 activity was measured to be 1.14 μM. Under these conditions, this parameter was calculated to be approximately 18 times lower than that of the antidiarrheal reference loperamide and 6 times lower than the esterase inhibitor BNPP. Lineweaver–Burk and Dixon plot analyses indicated a non-competitive inhibition pattern, demonstrating that the compound interacts with a binding site outside the catalytic active pocket where the substrate coordinates. *K*_i_ value for KWG is 1.09 μM. These kinetic data indicate that KWG limits the enzymatic conversion of CPT-11 to SN-38, its active and toxic metabolite, under the test conditions [67].

Adenosine triphosphate (ATP)-citrate lyase (ACL) is a key enzyme that plays a critical role in metabolic processes in the body. ACL links carbohydrate metabolism with lipid metabolism. This enzyme converts citrate into acetyl-CoA, which is used in the biosynthesis of both cholesterol and fatty acids. When the ACL is inhibited, the supply of acetyl-CoA—which is essential for cholesterol production—is cut off. For this reason, ACL is identified as an important drug target for the treatment of metabolic disorders such as hyperlipidemia. It has been observed that inhibiting the ACL in the liver provides protection against fatty liver disease and improves hyperglycemia. The discovery of new plant-derived agents capable of inhibiting ACL is one of the focal points of current scientific research. Zhou et al. (2025) [73] investigated the effect of KWG obtained from the ethyl acetate fraction of an ethanol extract of dried branches of *M. alba* on the ACL. In the enzyme assay, the IC_50_ value of KWG against ACL was determined to be 4.1 ± 0.3 μM. For comparison, the reference ACL inhibitor BMS 303141 presented an IC_50_ value of 0.3 μM. These data lines indicate that KWG limits the *in vitro* catalytic function of ACL, restricting the cellular enzymatic framework required for lipid and cholesterol production.

In conclusion, KWG exhibits multi-target enzymatic modulation *in vitro*, presenting distinct concentration-dependent inhibitory patterns against both mammalian and microbial enzymes. However, the translation of these parameters into functional biological systems is strictly constrained by the compound’s absorption and safety thresholds. Pharmacokinetically, the low oral bioavailability of KWG limits its therapeutic activity depending on the anatomical site of the target enzymes. Because the compound exhibits restricted systemic absorption, it remains localized in higher concentrations within the intestinal lumen. This localized presence facilitates interactions with gut microbial targets such as EcGUS and nitroreductases. Mechanistically, structural data indicate that the ‘pentenyl’ moiety is critical for this interaction, potentially anchoring the compound to the hydrophobic regions of the enzyme and interfering with normal substrate binding. Conversely, this lack of adequate systemic absorption restricts the delivery of active fractions to deeper tissues, thereby limiting its practical efficacy against systemic targets like liver ACL or vascular XO. While KWG displays calculated inhibitory kinetics against the target proteins, these parameters are established outside complex physiological metabolic networks. Consequently, future preclinical evaluations must focus on tracking the stability and effective concentrations of KWG directly within matching biological models to determine whether these multi-target enzyme interactions translate into consistent physiological results.

## 4. Materials and Methods

### 4.1. Literature Searching Strategy

This comprehensive review evaluated the existing scientific literature regarding the chemical structure, biosynthesis, pharmacological activities, and molecular mechanisms of action of Kuwanon G (KWG) isolated from *Morus* species. To ensure a rigorous and transparent data collection process, systematic electronic searches were executed across the PubMed, Scopus, ScienceDirect, Web of Science, and Google Scholar databases. The literature retrieval covered peer-reviewed studies published from 1980 to 2026, with the final comprehensive database update executed on 15 April 2026. No language restrictions were applied during the initial retrieval process. The standardized search strings incorporated explicit Boolean operators (AND, OR) to refine the target literature pool. The core search matrix utilized combinations of the primary keyword with specific functional and pharmacological terms: “Kuwanon G”, “Kuwanon G” AND “Morus”, ”Kuwanon G” AND (“natural products” OR “biological activity” OR “biosynthesis”), ”Kuwanon G” AND (“anti-cardiovascular” OR “cardioprotective” OR “anti-diabetic” OR “antidiabetic”), “Kuwanon G” AND (“anti-inflammatory” OR “anti-allergic” OR “anti-asthmatic” OR “antioxidant”), “Kuwanon G” AND (“neuroprotective” OR “anti-obesity” OR “anticancer” OR “enzymatic activity”), “Kuwanon G” AND (“antibacterial” OR “antiviral” OR “antiparasitic” OR “antifungal”).

### 4.2. Inclusion and Exclusion Criteria

To ensure methodological consistency and guarantee alignment with the primary objectives of the review, the identified articles were screened according to predetermined eligibility criteria. Inclusion Criteria: (1) *in vitro*, *in vivo*, *ex vivo*, and *in silico* experimental model systems directly evaluating the biological activities of KWG; (2) studies utilizing established human cell lines, primary human cells, standard laboratory animals (including mice, rats, and rabbits), or tissues derived from them; (3) studies focusing on KWG isolated directly from *Morus* species, its identified active metabolites, or verified synthetic analogs; and (4) original research articles detailing or modeling specific pharmacological pathways and molecular mechanisms of action. Exclusion Criteria: (1) Studies that did not satisfy the core inclusion parameters or lacked primary empirical data regarding KWG; (2) articles restricted solely to clinical pharmacokinetics or baseline agronomic reporting; (3) secondary literature, including case reports, letters, editorials, book reviews, commentaries, conference abstracts, and patents; and (4) studies where the full-text manuscript was completely unavailable or inaccessible.

To maintain a clear and objective screening workflow, we followed a practical, multi-stage evaluation process. In the first stage, two authors reviewed all identified titles and abstracts based on our specific inclusion and exclusion criteria. Following this initial look, we gathered the full-text versions of all relevant papers for a deeper check. The corresponding author then coordinated the final evaluation of these selected manuscripts. When questions or different interpretations arose about whether a study fits the review, we resolved them through targeted technical discussions. Since this paper serves as a comprehensive overview rather than a strict statistical meta-analysis, our primary goal during filtering was to ensure we covered as many relevant biological pathways of KWG as possible.

### 4.3. Identification of Target Genes Associated with KWG

Target genes associated with Kuwanon G (KWG) were obtained using the keyword “Kuwanon G” from the Comparative Toxicogenomics Database (CTD) (https://ctdbase.org/), Target Net (http://targetnet.scbdd.com) and Swiss Target Prediction (https://www.swisstargetprediction.ch/) databases. The SMILES structure and PubChem information of Kuwanon G were used as input for target prediction analyses in TargetNet and SwissTargetPrediction. In SwissTargetPrediction, targets with non-zero probability values were retained for further analysis. All predicted and experimentally validated targets were restricted to *Homo sapiens* to ensure species consistency. Gene lists obtained from the three databases were merged; duplicate genes were removed and resulting in a total of 40 genes included in the analysis [194,195,196].

### 4.4. Identification of Disease-Related Target Genes

The genes associated with the diseases examined in this study were obtained from the GeneCards (https://www.genecards.org/), Comparative Toxicogenomics Database (CTD) (https://ctdbase.org/), and DisGeNET (https://disgenet.com/) databases. To improve the specificity of disease-related gene and reduce data heterogeneity, disease-specific keywords were used during database searches according to the biological activities investigated in this study. For anti-cardiovascular effects, the keywords “myocardial infarction”, “hypertension”, and “atherosclerosis” were used. For anti-inflammatory, anti-allergic, and anti-asthmatic effects, the terms “asthma”, “chronic obstructive pulmonary disease (COPD)”, “acute bronchitis”, “chronic bronchitis”, “acute dermatitis”, “ulcerative colitis”, and “Crohn’s disease” were applied. Neuroprotective effect-related genes were retrieved using the keywords “Alzheimer’s disease”, “Parkinson’s disease”, and “diabetic encephalopathy”, while “diabetes mellitus” was used for antidiabetic activity. Antimicrobial activity-related genes were identified using the terms “bacterial infection”, “viral infection”, “fungal infection”, and “parasitic infection”. Cancer-related genes were obtained using the keywords “liver cancer”, “breast cancer”, and “gastric cancer” instead of the broader term “cancer”. For anti-obesity activity, the keyword “obesity” was used, whereas antioxidant activity-associated genes were retrieved using the terms “reactive oxygen species”, “oxidative stress”, and “antioxidant”. All retrieved genes were restricted to *Homo sapiens* to ensure species consistency. Gene lists obtained from the databases were merged, duplicate genes were removed, and final disease-associated gene sets were generated for subsequent analyses. The use of disease-specific terminology aimed to improve the biological relevance and reliability of the retrieved gene sets [197,198].

### 4.5. Identification of Common Target Genes and Venn Diagram

Identification of common target genes between Kuwanon G (KWG) and disease-associated genes was performed through overlap analysis using InteractiVenn (https://www.interactivenn.net/). The KWG-related target gene set and disease-associated gene sets were uploaded to the platform in gene symbol format, and intersections between datasets were identified and visualized using Venn diagrams. Shared genes representing the overlapping targets between KWG and disease-associated gene sets were identified and evaluated as potential therapeutic targets for subsequent protein–protein interaction (PPI) network and enrichment analyses [199].

### 4.6. PPI Network Analysis

Protein–protein interaction (PPI) analysis was performed to evaluate the interactions among the common target genes shared between KWG-associated targets and disease-related genes. The common target genes were uploaded to the STRING database (https://string-db.org/), with the organism restricted to *Homo sapiens* to ensure species consistency. A minimum required interaction score of 0.400 (medium confidence) was applied to improve the reliability of predicted interactions, and disconnected nodes were excluded from the network.

The generated PPI network was imported into Cytoscape software (version 3.10.4) for network visualization and topological analysis. Nodes with a degree value of 0 were excluded from the PPI network to eliminate disconnected nodes and improve network interpretability. The resulting network was subsequently analyzed to identify biologically important nodes within the interaction network. Hub genes were identified using the CytoHubba plugin based on the Maximal Clique Centrality (MCC) ranking algorithm, and the top 10 genes with the highest MCC scores were defined as core target genes [200,201].

### 4.7. GO and KEGG Pathway Enrichment Analysis

Gene Ontology (GO) and Kyoto Encyclopedia of Genes and Genomes (KEGG) pathway enrichment analyses were performed to investigate the functional characteristics and biological pathways associated with the common target genes. GO analysis was conducted under three categories, including biological process (BP), molecular function (MF), and cellular component (CC). Functional enrichment analyses were carried out using the DAVID Bioinformatics Resources platform (https://davidbioinformatics.nih.gov), with the organism restricted to *Homo sapiens*. The enrichment analysis parameters were set with a threshold of 0.05. Statistical significance was evaluated based on both raw *p*-values and false discovery rate (FDR) correction, and only GO terms and KEGG pathways with *p* < 0.05 and FDR < 0.05 were considered significantly enriched. The most significant GO terms and KEGG pathways were selected for subsequent analyses and interpretation. GO and KEGG enrichment results were visualized according to gene counts and statistical significance values. Visualization of GO enrichment analyses was performed using R software (version 2024.12.1), while KEGG pathway graphs were generated using the ImageGP platform (https://www.bic.ac.cn/BIC/#/, accessed on 6 May 2026) [202,203].

### 4.8. Litmaps References Analysis

**Figure 12:**
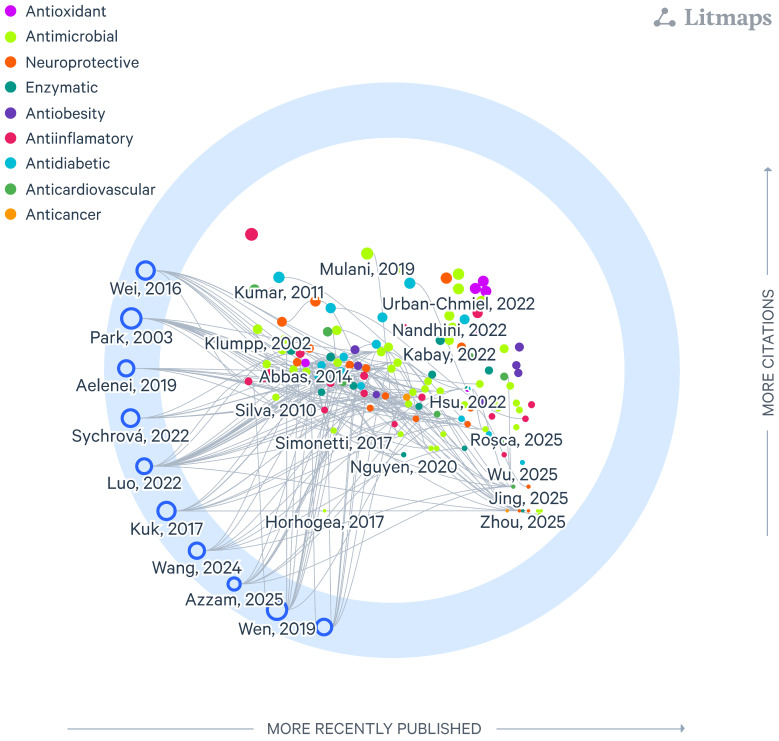
Map of references. It shows the inter-relationships of the articles related to the biological activities of KWG. The articles in the ring are highly correlated with the others.

The literature review was mapped using Litmaps (https://www.litmaps.com). Through Litmaps, forward and backward citation networks were examined through key articles and a visual literature map of related studies was created. This method enabled the systematic identification of related studies on the subject (Figure 12).

## 5. Conclusions

KWG is a naturally occurring isoprenylated flavonoid isolated primarily from the root bark of *M. alba* and other mulberry species. Its methylcyclohexene ring and prenyl groups increase the lipophilicity of the molecule, enhancing its binding affinity to biological targets and cell membranes. Unlike the conventional “one drug–one target” approach, which is often inadequate for treating multifactorial diseases, KWG is a potent bioactive compound with a pleiotropic effect, capable of modulating multiple cellular signaling pathways simultaneously.

Comprehensive studies clearly demonstrate that KWG possesses antimicrobial, antioxidant, anti-inflammatory, anti-diabetic, neuroprotective, cardiovascular protective, anti-obesity, and anticancer properties. The compound highly specifically inhibits key enzymes such as α-glucosidase, PTP1B, PL, BChE, BACE1, and β-GUS in the management of metabolic syndrome, neurodegeneration, and gastrointestinal disorders. At the clinical level, its ability to restore the efficacy of approved antibiotics against formidable pathogens like MRSA and CRKP by creating a synergistic effect, along with its strong antiviral capacity against viruses such as HCoV 229E and DENV, proves its strategic value in infectious diseases. *in silico* and molecular mechanism analyses confirm that the compound exerts its effects by regulating core signaling pathways associated with inflammation, oxidative stress, and cellular proliferation, such as NF-κB, PI3K/AKT/mTOR, Toll-like receptor, and JAK-STAT.

Considering the results of the Structure–Activity Relationship (SAR) and pharmacokinetic studies, KWG exhibits different biological activities derived from its prenyl groups, phenolic hydroxyls, and the methylcyclohexene ring formed via a Diels–Alder reaction. Structural prenyl groups confer a highly lipophilic property on the molecule, facilitating its penetration into cell membranes and the targeted inhibition of specific enzymes. Concurrently, the phenolic hydroxyl and benzopyranyl groups allow the compound to bind to allosteric sites on target proteins through hydrogen bonding. Meanwhile, the methylcyclohexene ring establishes strong hydrophobic interactions that inhibit catalytic residues in enzymes involved in processes such as inflammation and tyrosinase activity. From a pharmacokinetic perspective, KWG has low oral bioavailability and cannot enter the systemic circulation in its original, unchanged form. Instead, it is biotransformed by the gut microbiota into active metabolites that are capable of crossing the blood–brain barrier and exhibit high binding affinities for cellular targets. As KWG exhibits toxic and hemolytic effects in cells at high doses and has limited systemic absorption, therapeutic applications require the use of topical treatments or specifically targeted drug delivery systems. Despite its pharmacological potential, the cytotoxic effects exhibited by KWG at high concentrations require careful evaluation of dosing safety and pharmacokinetic limitations in therapeutic use.

## Figures and Tables

**Table 1:** Morus species containing KWG.

Plant	Part of Plant	References
*Morus alba*	Root barks	[34,35,36,37,39,41,43,44,45,46,47,48,49,50,51,52,53,54,55,56,57,58,59,60,61,62,63,64,65,66,67]
	Root	[68]
	Stem	[69,70]
	Branches	[38,57,59,71,72,73]
	Branch barks	[56]
	Leaves	[59,74]
	Wood	[75]
	Fruits	[59]
	Shoot epidermis	[40]
	Not detected	[76]
*Morus alba* var. *alba*	Leaves and stem bark	[77]
*Morus alba* var. *rosa*		
*Morus rubra*		
*Morus australis*	Stem barks	[78]
	Roots, stems, branches	[79]
*Morus nigra*	Root barks	[80,81]
	Roots and branches	[82]
	Stem barks	[83]
*Morus multicaulis*	Branches	[84,85,86]
*Morus lhou*	Root barks	[87]
*Morus bombycis*	Underground parts	[88]

## Data Availability

All data generated or analyzed as part of this study are available in the article.

## References

[1] Yuan H., Ma Q., Ye L., Piao G. (2016). The traditional medicine and modern medicine from natural products. Molecules.

[2] Atanasov A.G., Zotchev S.B., Dirsch V.M., Orhan I.E., Banach M., Rollinger J.M., Barreca D., Weckwerth W., Bauer R., Bayer E.A. (2021). Natural products in drug discovery: Advances and opportunities. Nat. Rev. Drug Discov..

[3] Elshafie H.S., Camele I., Mohamed A.A. (2023). A comprehensive review on the biological, agricultural and pharmaceutical properties of secondary metabolites based-plant origin. Int. J. Mol. Sci..

[4] Newman D.J., Cragg G.M. (2020). Natural products as sources of new drugs over the nearly four decades from 01/1981 to 09/2019. J. Nat. Prod..

[5] Hopkins A.L. (2008). Network pharmacology: The next paradigm in drug discovery. Nat. Chem. Biol..

[6] Ahmad M., Tahir M., Hong Z., Zia M.A., Rafeeq H., Ahmad M.S., Rehman S.u., Sun J. (2024). Plant and marine-derived natural products: Sustainable pathways for future drug discovery and therapeutic development. Front. Pharmacol..

[7] Tiwari R., Kumar Shukla A., Ritesh M., Tiwari K. (2020). Plant metabolites and their role in health benefits: A brief review. Adv. Pharm. J..

[8] Al-Khayri J.M., Rashmi R., Toppo V., Chole P.B., Banadka A., Sudheer W.N., Nagella P., Shehata W.F., Al-Mssallem M.Q., Alessa F.M. (2023). Plant secondary metabolites: The weapons for biotic stress management. Metabolites.

[9] Dias M.C., Pinto D.C.G.A., Silva A.M.S. (2021). Plant flavonoids: Chemical characteristics and biological activity. Molecules.

[10] Shamsudin N.F., Ahmed Q.U., Mahmood S., Shah S.A.A., Khatib A., Mukhtar S., Alsharif M.A., Parveen H., Zakaria Z.A. (2022). Antibacterial effects of flavonoids and their structure-activity relationship study: A comparative interpretation. Molecules.

[11] Tang S., Wang B., Liu X., Xi W., Yue Y., Tan X., Bai J., Huang L. (2025). Structural insights and biological activities of flavonoids: Implications for novel applications. Food Front..

[12] Kumar S., Pandey A.K., Lu K.P., Sastre J. (2013). Chemistry and biological activities of flavonoids: An overview. Sci. World J..

[13] Shi S., Li J., Zhao X., Liu Q., Song S.J. (2021). A comprehensive review: Biological activity, modification and synthetic methodologies of prenylated flavonoids. Phytochemistry.

[14] Wen L., Zhou T., Jiang Y., Chang S.K., Yang B. (2022). Prenylated flavonoids in foods and their applications on cancer prevention. Crit. Rev. Food Sci. Nutr..

[15] Morante-Carriel J., Živković S., Nájera H., Sellés-Marchart S., Martínez-Márquez A., Martínez-Esteso M.J., Obrebska A., Samper-Herrero A., Bru-Martínez R. (2024). Prenylated flavonoids of the Moraceae family: A comprehensive review of their biological activities. Plants.

[16] Nishi K., Imamura I., Hoashi K., Kiyama R., Mitsuiki S. (2024). Estrogenic prenylated flavonoids in sophora flavescens. Genes.

[17] Dat N.T., Binh P.T.X., Quynh L.T.P., Van Minh C., Huong H.T., Lee J.J. (2010). Cytotoxic prenylated flavonoids from *Morus alba*. Fitoterapia.

[18] Osorio M., Carvajal M., Vergara A., Butassi E., Zacchino S., Mascayano C., Montoya M., Mejías S., Martín M.C.S., Vásquez-Martínez Y. (2021). Prenylated flavonoids with potential antimicrobial activity: Synthesis, biological activity, and *in silico* study. Int. J. Mol. Sci..

[19] Shahinozzaman M., Taira N., Ishii T., Halim M.A., Hossain M.A., Tawata S. (2018). Anti-inflammatory, anti-diabetic, and anti-Alzheimer’s effects of prenylated flavonoids from Okinawa propolis: An investigation by experimental and computational studies. Molecules.

[20] Li D., Fan J., Du L., Ren G. (2024). Prenylated flavonoid fractions from *Glycyrrhiza glabra* alleviate insulin resistance in HepG2 cells by regulating the ERK/IRS-1 and PI3K/Akt signaling pathways. Arch. Pharm. Res..

[21] Seong S.H., Ha M.T., Min B.S., Jung H.A., Choi J.S. (2018). Moracin derivatives from Morus Radix as dual BACE1 and cholinesterase inhibitors with antioxidant and anti-glycation capacities. Life Sci..

[22] Santos C.M.M., Silva A.M.S. (2020). The antioxidant activity of prenylflavonoids. Molecules.

[23] Kong S., Liu Y., Tang R., Liao Q., Bai D., Lv D., Xu Z., Lin L., Li H. (2025). Ultrasound-assisted extraction of prenylated flavonoids from Sophora flavescens: Optimization, mechanistic characterization, antioxidant and anti-inflammatory activities. Ind. Crops Prod..

[24] Tronina T., Bartmańska A., Popłoński J., Rychlicka M., Sordon S., Filip-Psurska B., Milczarek M., Wietrzyk J., Huszcza E. (2023). Prenylated flavonoids with selective toxicity against human cancers. Int. J. Mol. Sci..

[25] Lv H.W., Wang Q.L., Luo M., Zhu M.D., Liang H.M., Li W.J., Cai H., Zhou Z.B., Wang H., Tong S.Q. (2023). Phytochemistry and pharmacology of natural prenylated flavonoids. Arch. Pharm. Res..

[26] R. V.K., Srivastava D., Verma A., Singh V., Kumar U. (2026). Kuwanons from Mulberry (*Morus* spp.): Exploring their structural activity relationship, biosynthesis, and biological properties. Fitoterapia.

[27] Wen P., Hu T.G., Linhardt R.J., Liao S.T., Wu H., Zou Y.X. (2019). Mulberry: A review of bioactive compounds and advanced processing technology. Trends Food Sci. Technol..

[28] Nomura T., Hano Y. (1999). Chemistry, biosynthesis, and biological activity of natural diels-alder type adducts from moraceous plants. Basic Life Sci..

[29] Tortora C., Pisano L., Vergine V., Ghirga F., Iazzetti A., Calcaterra A., Marković V., Botta B., Quaglio D. (2022). Synthesis, biosynthesis, and biological activity of Diels–Alder adducts from *Morus* genus: An Update. Molecules.

[30] Chan E.W.C., Lye P.Y., Wong S.K. (2016). Phytochemistry, pharmacology, and clinical trials of *Morus alba*. Chin. J. Nat. Med..

[31] Yang Y., Tan Y.X., Chen R.Y., Kang J. (2014). The latest review on the polyphenols and their bioactivities of Chinese *Morus* plants. J. Asian Nat. Prod. Res..

[32] Luo S.Y., Zhu J.Y., Zou M.F., Yin S., Tang G.H. (2022). Mulberry Diels–Alder-type adducts: Isolation, structure, bioactivity, and synthesis. Nat. Prod. Bioprospect..

[33] Li N., Lu W., Ren H., Chen Z. (2024). Chemistry, bioactivities, structure–activity relationship, biosynthesis and metabolism of prenylated flavonoids in Moraceae plants. Food Funct..

[34] Nomura T., Fukai T. (1980). Kuwanon G, a new flavone derivative from the root barks of the cultivated mulberry tree (*Morus alba* L.). Chem. Pharm. Bull..

[35] Park K.M., You J.S., Lee H.Y., Baek N.I., Hwang J.K. (2003). Kuwanon G: An antibacterial agent from the root bark of *Morus alba* against oral pathogens. J. Ethnopharmacol..

[36] Jung H.W., Kang S.Y., Kang J.S., Kim A.R., Woo E.R., Park Y.K. (2014). Effect of kuwanon G isolated from the root bark of *Morus alba* on ovalbumin-induced allergic response in a mouse model of asthma. Phytother. Res..

[37] Lee H.J., Ryu J., Park S.H., Woo E.R., Kim A.R., Lee S.K., Kim Y.S., Kim J.O., Hong J.H., Lee C.J. (2014). Effects of *Morus alba* L. and natural products including morusin on *in vivo* secretion and *in vitro* production of airway MUC5AC mucin. Tuberc. Respir. Dis..

[38] Kwon R.H., Thaku N., Timalsina B., Park S.E., Choi J.S., Jung H.A. (2022). Inhibition mechanism of components isolated from *Morus alba* branches on diabetes and diabetic complications via experimental and molecular docking analyses. Antioxidants.

[39] Kuk E.B., Jo A.R., Oh S.I., Sohn H.S., Seong S.H., Roy A., Choi J.S., Jung H.A. (2017). Anti-Alzheimer’s disease activity of compounds from the root bark of *Morus alba* L.. Arch. Pharm. Res..

[40] Takasugi M., Ishikawa S., Nagao S., Masamune T., Shirata A., Takahashi K. (1980). Albanins F and G, natural Diels-Alder adducts from mulberry. Chem. Lett..

[41] Oshima Y., Konno C., Hikino H., Matsushita K. (1980). Structure of moracenin B, a hypotensive principle of *Morus* root barks. Tetrahedron Lett..

[42] Nomura T., Fukai T., Narita T., Terada S., Uzawa J., Iitaka Y., Takasugi M., Ishikawa S.i., Nagao S., Masamune T. (1981). Confirmation of the structures of kuwanons G and H (albanins F and G) by partial synthesis. Tetrahedron Lett..

[43] Wu S.C., Han F., Song M.R., Chen S., Li Q., Zhang Q., Zhu K., Shen J.Z. (2019). Natural flavones from *Morus alba* against methicillin-resistant *Staphylococcus aureus* via targeting the proton motive force and membrane permeability. J. Agric. Food Chem..

[44] Chang Y.S., Jin H.G., Lee H., Lee D.S., Woo E.R. (2019). Phytochemical constituents of the root bark from *Morus alba* and their IL-6 inhibitory activity. Nat. Prod. Sci..

[45] Jin S.E., Ha H., Shin H.K., Seo C.S. (2019). Anti-allergic and anti-inflammatory effects of kuwanon G and morusin on MC/9 mast cells and HaCaT keratinocytes. Molecules.

[46] Paudel P., Park S.E., Seong S.H., Jung H.A., Choi J.S. (2019). Novel diels–alder type adducts from *Morus alba* root bark targeting human monoamine oxidase and dopaminergic receptors for the management of neurodegenerative diseases. Int. J. Mol. Sci..

[47] Liang J.H., Fu Y.W., Zhang Q.Z., Xu D.H., Wang B., Lin D.J. (2015). Identification and effect of two flavonoids from root bark of *Morus alba* against *Ichthyophthirius multifiliis* in Grass Carp. J. Agric. Food Chem..

[48] Čulenová M., Sychrová A., Hassan S.T.S., Berchová-Bímová K., Svobodová P., Helclová A., Michnová H., Hošek J., Vasilev H., Suchý P. (2020). Multiple *in vitro* biological effects of phenolic compounds from *Morus alba* root bark. J. Ethnopharmacol..

[49] Jing W., Yan R., Wang Y. (2015). A practical strategy for chemical profiling of herbal medicines using ultra-high performance liquid chromatography coupled with hybrid triple quadrupole-linear ion trap mass spectrometry: A case study of Mori Cortex. Anal. Methods.

[50] Kim K.T., Shin M.C., Kim H.H., Cho C.W., Lee W.J., Woo E.R., Kim K.H., Kang J.S. (2015). Specification and analysis of multiple marker compounds for quality control of Mori Cortex Radicis by HPLC. Bull. Korean Chem. Soc..

[51] Seo C.S., Shin H.K. (2018). Simultaneous quantification of two flavonoids in *Morus alba* by high performance liquid chromatography coupled with photodiode array detector. Nat. Prod. Commun..

[52] Xia C.L., Tang G.H., Guo Y.Q., Xu Y.K., Huang Z.S., Yin S. (2019). Mulberry Diels-Alder-type adducts from *Morus alba* as multi-targeted agents for Alzheimer’s disease. Phytochemistry.

[53] Zhao Y., Kongstad K.T., Jäger A.K., Nielsen J., Staerk D. (2018). Quadruple high-resolution α-glucosidase/α-amylase/PTP1B/radical scavenging profiling combined with high-performance liquid chromatography–high-resolution mass spectrometry–solid-phase extraction–nuclear magnetic resonance spectroscopy for identification of antidiabetic constituents in crude root bark of *Morus alba* L.. J. Chromatogr. A.

[54] Zhao X., Qiu Z., Ma Z., Liu Y., Ren X., Yu X., Sun L., Wang M. (2022). Comprehensive quality evaluation of the root bark of *Morus alba* L. based on high-performance liquid chromatography fingerprinting and chemometric analyses. Chem. Biodivers..

[55] Zhu M., Wang Z.J., He Y.J., Qin Y., Zhou Y., Qi Z.H., Zhou Z.S., Zhu Y.Y., Jin D.N., Chen S.S. (2021). Bioguided isolation, identification and bioactivity evaluation of anti-MRSA constituents from *Morus alba* Linn. J. Ethnopharmacol..

[56] Li W., Chen L., Li M., Peng K., Lin X., Feng Y., Zou Y., Wu X. (2025). Study on chemical composition, anti-inflammatory activity and quality control of the branch bark of *Morus alba* L.. Fitoterapia.

[57] Lee J.H., Kim H.W., Kim S.A., Ju W.T., Kim S.R., Kim H.B., Cha I.S., Kim S.W., Park J.W., Kang S.K. (2024). Modulatory effects of the Kuwanon-rich fraction from mulberry root bark on the renin–angiotensin system. Foods.

[58] Paudel P., Yu T., Seong S.H., Kuk E.B., Jung H.A., Choi J.S. (2018). Protein tyrosine phosphatase 1B inhibition and glucose uptake potentials of mulberrofuran G, albanol B, and kuwanon G from root bark of *Morus alba* L. in insulin-resistant HepG2 cells: An *in vitro* and *in silico* study. Int. J. Mol. Sci..

[59] Chen Z., Du X., Yang Y., Cui X., Zhang Z., Li Y. (2018). Comparative study of chemical composition and active components against α-glucosidase of various medicinal parts of *Morus alba* L.. Biomed. Chromatogr..

[60] Hsu J.H., Yang C.S., Chen J.J. (2022). Antioxidant, anti-α-glucosidase, antityrosinase, and anti-inflammatory activities of bioactive components from *Morus alba*. Antioxidants.

[61] Lim H.J., Jin H.G., Woo E.R., Lee S.K., Kim H.P. (2013). The root barks of *Morus alba* and the flavonoid constituents inhibit airway inflammation. J. Ethnopharmacol..

[62] Kimura Y., Okuda H., Nomura T., Fukai T., Arichi S. (1986). Effects of phenolic constituents from the mulberry tree on arachidonate metabolism in rat platelets. J. Nat. Prod..

[63] You S., Kim G.H. (2019). Protective effect of Mori Cortex radicis extract against high glucose-induced oxidative stress in PC12 cells. Biosci. Biotechnol. Biochem..

[64] Yimam M., Jiao P., Hong M., Brownell L., Lee Y.C., Kim H.J., Nam J.B., Kim M.R., Jia Q. (2019). *Morus alba*, a medicinal plant for appetite suppression and weight loss. J. Med. Food.

[65] Zhu L., Wang Y., Zhang X., Ma X., Yan F., Feng L., Ma T. (2026). Fluorescence-based high-throughput screening of carbapenemase inhibitors to enhance the antibacterial efficacy of antibiotics against carbapenem-resistant *Klebsiella pneumoniae*. Sens. Actuators B Chem..

[66] Koirala P., Seong S.H., Zhou Y., Shrestha S., Jung H.A., Choi J.S. (2018). Structure–activity relationship of the tyrosinase inhibitors kuwanon G, mulberrofuran G, and albanol B from *Morus* species: A kinetics and molecular docking study. Molecules.

[67] Liu Y.J., Li S.Y., Hou J., Liu Y.F., Wang D.D., Jiang Y.S., Ge G.B., Liang X.M., Yang L. (2016). Identification and characterization of naturally occurring inhibitors against human carboxylesterase 2 in white mulberry root-bark. Fitoterapia.

[68] Baek S.H., Hwang S., Park T., Kwon Y.J., Cho M., Park D. (2021). Evaluation of selective cox-2 inhibition and *in silico* study of Kuwanon derivatives isolated from *Morus alba*. Int. J. Mol. Sci..

[69] Nguyen Thu H., Hoang Thi My L., Nguyen Van P., Do Thi H. (2020). Bioactivity-guided isolation and identification of xanthine oxidase inhibitors from *Morus alba* bark. J. Adv. Pharm. Res..

[70] Phong N.V., Lee Y.M., Min B.S., Kim J.A. (2024). Development and validation of an HPLC-DAD method for simultaneous quantitation of steppogenin and flavonoids from the stems of *Morus alba*. Nat. Prod. Sci..

[71] Nguyệt B.T.M., Khanh T.H.N. (2016). The isolated compounds from the twigs of *Morus alba* L. in dong thap. Vietnam J. Sci. Technol..

[72] Jin Z., Xiang W., Shi X., Jiang F., Jia Y., Zhang Y., Zeng L., Huang X., Xu L. (2025). Identification of compounds against atherosclerosis induced by ox-LDL based on cell extraction/UPLC–MS/MS from mulberry twigs and their mechanistic analysis. Chem. Biol. Technol. Agric..

[73] Zhou Q., Tong Y.P., Wan J., Yan L.H., Jiang C.X., Hu J.F. (2025). Mulberry Diels-Alder-type adducts and other phenolic compounds with ATP-citrate lyase inhibitory effects from *Morus alba* and their chemotaxonomic significance. Biochem. Syst. Ecol..

[74] Sun F., Shen L., Ma Z. (2011). Screening for ligands of human aromatase from mulberry (*Mori alba* L.) leaf by using high-performance liquid chromatography/tandem mass spectrometry. Food Chem..

[75] Chaita E., Lambrinidis G., Cheimonidi C., Agalou A., Beis D., Trougakos I., Mikros E., Skaltsounis A.L., Aligiannis N., Ferreira I.C.F.R. (2017). Anti-melanogenic properties of Greek plants. A novel depigmenting agent from *Morus alba* wood. Molecules.

[76] Liu H.R., Liu Y.M., Hou T.L., Li C.T., Zhang Q.Z. (2021). Antiparasitic efficacy of crude plant extracts and compounds purified from plants against the Fish Monogenean *Neobenedenia girellae*. J. Aquat. Anim. Health.

[77] Thabti I., Albert Q., Philippot S., Dupire F., Westerhuis B., Fontanay S., Risler A., Kassab T., Elfalleh W., Aferchichi A. (2020). Advances on antiviral activity of *Morus* spp. plant extracts: Human coronavirus and virus-related respiratory tract infections in the spotlight. Molecules.

[78] Zhang Q.J., Tang Y.B., Chen R.Y., Yu D.Q. (2007). Three new cytotoxic Diels-Alder-type adducts from *Morus australis*. Chem. Biodivers..

[79] Zheng Z.P., Tan H.Y., Wang M. (2012). Tyrosinase inhibition constituents from the roots of *Morus australis*. Fitoterapia.

[80] Mascarello A., Orbem Menegatti A.C., Calcaterra A., Martins P.G.A., Chiaradia-Delatorre L.D., D’Acquarica I., Ferrari F., Pau V., Sanna A., De Logu A. (2018). Naturally occurring Diels-Alder-type adducts from *Morus nigra* as potent inhibitors of *Mycobacterium tuberculosis* protein tyrosine phosphatase B. Eur. J. Med. Chem..

[81] Simonetti G., Brasili E., D’Auria F.D., Corpolongo S., Ferrari F., Pasqua G., Valletta A. (2017). Prenylated flavonoids and total extracts from *Morus nigra* L. root bark inhibit *in vitro* growth of plant pathogenic fungi. Plant Biosyst.—Int. J. Deal. All Asp. Plant Biol..

[82] Zheng Z.P., Cheng K.W., Zhu Q., Wang X.C., Lin Z.X., Wang M. (2010). Tyrosinase inhibitory constituents from the roots of *Morus nigra*: A structure−activity relationship study. J. Agric. Food Chem..

[83] Abbas G.M., Abdel Bar F.M., Baraka H.N., Gohar A.A., Lahloub M.F. (2014). A new antioxidant stilbene and other constituents from the stem bark of *Morus nigra* L.. Nat. Prod. Res..

[84] Feng F., Xiang W., Gao H., Jia Y., Zhang Y., Zeng L., Chen J., Huang X., Xu L. (2022). Rapid screening of nonalkaloid α-glucosidase inhibitors from a Mulberry Twig extract using enzyme-functionalized magnetic nanoparticles coupled with UPLC-MS/MS. J. Agric. Food Chem..

[85] Zhu Y., Xiang W., Shen Y., Jia Y., Zhang Y., Zeng L., Chen J., Zhou Y., Xue X., Huang X. (2022). New butyrylcholinesterase inhibitor derived from mulberry twigs, a kind of agricultural byproducts. Ind. Crops Prod..

[86] Xiang W., Xia Z., Xu L. (2021). UPLC-MS/MS Profiling, antioxidant, α-glucosidase inhibitory, cholinesterase inhibitory, and cardiovascular protection potentials of Jialing 20 (*Morus multicaulis* Perr.) mulberry branch extract. Foods.

[87] Hano Y., Hirakura K., Nomura T., Terada S., Fukushima K. (1984). Components of root bark of *Morus lhou* -1. Structures of two new natural Diels-Alder Adducts, kuwanons N and O. Planta Med..

[88] Mihara S.I., Hara M., Nakamura M., Sakurawi K., Tokura K., Fujimoto M., Fukai T., Nomura T. (1995). Non-peptide bombesin receptor antagonists, kuwanon G and H, isolated from mulberry. Biochem. Biophys. Res. Commun..

[89] Nomura T. (1988). Phenolic Compounds of the Mulberry Tree and Related Plants. Fortschritte Chem. Org. Naturstoffe.

[90] Nomura T., Hano Y., Ueda S. (1995). Chemistry and Biosynthesis of Natural Diels-Alder Type Adducts from Moraceous Plants. Stud. Nat. Prod. Chem..

[91] Nomura T., Hano Y., Fukai T. (2009). Chemistry and biosynthesis of isoprenylated flavonoids from Japanese mulberry tree. Proc. Jpn. Acad. Ser. B.

[92] Takayama M., Nomura T., Nojima K. (1995). Structure of the diene originating from a retro-Diels–Alder cleavage, of the natural product kuwanon g. Rapid Commun. Mass Spectrom..

[93] Luo S.Y., Tang Z.Y., Li Q., Weng J., Yin S., Tang G.H. (2021). Total synthesis of mulberry Diels–Alder-type adducts kuwanons G and H. J. Org. Chem..

[94] Martyniak A., Tomasik P.J. (2022). A new perspective on the renin-angiotensin system. Diagnostics.

[95] Unger T. (2002). The role of the renin-angiotensin system in the development of cardiovascular disease. Am. J. Cardiol..

[96] Cochain C., Zernecke A. (2017). Macrophages in vascular inflammation and atherosclerosis. Pflug. Arch..

[97] Munno M., Mallia A., Greco A., Modafferi G., Banfi C., Eligini S. (2024). Radical oxygen species, oxidized low-density lipoproteins, and lectin-like oxidized low-density lipoprotein receptor 1: A vicious circle in atherosclerotic process. Antioxidants.

[98] Liu X.X., Zhang X.W., Wang K., Wang X.Y., Ma W.L., Cao W., Mo D., Sun Y., Li X.Q. (2018). Kuwanon G attenuates atherosclerosis by upregulation of LXRα-ABCA1/ABCG1 and inhibition of NFκB activity in macrophages. Toxicol. Appl. Pharmacol..

[99] Banday M.Z., Sameer A.S., Nissar S. (2020). Pathophysiology of diabetes: An overview. Avicenna J. Med..

[100] Kashtoh H., Baek K.H. (2022). Recent updates on phytoconstituent alpha-glucosidase inhibitors: An approach towards the treatment of type two diabetes. Plants.

[101] Kumar S., Narwal S., Kumar V., Prakash O. (2011). α-glucosidase inhibitors from plants: A natural approach to treat diabetes. Pharmacogn. Rev..

[102] Thiebaut P.A., Besnier M., Gomez E., Richard V. (2016). Role of protein tyrosine phosphatase 1B in cardiovascular diseases. J. Mol. Cell. Cardiol..

[103] Abdelsalam S.S., Korashy H.M., Zeidan A., Agouni A. (2019). The role of protein tyrosine phosphatase (PTP)-1B in cardiovascular disease and its interplay with insulin resistance. Biomolecules.

[104] Wu E., Zhu Y., Wei Q., Lu H., Zou Y., Liu F., Li Q. (2025). Inhibition mechanism of mulberry prenylated flavonoids sanggenone D/kuwanon G against α-glucosidase and the regulation of glucose via GLUT4 pathway. Nutrients.

[105] Gomes J.M.G., Costa J.d.A., Alfenas R.d.C.G. (2017). Metabolic endotoxemia and diabetes mellitus: A systematic review. Metabolism.

[106] Fuke N., Nagata N., Suganuma H., Ota T. (2019). Regulation of gut microbiota and metabolic endotoxemia with dietary factors. Nutrients.

[107] Liu C., Zeng H., Jiang R., Wang K., Ouyang J., Wen S., Peng L., Xu H., Huang J., Liu Z. (2023). Effects of mulberry leaf Fu Tea on the intestines and intestinal flora of Goto-Kakizaki type 2 diabetic rats. Foods.

[108] Guo H., Xu Y., Huang W., Zhou H., Zheng Z., Zhao Y., He B., Zhu T., Tang S., Zhu Q. (2016). Kuwanon G preserves LPS-induced disruption of gut epithelial barrier *in vitro*. Molecules.

[109] Moldoveanu B., Otmishi P., Jani P., Walker J., Sarmiento X., Guardiola J., Saad M., Yu J. (2008). Inflammatory mechanisms in the lung. J. Inflamm. Res..

[110] Bezerra F.S., Lanzetti M., Nesi R.T., Nagato A.C., Silva C.P.e., Kennedy-Feitosa E., Melo A.C., Cattani-Cavalieri I., Porto L.C., Valenca S.S. (2023). Oxidative stress and inflammation in acute and chronic lung injuries. Antioxidants.

[111] Fan F., Guo R., Pan K., Xu H., Chu X. (2025). Mucus and mucin: Changes in the mucus barrier in disease states. Tissue Barriers.

[112] Lee J.W., Kim E.N., Jeong G.S. (2024). Anti-inflammatory herbal extracts and their drug discovery perspective in atopic dermatitis. Biomol. Ther..

[113] Peng Y., Yang M., Wen J., Chen H., Shen W., Jiang L., Li Y., Lin L., Du Z. (2024). Advancements in the application of natural extracts for atopic dermatitis treatment. J. Dermatol. Sci. Cosmet. Technol..

[114] DuBois R.N., Abramson S.B., Crofford L., Gupta R.A., Simon L.S., van de Putte L.B.A., Lipsky P.E. (1998). Cyclooxygenase in biology and disease. FASEB J..

[115] Rouzer C.A., Marnett L.J. (2020). Structural and chemical biology of the interaction of cyclooxygenase with substrates and non-steroidal anti-inflammatory drugs. Chem. Rev..

[116] Rucker D., Dhamoon A.S. (2022). Physiology, Thromboxane A2. StatPearls.

[117] Kuhn H., Chaitidis P., Roffeis J., Walther M. (2007). Arachidonic acid metabolites in the cardiovascular system: The role of lipoxygenase isoforms in atherogenesis with particular emphasis on vascular remodeling. J. Cardiovasc. Pharmacol..

[118] Paes A.M.d.A., Gaspar R.S., Fuentes E., Wehinger S., Palomo I., Trostchansky A. (2019). Lipid metabolism and signaling in platelet function. Adv. Exp. Med. Biol..

[119] Nakai D., Miyake M. (2023). Intestinal membrane function in inflammatory bowel disease. Pharmaceutics.

[120] Buyse M., Radeva G., Bado A., Farinotti R. (2005). Intestinal inflammation induces adaptation of P-glycoprotein expression and activity. Biochem. Pharmacol..

[121] Jing W., Gao X., Han B., Wei B., Hu N., Li S., Yan R., Wang Y. (2017). Mori Cortex regulates P-glycoprotein in Caco-2 cells and colons from rats with experimental colitis via direct and gut microbiota-mediated mechanisms. RSC Adv..

[122] Sahu P., Satapathy T. (2025). Immunopharmacology of senescence: Targeting the senescence-associated secretory phenotype (SASP)—A mechanism-based review. Inflammopharmacology.

[123] Lim H., Park B.K., Shin S.Y., Kwon Y.S., Kim H.P. (2017). Methyl caffeate and some plant constituents inhibit age-related inflammation: Effects on senescence-associated secretory phenotype (SASP) formation. Arch. Pharm. Res..

[124] Chen Z.R., Huang J.B., Yang S.L., Hong F.F. (2022). Role of cholinergic signaling in Alzheimer’s disease. Molecules.

[125] Sharifi-Rad J., Rapposelli S., Sestito S., Herrera-Bravo J., Arancibia-Diaz A., Salazar L.A., Yeskaliyeva B., Beyatli A., Leyva-Gómez G., González-Contreras C. (2022). Multi-target mechanisms of phytochemicals in Alzheimer’s disease: Effects on oxidative Stress, neuroinflammation and protein aggregation. J. Pers. Med..

[126] Moreira N.C.D.S., Lima J.E.B.d.F., Marchiori M.F., Carvalho I., Sakamoto-Hojo E.T. (2022). Neuroprotective effects of cholinesterase inhibitors: Current scenario in therapies for Alzheimer’s disease and future perspectives. J. Alzheimers Dis. Rep..

[127] Jing Y., Yao P., Zhu H., Yu L., Lin Y., Kang D. (2025). The role and mechanisms of G protein-coupled receptors in Parkinson’s disease. Neurol. Sci..

[128] Jones-Tabah J. (2023). Targeting G protein-coupled receptors in the treatment of Parkinson’s disease. J. Mol. Biol..

[129] Finberg J.P.M., Rabey J.M. (2016). Inhibitors of MAO-A and MAO-B in psychiatry and neurology. Front. Pharmacol..

[130] Riederer P., Laux G. (2011). MAO-inhibitors in Parkinson’s disease. Exp. Neurobiol..

[131] Kumar B., Sheetal, Mantha A.K., Kumar V. (2016). Recent developments on the structure–activity relationship studies of MAO inhibitors and their role in different neurological disorders. RSC Adv..

[132] Chen R., Shi J., Yin Q., Li X., Sheng Y., Han J., Zhuang P., Zhang Y. (2018). Morphological and pathological characteristics of brain in diabetic encephalopathy. J. Alzheimer’s Dis..

[133] Liu J., Wang S., Feng L., Ma D., Fu Q., Song Y., Jia X., Ma S. (2013). Hypoglycemic and antioxidant activities of paeonol and its beneficial effect on diabetic encephalopathy in streptozotocin-induced diabetic rats. J. Med. Food.

[134] Gan W.-J., Gao C.-L., Zhang W.-Q., Gu J.-L., Zhao T.-T., Guo H.-L., Zhou H., Xu Y., Yu L.-L., Li L.-F. (2021). Kuwanon G protects HT22 cells from advanced glycation end product-induced damage. Exp. Ther. Med..

[135] Zhang Y., Niu H., Zhang X., Xie W., Zhang K., Zhang L., Jin Y. (2025). Elucidating the mechanism of Kuwanon G in treating diabetic encephalopathy through network pharmacology: A comprehensive study. J. Chin. Pharm. Sci..

[136] Zhang Y., Zhang S., Niu H., Xie W., Tan Y., Li D., Jin Y. (2025). Metabolite-based network pharmacology, molecular docking, and dynamics simulations to preliminarily verify treating diabetic encephalopathy effect of kuwanon G. Food Sci. Nutr..

[137] Blüher M. (2025). An overview of obesity-related complications: The epidemiological evidence linking body weight and other markers of obesity to adverse health outcomes. Diabetes Obes. Metab..

[138] Janić M., Janež A., El-Tanani M., Rizzo M. (2025). Obesity: Recent advances and future perspectives. Biomedicines.

[139] Subramaniyan V., Hanim Y.U. (2025). Role of pancreatic lipase inhibition in obesity treatment: Mechanisms and challenges towards current insights and future directions. Int. J. Obes..

[140] Hou X.D., Ge G.B., Weng Z.M., Dai Z.R., Leng Y.H., Ding L.L., Jin L.L., Yu Y., Cao Y.F., Hou J. (2018). Natural constituents from Cortex Mori Radicis as new pancreatic lipase inhibitors. Bioorg. Chem..

[141] Vasincu A., Rusu R.N., Ababei D.C., Neamțu M., Arcan O.D., Macadan I., Beșchea Chiriac S., Bild W., Bild V. (2023). Exploring the therapeutic potential of cannabinoid receptor antagonists in inflammation, diabetes mellitus and obesity. Biomedicines.

[142] Rossi F., Punzo F., Umano G.R., Argenziano M., Miraglia Del Giudice E. (2018). Role of cannabinoids in obesity. Int. J. Mol. Sci..

[143] Murray P.E., Coffman J.A., Garcia-Godoy F. (2024). Oral pathogens’ substantial burden on cancer, cardiovascular diseases, Alzheimer’s, diabetes, and other systemic diseases: A public health crisis—A comprehensive review. Pathogens.

[144] Prosper A., Desnot L., Lê S., Minty M., Thomas C., Marty M., Blasco-Baque V., Canceill T., Prosper A., Desnot L. (2026). Oral and systemic diseases: Critical relationships between human health, tooth decay, periodontal diseases and multidisciplinary care. Br. J. Hosp. Med..

[145] Oetiker N., Salinas D., Lucero-Mora J., Orellana R., Quiroz-Muñoz M., Bravo D., Pérez-Donoso J.M. (2024). Antimicrobial effect of copper nanoparticles on relevant supragingival oral bacteria. Microorganisms.

[146] Wang W., Tao R., Tong Z., Ding Y., Kuang R., Zhai S., Liu J., Ni L. (2012). Effect of a novel antimicrobial peptide chrysophsin-1 on oral pathogens and *Streptococcus mutans* biofilms. Peptides.

[147] Chiaradia L.D., Martins P.G.A., Cordeiro M.N.S., Guido R.V.C., Ecco G., Andricopulo A.D., Yunes R.A., Vernal J., Nunes R.J., Terenzi H. (2011). Synthesis, biological evaluation and molecular modeling of chalcone derivatives as potent inhibitors of *Mycobacterium tuberculosis* protein tyrosine phosphatases (PtpA and PtpB). J. Med. Chem..

[148] Dada N.Y., Shah M.Z., Sacher S., Govindh M.P., Bhedsurkar P., Nair R.R., Kizhakethil R., Barage S., Ray A., Guldhe A. (2026). Molecular insights into anabaenopeptin-mediated inhibition of protein tyrosine phosphatase B in the *Mycobacterium tuberculosis* complex. ACS Omega.

[149] Ruddraraju K.V., Aggarwal D., Zhang Z.Y. (2020). Therapeutic targeting of protein tyrosine phosphatases from *Mycobacterium tuberculosis*. Microorganisms.

[150] Silva A.P., Tabernero L. (2010). New strategies in fighting TB: Targeting *Mycobacterium tuberculosis*-secreted phosphatases MptpA & MptpB. Future Med. Chem..

[151] Urban-Chmiel R., Marek A., Stępień-Pyśniak D., Wieczorek K., Dec M., Nowaczek A., Osek J. (2022). Antibiotic resistance in bacteria—A review. Antibiotics.

[152] Chinemerem Nwobodo D., Ugwu M.C., Oliseloke Anie C., Al-Ouqaili M.T.S., Chinedu Ikem J., Victor Chigozie U., Saki M. (2022). Antibiotic resistance: The challenges and some emerging strategies for tackling a global menace. J. Clin. Lab. Anal..

[153] Nandhini P., Kumar P., Mickymaray S., Alothaim A.S., Somasundaram J., Rajan M. (2022). Recent developments in methicillin-resistant *Staphylococcus aureus* (MRSA) treatment: A review. Antibiotics.

[154] Mulani M.S., Kamble E.E., Kumkar S.N., Tawre M.S., Pardesi K.R. (2019). Emerging strategies to combat ESKAPE pathogens in the era of antimicrobial resistance: A review. Front. Microbiol..

[155] Horhogea C., Rîmbu C., Aelenei P., Guguianu E., Crețu C., Dimitriu G., Miron A. (2017). Preliminary studies regarding antimicrobial effect of various kuwanon G-antibiotic combinations on some MRSA strains. Lucr. Ştiinţifice. Ser. Med. Vet. Iasi.

[156] Aelenei P., Horhogea C.E., Rîmbu C.M., Dimitriu G.T.P., Aprotosoaie A.C., Miron A. (2019). Pharmacy morusin and kuwanon G-promising anti-MRSA agents. Med.-Surg. J..

[157] Aelenei P., Rimbu C.M., Horhogea C.E., Lobiuc A., Neagu A.N., Dunca S.I., Motrescu I., Dimitriu G., Aprotosoaie A.C., Miron A. (2020). Prenylated phenolics as promising candidates for combination antibacterial therapy: Morusin and kuwanon G. Saudi Pharm. J..

[158] Fihn C.A., Carlson E.E. (2021). Targeting a highly conserved domain in bacterial histidine kinases to generate inhibitors with broad spectrum activity. Curr. Opin. Microbiol..

[159] Klumpp S., Krieglstein J. (2002). Phosphorylation and dephosphorylation of histidine residues in proteins. Eur. J. Biochem..

[160] Gilmour R., Foster J.E., Sheng Q., McClain J.R., Riley A., Sun P.M., Ng W.L., Yan D., Nicas T.I., Henry K. (2005). New class of competitive inhibitor of bacterial histidine kinases. J. Bacteriol..

[161] Barker W.T., Jania L.A., Melander R.J., Koller B.H., Melander C. (2020). Eukaryotic phosphatase inhibitors enhance colistin efficacy in gram-negative bacteria. Chem. Biol. Drug Des..

[162] Liu P., Li X., Luo M., Xu X., Su K., Chen S., Qing Y., Li Y., Qiu J. (2018). Risk factors for carbapenem-resistant *Klebsiella pneumoniae* infection: A meta-analysis. Microb. Drug Resist..

[163] Karampatakis T., Tsergouli K., Behzadi P. (2023). Carbapenem-resistant *Klebsiella pneumoniae*: Virulence factors, molecular epidemiology and latest updates in treatment options. Antibiotics.

[164] Zhu W.M., Yuan Z., Zhou H.Y. (2020). Risk factors for carbapenem-resistant *Klebsiella pneumoniae* infection relative to two types of control patients: A systematic review and meta-analysis. Antimicrob. Resist. Infect. Control.

[165] Sharma R., Bhattu M., Tripathi A., Verma M., Acevedo R., Kumar P., Rajput V.D., Singh J. (2023). Potential medicinal plants to combat viral infections: A way forward to environmental biotechnology. Environ. Res..

[166] Kvansakul M. (2017). Viral infection and apoptosis. Viruses.

[167] Kabay G., DeCastro J., Altay A., Smith K., Lu H.W., Capossela A.M.D., Moarefian M., Aran K., Dincer C. (2022). Emerging biosensing technologies for the diagnostics of viral infectious diseases. Adv. Mater..

[168] He M., He C.Q., Ding N.Z. (2025). Human viruses: An ever-increasing list. Virology.

[169] Ciotti M., Ciccozzi M., Terrinoni A., Jiang W.C., Wang C.B., Bernardini S. (2020). The COVID-19 pandemic. Crit. Rev. Clin. Lab. Sci..

[170] Salahshoori I., Mobaraki-Asl N., Seyfaee A., Mirzaei Nasirabad N., Dehghan Z., Faraji M., Ganjkhani M., Babapoor A., Shadmehr S.Z., Hamrang A. (2021). Overview of COVID-19 disease: Virology, epidemiology, prevention diagnosis, treatment, and vaccines. Biologics.

[171] Sopjani M., Falco F., Impellitteri F., Guarrasi V., Nguyen Thi X., Dërmaku-Sopjani M., Faggio C. (2024). Flavonoids derived from medicinal plants as a COVID-19 treatment. Phytother. Res..

[172] Vairaperumal T., Lee P.T., Liu P.Y. (2025). Portable point-of-care diagnosis platforms and emerging predictive biomarkers for rapid detection of severe dengue viral infection. ACS Sens..

[173] Rosca E.C., Garg D., Perez-Lloret S., Mohamed Ibrahim N., Phokaewvarangkul O., Sringean J., Holla V., Yadav R., Desai S., Pal P.K. (2025). Movement disorders after dengue virus infection: A scoping review. Mov. Disord..

[174] Wan S.W., Lee Y.R., Ho T.S., Chang C.P. (2022). Regulation of innate immune signaling pathways by autophagy in dengue virus infection. IUBMB Life.

[175] Limthongkul J., Akkarasereenon K., Yodweerapong T., Songthammawat P., Tong-Ngam P., Tubsuwan A., Kunkaew N., Kanjanasirirat P., Khumpanied T., Wannalo W. (2023). Novel potent autophagy inhibitor Ka-003 inhibits dengue virus replication. Viruses.

[176] Carlson K.B., Dilley A., O’Grady T., Johnson J.A., Lopman B., Viscidi E. (2024). A narrative review of norovirus epidemiology, biology, and challenges to vaccine development. Vaccines.

[177] Su X., D’Souza D.H. (2013). Naturally occurring flavonoids against human norovirus surrogates. Food Environ. Virol..

[178] Lim C.Y., Kim H., Chung M.S. (2021). Mori Cortex Radicis extract inhibits human norovirus surrogate in simulated digestive conditions. Food Sci. Biotechnol..

[179] Bula B., Etana M., Abdisa T., Getu M. (2023). Epidemiology of helminthes, protozoans and ectoparasites of fishes: A review. J. Vet. Med. Anim. Sci..

[180] Huang K., Hu G., Wang R., Zeng Q., Li W., Zou H., Wu S., Wang G., Li M. (2022). *In vitro* assessment of berberine against *Ichthyophthirius multifiliis* in Goldfish. Pathogens.

[181] Palmieri D., Ianiri G., Del Grosso C., Barone G., De Curtis F., Castoria R., Lima G. (2022). Advances and perspectives in the use of biocontrol agents against fungal plant diseases. Horticulturae.

[182] Olufunmilayo E.O., Gerke-Duncan M.B., Holsinger R.M.D. (2023). Oxidative stress and antioxidants in neurodegenerative disorders. Antioxidants.

[183] Kıran T.R., Otlu O., Karabulut A.B. (2023). Oxidative stress and antioxidants in health and disease. J. Lab. Med..

[184] Halliwell B. (2023). Understanding mechanisms of antioxidant action in health and disease. Nat. Rev. Mol. Cell Biol..

[185] Asma S.T., Acaroz U., Imre K., Morar A., Shah S.R.A., Hussain S.Z., Arslan-Acaroz D., Demirbas H., Hajrulai-Musliu Z., Istanbullugil F.R. (2022). Natural products/bioactive compounds as a source of anticancer drugs. Cancers.

[186] Abdel Bar F.M., Abbas G.M., Gohar A.A., Lahloub M.F.I. (2020). Antiproliferative activity of stilbene derivatives and other constituents from the stem bark of *Morus nigra* L.. Nat. Prod. Res..

[187] Zhijun G., Jingjing Y., Minzhu N., Xinyue L., Jinran S., Yike L., Xinyu Y., Yulu Z., Xiaofeng Z., Jianguo H. (2024). Kuwanon G inhibits growth, migration and invasion of gastric cancer cells by regulating the PI3K/AKT/mTOR pathway. J. South. Med. Univ..

[188] Fan Y.F., Zhu S.X., Hou F.B., Zhao D.F., Pan Q.S., Xiang Y.W., Qian X.K., Ge G.B., Wang P. (2021). Spectrophotometric assays for sensing tyrosinase activity and their applications. Biosensors.

[189] El-Nashar H.A.S., Gamal El-Din M.I., Hritcu L., Eldahshan O.A. (2021). Insights on the inhibitory power of flavonoids on tyrosinase activity: A survey from 2016 to 2021. Molecules.

[190] Jin W., Stehbens S.J., Barnard R.T., Blaskovich M.A.T., Ziora Z.M. (2024). Dysregulation of tyrosinase activity: A potential link between skin disorders and neurodegeneration. J. Pharm. Pharmacol..

[191] Bai Y., Chen L., Cao Y.F., Hou X.D., Jia S.N., Zhou Q., He Y.Q., Hou J. (2021). Beta-glucuronidase inhibition by constituents of mulberry bark. Planta Med..

[192] Wei B., Yang W., Yan Z.X., Zhang Q.W., Yan R. (2018). Prenylflavonoids sanggenon C and kuwanon G from mulberry (*Morus alba* L.) as potent broad-spectrum bacterial β-glucuronidase inhibitors: Biological evaluation and molecular docking studies. J. Funct. Foods.

[193] Chen X., Han Y., Chen L., Tian Q.L., Yin Y.L., Zhou Q., Zang S.Z., Hou J. (2022). Discovery and characterization of the flavonoids in Cortex Mori Radicis as naturally occurring inhibitors against intestinal nitroreductases. Chem. Biol. Interact..

[194] Yao Z.J., Dong J., Che Y.J., Zhu M.F., Wen M., Wang N.N., Wang S., Lu A.P., Cao D.S. (2016). TargetNet: A web service for predicting potential drug–target interaction profiling via multi-target SAR models. J. Comput. Aided. Mol. Des..

[195] Davis A.P., Wiegers T.C., Sciaky D., Barkalow F., Strong M., Wyatt B., Wiegers J., McMorran R., Abrar S., Mattingly C.J. (2025). Comparative Toxicogenomics Database’s 20th anniversary: Update 2025. Nucleic Acids Res..

[196] Daina A., Michielin O., Zoete V. (2019). SwissTargetPrediction: Updated data and new features for efficient prediction of protein targets of small molecules. Nucleic Acids Res..

[197] Stelzer G., Rosen N., Plaschkes I., Zimmerman S., Twik M., Fishilevich S., Iny Stein T., Nudel R., Lieder I., Mazor Y. (2016). The GeneCards suite: From gene data mining to disease genome sequence analyses. Curr. Protoc. Bioinform..

[198] Piñero J., Corvi J., Rykova N., Guillem A., Martínez A., Zufiaur J.T., Rivetta I., Holmes S., Del Boccio M., Shapoval D. (2026). DISGENET: Accelerating data-driven discovery in disease genomics and therapeutic development. bioRxiv.

[199] Heberle H., Meirelles V.G., da Silva F.R., Telles G.P., Minghim R. (2015). InteractiVenn: A web-based tool for the analysis of sets through Venn diagrams. BMC Bioinform..

[200] Szklarczyk D., Kirsch R., Koutrouli M., Nastou K., Mehryary F., Hachilif R., Gable A.L., Fang T., Doncheva N.T., Pyysalo S. (2023). The STRING database in 2023: Protein–protein association networks and functional enrichment analyses for any sequenced genome of interest. Nucleic Acids Res..

[201] Shannon P., Markiel A., Ozier O., Baliga N.S., Wang J.T., Ramage D., Amin N., Schwikowski B., Ideker T. (2003). Cytoscape: A software environment for integrated models of biomolecular interaction networks. Genome Res..

[202] Sherman B.T., Hao M., Qiu J., Jiao X., Baseler M.W., Lane H.C., Imamichi T., Chang W. (2022). DAVID: A web server for functional enrichment analysis and functional annotation of gene lists (2021 update). Nucleic Acids Res..

[203] Chen T., Liu Y.X., Chen T., Yang M., Fan S., Shi M., Wei B., Lv H., Cao W., Wang C. (2024). ImageGP 2 for enhanced data visualization and reproducible analysis in biomedical research. iMeta.

